# Effect of dehydration of Syrah grape berries on the aging potential of fortified sweet wines in Ningxia of China

**DOI:** 10.1016/j.fochx.2025.102197

**Published:** 2025-01-17

**Authors:** Xiaoxi Zhang, Kaixian Wang, Qun Wang, Zhenan Shi, Hélder Oliveira, Nuno Mateus, Fuliang Han

**Affiliations:** a College of Enology, Northwest A&F University, Yangling 712100, Shaanxi, China; b Requimte/Laqv, Chemistry and Biochemistry Department, Faculty of Sciences, University of Porto, Portugal; c Shaanxi Engineering Research Center for Viti-Viniculture, Northwest A&F University, Yangling 712100, Shaanxi, China; d Heyang Experimental Demonstration Station, Northwest A&F University, Weinan 715300, Shaanxi, China

**Keywords:** Dehydration, Syrah, Fortified sweet wine, Phenolic, Organoleptic property

## Abstract

Many of wines from western China suffer from the short lifespan of aging. This study was conducted to investigate the effects of grape dehydration on aging potential of fortified sweet wines. Compared with the control wine (CK), dehydration significantly raised the sugar, organic acids and acetic esters content of G20 and G40 wines (made by 20 % and 40 % weight loss grapes). Dehydration also enhanced the stability of anthocyanins and the color of wines. After 24 months of aging, G20 had the highest monomeric anthocyanin content, and the variations of *a*^*⁎*^, *b*^*⁎*^ and *H*^*⁎*^_*ab*_ values were lowest in G40. Furthermore, dehydrated samples presented better aging stability with the improved sensory characteristics such as stronger black fruit fragrance, fuller body and richer aftertaste. In general, Syrah fortified sweet wine provides a new option for high quality wines with stable color, concentrated aroma, rich taste, and high aging potential in Ningxia of China.

## Highlights

• Dehydration enhances color stability and aroma intensity of wine during bottle aging.
• Dehydration improves aging ability and sensory property of Syrah fortified sweet wine.
• Alcohols and fatty acid ethyl esters contribute to smoky, sweet and nutty aromas.
• Providing a new option for high quality fortified sweet wines in Ningxia of China.

## Graphical abstract

**Figure:**
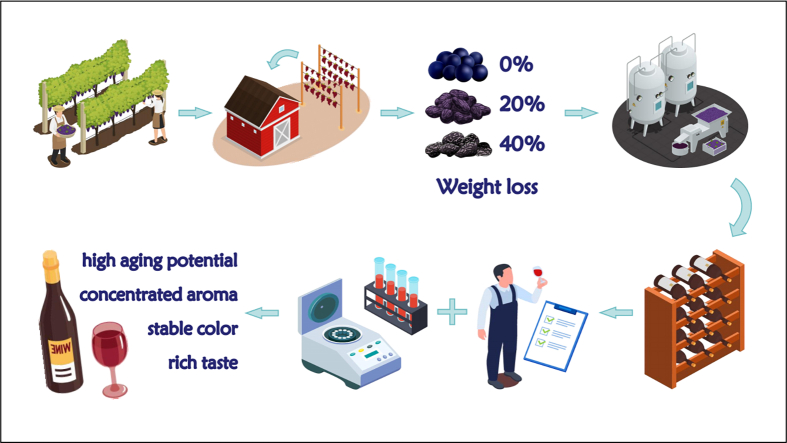

## 1 Introduction

Currently, red wines from northwestern wine-producing areas of China, including Ningxia, generally suffer from color lightening, aroma dissipating and taste fading during storage. Therefore, finding ways to enhance the aging quality of red wines is key to promoting the development of the wine industry. The aging quality of a wine refers to the quality produces or displays during the aging process, and the aging potential can be measured from three dimensions of quality change (Aleixandre-Tudo & du Toit, 2020). The first is the time dimension, which is judged in terms of how long it takes for a wine to reach its optimal sensory quality. The second is the quality dimension, which refers to the degree to which a wine reaches its optimal organoleptic quality after aging. The last dimension is the stability dimension, which is judged from the duration of the period of the wine's optimal sensory quality. The bottle aging occurs under reductive conditions and without other components being infiltrated, so the aging quality of wines at this stage is mainly determined by their initial composition before aging (Burin et al., 2011), and their aging potential can be better assessed in terms of the stability dimension. Normally, wines with high aging potential are characterized by high contents of sugar, acidity, phenolics or alcohol. Therefore, some fortified wines, especially fortified dessert wines, usually have great quality and aging potential, such as Port and Madeira wines in Portugal and Sherry in Spain (Perestrelo et al., 2023).

Sugar concentration technologies were most commonly used in the production of sweet wines, including noble-rot wine, icewine and raisin wine (Modesti et al., 2023). Due to the use of different winemaking technologies, the residual sugar and alcohol content of various sweet wine vary. For instance, icewine typically has a sugar content of over 125 g/L and an alcohol content of 5.5–14.9 %vol, whereas fortified sweet wines have a sugar content of 25 - 110 g/L and an alcohol content of 16.0–22.0 %vol (Andrade et al., 2001; Hong et al., 2021). The fortified sweet wines produced by the dehydrated grapes have a high alcohol content and a unique phenolic and aromatic profile (Panceri et al., 2015). Grape dehydration affects the metabolism and oxidation of volatile compounds and polyphenols in grapes, which has a great impact on the sensory quality and aging capacity of fortified sweet wines (Bai, Zhao, et al., 2023). Specifically, wines produced with dehydrated grapes are rich in polymeric anthocyanins and poor in monomeric anthocyanins, which contribute to color stabilization and have a positive impact on improving the aging ability of red wines (Aleixandre-Tudo & du Toit, 2020). Moreover, wines made from dehydrated grapes have higher levels of terpenes, norisoprenoids, lactones, aldehydes, and ketones, increasing the complexity and elegance of the aroma (Moreno et al., 2008). However, higher sugar musts could cause a stress response in yeast, resulting in the development of heightened quantities of yeast metabolites such as acetic acid (Teng et al., 2020). Thus, the volatile acidity in sweet wines is often between 0.5 and 1.5 g/L, while the titratable acidity is typically about 6 g/L (Bely et al., 2008; Hong et al., 2021).

Several factors contribute to the quality of fortified sweet wines with dehydrated grape, including grape varieties, dehydration approach and degree (Scalzini et al., 2023). They directly influence the profile of chemical compositions in grape berries, which in turn result in the difference of the characteristics and aging ability of fortified sweet wines (Ossola et al., 2017). For instance, the color of wines made from slow withered Avanà and Chatus grapes is darker (lower *L*^*∗*^ values and higher color intensity), while the trend is reversed in Nebbiolo wines (Torchio et al., 2016). In previous studies, we have investigated the impact of postharvest grape dehydration on Cabernet Sauvignon and Matheran wines. The results verifies that wines made from dehydrated grapes possess the better potential for enhancing the stability of wine quality during aging (Bai et al., 2024; Bai, Zhao, et al., 2023). However, few systematic studies have been reported on the effects of dehydration on the quality of Syrah fortified sweet wines. Thus, we produced fortified sweet wines by fresh and dehydrated Syrah grapes, to investigate whether fortified wines made from dehydrated grapes have better sensory characteristics and aging potential.

## 2 Materials and methods

### 2.1 Grape samples

Red grapes of the cv. ‘Syrah’ (*Vitis vinifera* L*.*) grown in Ningxia Province of China were harvested at the technological ripening stage in September 2020. The sugar content of the must was about 260.75 g/L, and the total acidity was 4.17 g/L. Bunches of grapes were subjected to postharvest dehydration under natural room conditions (18–23 °C, 53 %–71 % relative humidity) in order to dehydration. And healthy berries were collected at 0 %, 20 %, and 40 % weight loss for winemaking, labeled P0, P20 and P40, respectively (Fig. S1). The sugar content of P20 and P40 musts was approximately 330.34 g/L and 458.50 g/L, and the total acidity was 6.13 g/L and 6.67 g/L, respectively.

### 2.2 Wine samples

At Northwest A&F University's experimental winery, P0, P20 and P40 grapes were used to make the wine samples, which were branded CK, G20, and G40. The vinification protocol was the same for all six tests. Following the destemming and crushing of the grapes, the must was pumped into glass fermentation tanks with a volume of 20 L. Each replicate added 100 mg/kg of sulfur dioxide and 30 mg/kg pectinase (Lafaze Fruit). After a 36-h maceration period at 18 ± 1 °C, 250 mg/kg of yeast (Zymaflore RX60) was added. And the fermentation was conducted at a controlled temperature (20 ± 1 °C). When the total alcohol content of wine samples reach 15 ± 1 %, the fermentation was terminated by adding 60 % (*v*/v) brandy. After pressing, brandy was added to raise the alcohol content to 20 % (v/v). Then the resulting wines were cold-stabilized at 4 ± 1 °C for three months before being finally bottled and stored in the cellar. Among the two years of bottle aging, each sample was investigated half-yearly; the five periods were marked as T0, T6, T12, T18, and T24.

### 2.3 Reagents

Analytical-grade chemicals, including tartaric acid, anhydrous sodium sulfate, sodium hydroxide, sodium chloride, and glucose, were purchased from Beijing Chemical Works (Beijing, China). Chromatographic-grade reagents, including formic acid, acetonitrile, and acetic acid, were purchased from Tianjin Kemiou Chemical Reagent Co., Ltd. (Tianjin, China) and Anhui Tedia High Purity Solvents Co., Ltd. (Anhui, China). The organic acid standards and phenolic standards were obtained from Shanghai Yuanye Bio-Technology Co., Ltd. (Shanghai, China) and Sigma-Aldrich (Shanghai, China).

### 2.4 General wine parameters

The contents of residual sugar, alcohol, titratable acidity, and volatile acidity were analyzed by means of the National Standard of the People's Republic of China GB/T 15038–2006 (General Administration of Quality Supervision, Inspection & Quarantine (AQSIQ) Standardization Administration of China (SAC), 2006). pH values were measured by an acidity meter.

### 2.5 Color parameters and phenolic compositions

#### 2.5.1 Measurement of CIELab color parameters

Each wine sample's CIELab color parameters were determined using the Wine Color Analyzer W100 after filtering by 0.45 μm polyether sulfone membranes (Bai et al., 2024). The feature color is drawn using the Color Tell tool through the CIELab parameters (*L*^*⁎*^, *a*^*⁎*^, and *b*^*⁎*^).

#### 2.5.2 Determination of total polyphenol

In accordance with the method of Bai et al. (2024), the wine was diluted tenfold, 0.2 mL of the diluted wine sample was taken in a 10 mL centrifuge tube, 5 mL of deionized water, 0.5 mL of Folin-Shoka, 1.5 mL of a 20 % sodium carbonate solution, and 2.8 mL of deionized water were added sequentially and mixed, and the reaction was left to stand at 20 °C and protected from light for 2 h. Absorbance values were measured at 765 nm, and deionized water was used instead of the wine samples as the blank. The results were expressed in mg/L gallic acid, and each wine sample was repeated three times.

#### 2.5.3 Characterization of anthocyanins content

The HPLC instrument (LC-20AT, Shimadzu, Japan) with a photodiode array detector (SPD-M20A, Shimadzu, China) was used to determine anthocyanins in wines according to the HPLC procedure described by Han et al. (2009). Chromatographic separation of anthocyanins was performed on a Synergi Hydro-RP C18 column (250 × 4.6 mm, 4 μm, Phenomenex, Torrance, CA, USA). The mobile phases were: A = formic acid / acetonitrile / water (1:4:32, *v*/v/v); B = formic acid / acetonitrile / water (1:20:16, v/v/v), with the following analysis conditions: flow rate of 1 mL/min; column temperature of 35 °C; injection volume of 20 μL; detection wavelength of 520 nm. The elution gradient of mobile phase B was the following: 0 % to 35 % from 0 to 45 min; 35 % to 100 % from 45 to 46 min; 100 % from 46 to 50 min; 100 % to 0 % from 50 to 51 min; and 0 % from 51 to 55 min.

#### 2.5.4 Characterization of non-anthocyanin phenolic content

The wine samples were analyzed using the previously outlined method (Chen et al., 2024). Collecting the supernatants of wine and ethyl acetate to evaporate and then redissolving them in 0.5 mL of methanol for HPLC analysis. Determined the samples by an LC-20AT HPLC instrument coupled with the SPD-M20A photodiode array detector and using the Synergi Hydro-RP C18 column (250 × 4.6 mm, 4 μm) to separate the components of phenolic acids. The mobile phases, mobile phase A (0.1 % acetic acid in an 8:1 *v*/v water:acetonitrile mixture) and mobile phase B (0.1 % acetic acid in a 4:5 v/v water:acetonitrile mixture), had a flow rate of 1 mL/min. The relevant conditions were set as follows: column temperature, 30 °C; detection wavelength, 280 nm; injection volume, 20 μL. And the gradient program was configured as follows: 100 to 65 % A from 0 to 45 min; 65 to 0 % A from 45 to 50 min; 0 % A from 50 to 55 min; 0 % to 100 % A from 55 to 56 min; and 100 % A from 56 to 62 min. Using the external standard method to quantify the phenolic acid content.

### 2.6 Organic acids

After wine samples were filtered through 0.22 μm polyethersulfone filters, the organic acid analysis was performed by the HPLC instrument (LC-20AT, Shimadzu, Japan) coupled with a photodiode array detector (SPD-M20A, Shimadzu, China) directly (Chen et al., 2024). HPLC system equipped with Hydro-RP C18 column (250 × 4.6 mm, 4 μm, Phenomenex, Torrance, CA, USA), and the mobile phase was 0.05 mmol/L H_2_SO_4_ aqueous solution with 0.45 mL/min. The injection volume was 5 μL, and the column temperature was 55 °C.

### 2.7 Volatile compounds

The analysis of volatile compounds in wines was done by headspace - SPME - gas chromatography–mass spectrometry (HS-SPME-GC–MS), referring to the method of Liu et al. (2024). The sample was incubated at 40 °C for 15 min and extracted for 30 min, then transferred to the injector for desorption at 230 °C for 5 min. During the GC run, a constant flow rate (1.5 mL/min) of the carrier gas (helium) was maintained. The injector temperature was held at 250 °C. The oven temperature was programmed to 40 °C for 5 min, then raised to 130 °C and 220 °C at the rates of 2 °C/min and 5 °C/min, separately. Finally, it was kept at 220 °C for 10 min. GC–MS spectra were produced by electron ionization (EI) with an electron energy of 70 eV and a mass range of 35–350 amu.

Qualitative analysis was conducted by using standard retention time comparisons, retention index comparisons, and NIST 17.0 mass spectral library queries. The internal standard calibration curve method was used for quantification.

### 2.8 Organoleptic properties

In accordance with the method described by Bai, Zhang, et al. (2023), the organoleptic properties analysis of wine samples was performed by 12 oenology students attending the College of Enology of Northwest A&F University (comprising 8 females and 4 males). All the judges were selected based on their basic sensory performances during 8-week training, which were determined by the reproducibility index (Ri > 2), the F value of the test statistics, the mean-square error (MSE), and the Manhattan plots (Tomic et al., 2013). Due to the limited number of samples, we only analyzed samples from the T0, T12 and T18 periods.

In the formal sensory evaluation of wine samples, each judge was required to employ quantitative descriptive analysis to evaluate the appearance (including purity, lightness, and color), aroma (including condition, elegance, and intensity), and taste (including balance, characteristics, length, body, alcohol, tannin, bitterness, and sweetness) of the wines, describe the aroma attributes of the wines using three to five aroma descriptors, and rate the intensity of each descriptor. Each descriptor was quantified using a 5-point intensity scale, and using the M-value to express wine aroma quantification (Tao & Zhang, 2010).

### 2.9 Statistical analysis

One-way ANOVA was conducted using SPSS (IBM SPSS Statistics 23.0), with significant differences established by Tukey's test (*p* < 0.05). The graphs were performed using GraphPad Prism version 8.00 for Windows (GraphPad Software, California, USA) and OriginPro 2024 (OriginLab Corporation, USA). The results of Correlation analysis and Mantel test were performed using ChiPlot (https://www.chiplot.online/).

## 3 Results and discussion

### 3.1 Main chemical parameters

The main chemical parameters of Syrah fortified sweet wines after 0, 6, 12, 18, and 24 months of bottle aging are displayed in Table 1, which could provide a visual indication of the quality of wines. The results showed that G20 and G40 with the same alcohol level, the reducing sugar content and the acidity tended to grow considerably (*p* < 0.05): the G40 sample's reducing sugar concentration and volatile acid content were about 3 and 5 times higher than those of CK, respectively.

**Table 1:** The main chemical parameters of Syrah fortified sweet wines made by fresh and dehydrated grapes at 0, 6, 12, 18, and 24 months of bottle aging.

Time	Group	Alcohol content (%Vol)	Residual sugar (g/L)	Titratable acidity (g/L)	Volatile acidity (g/L)
T0	CK	19.53 ± 0.26a	54.75 ± 0.19c	5.58 ± 0.08a	0.28 ± 0.02c
	G20	18.63 ± 1.46a	64.69 ± 5.20b	5.82 ± 0.00a	0.52 ± 0.02b
	G40	19.16 ± 0.72a	166.37 ± 10.39a	6.21 ± 0.66a	1.53 ± 0.03a
T6	CK	19.74 ± 0.84a	51.79 ± 2.80c	5.76 ± 0.09b	0.29 ± 0.01c
	G20	18.94 ± 0.94a	60.29 ± 0.77b	6.03 ± 0.16b	0.56 ± 0.01b
	G40	20.16 ± 1.43a	161.74 ± 8.80a	6.34 ± 0.11a	1.52 ± 0.02a
T12	CK	19.89 ± 0.25a	48.29 ± 2.81c	5.66 ± 0.02b	0.30 ± 0.00c
	G20	19.36 ± 0.36a	56.29 ± 0.77b	5.86 ± 0.14b	0.58 ± 0.01b
	G40	19.85 ± 0.95a	152.99 ± 6.17a	6.56 ± 0.15a	1.51 ± 0.02a
T18	CK	19.85 ± 0.84a	44.40 ± 2.41c	5.59 ± 0.21c	0.30 ± 0.01c
	G20	18.96 ± 1.04a	52.43 ± 3.78b	6.00 ± 0.17b	0.64 ± 0.03b
	G40	19.48 ± 0.93a	141.89 ± 5.28a	7.03 ± 0.21a	1.53 ± 0.09a
T24	CK	19.58 ± 0.75a	42.94 ± 2.44c	5.48 ± 0.08c	0.34 ± 0.01c
	G20	19.03 ± 1.05a	50.41 ± 1.35b	5.92 ± 0.15b	0.64 ± 0.00b
	G40	19.67 ± 0.85a	133.91 ± 5.09a	6.83 ± 0.13a	1.46 ± 0.02a

For every bottle aging period, a distinct statistical analysis was carried out. Different letters indicate significant differences among wine samples (*p* < 0.05). Wine samples coding: CK, the control, made by 0 % weight loss grapes (P0); G20, made by 20 % weight loss grapes (P20); G40, made by 40 % weight loss grapes (P40).

The increase in titratable acidity may be the result of the concentration effect from dehydration and the rise in volatile acid content (Panceri & Bordignon-Luiz, 2017). During two years of aging, there was a general tendency for the reducing sugar concentration to gradually fall, possible due to the Maillard reaction of sugar and amino acids (Lopez de Lerma et al., 2010). The increase in volatile acid content may be attributed to the enrichment of sugars due to dehydration, which imposes osmotic stress on the cells of *Saccharomyces cerevisiae*, and in response to the stress, some of the pyruvic acid is converted to glycerol and acetic acid (Kelly et al., 2018). This phenomenon also occurs in other wines made from high-sugar must, such as icewines and Passito wines (Rantsiou et al., 2012). Furthermore, high sugar also leads to a decrease in yeast activity during the fermentation, which is one of the main problems in the production of sweet wines (Cortes et al., 2010). In this work, as the degree of dehydration of the grapes and the sugar content of the must increased, the fermentation performed longer, while the rate of carbon dioxide production decreased.

### 3.2 Organic acids

**Fig. 1:**
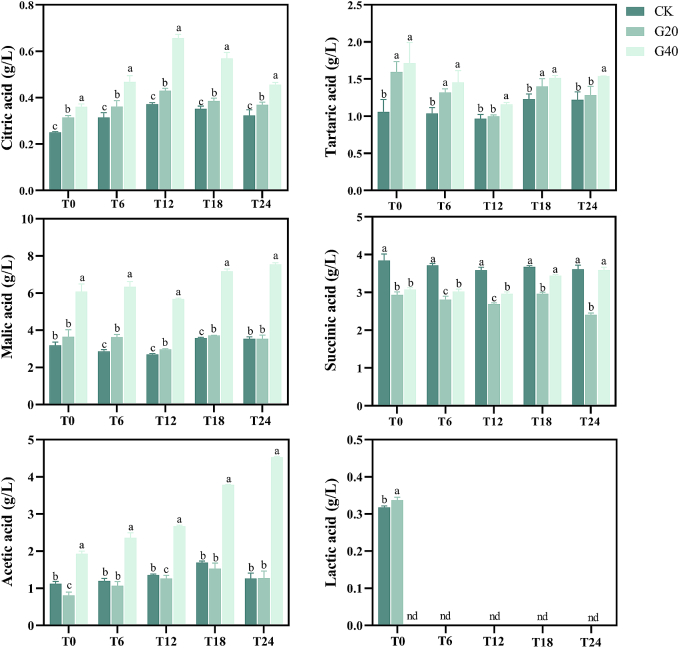
The organic acids content of Syrah fortified sweet wines made by fresh and dehydrated grapes at 0, 6, 12, 18 and 24 months of bottle aging (coding: T0; T6; T12; T18; T24). For every bottle aging period, a distinct statistical analysis was carried out. Different letters indicate significant differences among wine samples (*p* < 0.05), “nd” indicate not detected. Wine samples coding: CK, the control, made by 0 % weight loss grapes (P0); G20, made by 20 % weight loss grapes (P20); G40, made by 40 % weight loss grapes (P40).

The predominant organic acids identified in fortified sweet wines were tartaric, malic, and succinic acids, each with a content of more than 1 g/L (Fig. 1). Like the results of Panceri and Bordignon-Luiz (2017), the organic acids contents were significantly affected by grape dehydration degree. During the aging process, the contents of citric acid, tartaric acid, malic acid and acetic acid were considerably higher in G40 wine than in CK and G20 wines (*p* < 0.05). This is due to the fact that the water loss of berries during the dehydration, which concentrated the components of must. It is noteworthy that organic acids can protect human immunological and cardiovascular systems by regulating metabolism and supplying energy (Shi et al., 2022). Furthermore, organic acids can improve the stability of color and aroma of aging wines (Bai et al., 2024). Therefore, the increase of organic acids contents may modulate the organoleptic properties and nutritive value of Syrah fortified sweet wines.

Moreover, succinic acid content tends to drop down and then increase as the dehydration degree increases. Lactic acid was only present in CK and G20 at T0 and not detected in G40, indicating that wines did not undergo malolactic fermentation and the fortified treatment may have restricted *Lactobacillus* activity. During the aging process, the succinic acid content remains generally constant. The amount of acetic acid and citric acid increased as wine samples aged. Conversely, lactic acid, malic acid, and tartaric acid contents were shown to be lower. This is most likely caused by the crystallization of tartrate, the involvement of malic acid in oxidative breakdown of cells, and the reaction of lactic acid with ethanol to make ethyl lactate (Conde et al., 2018).

### 3.3 Phenolic compounds

#### 3.3.1 Total polyphenol

**Fig. 2:**
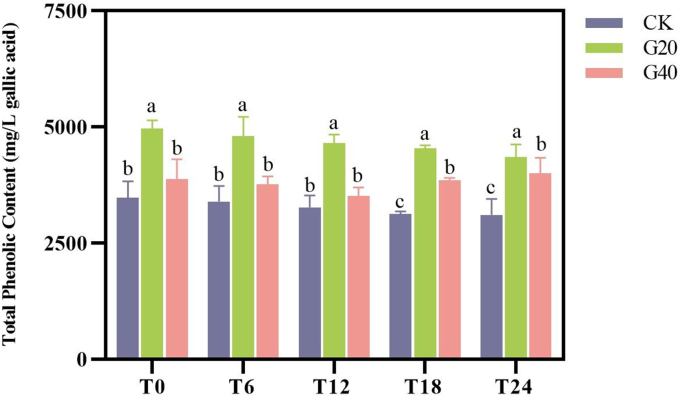
The total phenolic content of Syrah fortified sweet wines made by fresh and dehydrated grapes at 0, 6, 12, 18, and 24 months of bottle aging (coding: T0; T6; T12; T18; T24). For every bottle aging period, a distinct statistical analysis was carried out. Different letters indicate significant differences among wine samples (*p* < 0.05). Wine samples coding: CK, the control, made by 0 % weight loss grapes (P0); G20, made by 20 % weight loss grapes (P20); G40, made by 40 % weight loss grapes (P40).

As illustrated in Fig. 2, G20 wines were significantly higher in total polyphenol than CK and G40 wines (*p* < 0.05) during the two-year aging, indicating that G20 wines may have a stronger aging potential. Specifically, the highest total phenolic content was in G20 (4959.21 ± 181.37 mg/L) at T0 and the lowest was in CK (3101.17 ± 345.59 mg/L) at T24, which shows that dehydration treatment increases the total phenolic content of fortified sweet wines, but over-hydration leads to a decrease. The increase in total phenolic content of dehydrated wines can be explained by the abiotic stress caused by water loss, which induces metabolic pathways for phenolic synthesis (Mencarelli et al., 2021). On the other hand, the rupture of grape pericarp cells caused by grape dehydration also had a strengthening effect on pre-fermentation maceration (Ossola et al., 2017).

#### 3.3.2 Monomeric anthocyanins

**Fig. 3:**
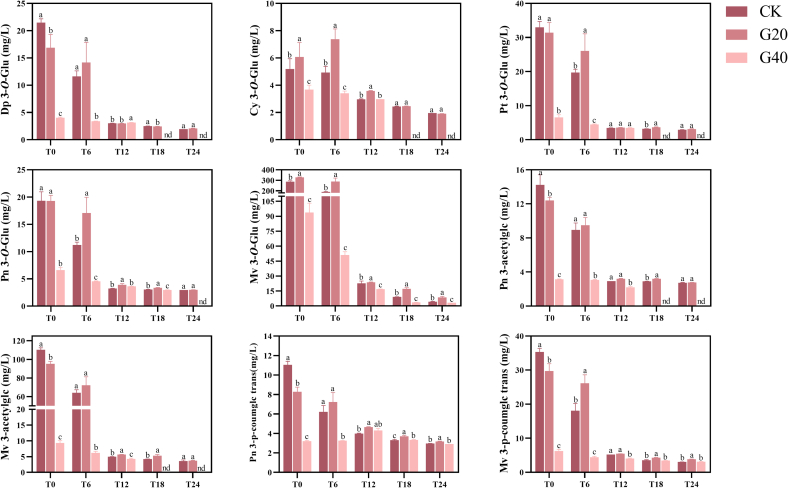
The monomeric anthocyanin content of Syrah fortified sweet wines made by fresh and dehydrated grapes at 0, 6, 12, 18 and 24 months of bottle aging (coding: T0; T6; T12; T18; T24). For every bottle aging period, a distinct statistical analysis was carried out. Different letters indicate significant differences among wine samples (*p* < 0.05), nd indicate not detected. Wine samples coding: CK, the control, made by 0 % weight loss grapes (P0); G20, made by 20 % weight loss grapes (P20); G40, made by 40 % weight loss grapes (P40). Abbreviations: delphinidin-3-*O*-glucoside (Dp 3-*O*-Glu); cyanidin-3-*O*-glucoside (Cy 3-*O*-Glu); petunidin-3-*O*-glucoside (Pt 3-*O*-Glu); peonidin-3-*O*-glucoside (Pn 3-*O*-Glu); malvidin-3-*O*-glucoside (Mv 3-*O*-Glu); peonidin-3-*O*-(6-*O*-acetyl)-glucoside (Pn-3-acetylglc); Malvidin-3-*O*-(6-*O*-acetyl)-glucoside (Mv-3-*O*-acetylglc); trans-peonidin-3-*O*-(6-*O*-p- coumaryl)-glucoside (Pn-3-p-coumglc trans); trans-Malvidin-3-*O*-(6-*O*-p- coumaryl)-glucoside (Mv-3-p-coumglc trans).

Syrah fortified sweet wines were found to contain nine distinct monomeric anthocyanins (Fig. 3). As the primary component, Malvidin-3-*O*-glucoside (Mv 3-*O*-Glu) takes the largest proportion, followed by Malvidin-3-*O*-(6-*O*-acetyl)-glucoside (Mv-3-*O*-acetylglc). Cyanidin-3-*O*-glucoside (Cy 3-*O*-Glu) exhibited the lowest concentration. With the exception of Cy 3-*O*-Glu and Mv 3-*O*-Glu, the remaining monomeric anthocyanins in both G20 and G40 wines were lower than in CK wine, and the magnitude of change was significantly higher in G40 than in G20. This is attributed to the fact that the drying treatment of the grapes induced thermal, oxidative degradation, cycloaddition or polymerization reactions of anthocyanins to produce other phenolics with a more structurally stable structure, such as colored polymers form anthocyanins with tannins (Marquez et al., 2013).

Observe the bottle aging process, the monomeric anthocyanin content of all three wines falls sharply, but the magnitude of the decrease had variations. In concrete terms, the contents of all monomeric anthocyanins in G40 wine were significantly lower than those in CK and G20 wines, with Delphinidin-3-*O*-glucoside (Dp 3-*O*-Glu), Cy 3-*O*-Glu, Petunidin-3-O-glucoside (Pt 3-*O*-Glu), Peonidin-3-*O*-(6’-*O*-acetyl)-glucoside (Pn-3-acetylglc), and Mv-3-*O*-acetylglc no longer identifiable in G40 after 18 months. The contents of the seven monomeric anthocyanins of G20 were lower than that of CK at the period of T0, but were higher than that of CK after six months of aging. Studies have demonstrated that the decline in monomeric anthocyanin concentration throughout aging is caused by oxidative degradation and the synthesis of derivatives with other acids and phenolics (Figueiredo-Gonzalez et al., 2013). In addition, the Cy 3-*O*-Glu and Mv 3-*O*-Glu contents of G20 were significantly higher than those of CK (*p* < 0.05) at both T0 and T6. This suggests that while the dehydration treatment causes monomeric anthocyanins to be degraded by oxidation, it also causes more monomeric anthocyanins to be extracted from the dehydrated wine because the pericarp cells rupture during the dehydration process (Fasoli et al., 2019).

Overall, the three wines were characterized by considerable differences in monomeric anthocyanin content due to the different dehydration degrees. After bottling, CK had the highest monomeric anthocyanin content, but along with aging, G20 had a higher monomeric anthocyanin content than G40 and CK. This result is consistent with our prior work on Marselan grapes (Chen et al., 2024), indicating that moderate drying of grapes can enhance the stability of monomeric anthocyanins in Syrah fortified sweet wines, which has a positive effect on the maintenance of a vivid red color and improvement of aging potential (He et al., 2012).

#### 3.3.3 Non-anthocyanin phenolics

**Fig. 4:**
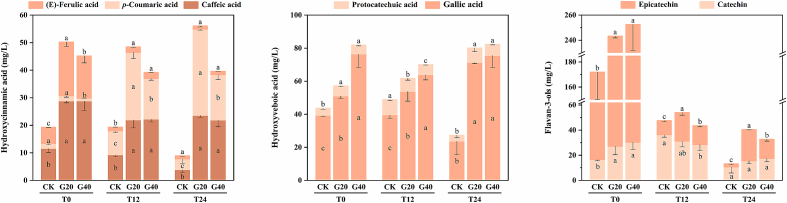
The non-anthocyanin phenolic content of Syrah fortified sweet wines made by fresh and dehydrated grapes at 0, 12, and 24 months of bottle aging (coding: T0; T12; T24). For every bottle aging period, a distinct statistical analysis was carried out. Different letters indicate significant differences among wine samples (*p* < 0.05). Wine samples coding: CK, the control, made by 0 % weight loss grapes (P0); G20, made by 20 % weight loss grapes (P20); G40, made by 40 % weight loss grapes (P40).

As shown in Fig. 4, the concentrations of non-anthocyanin phenolics in the three types of fortified sweet wines were highly influenced by the degree of grape drying, and this effect persisted during bottle aging.

Probably because hydroxycinnamates are hydrolyzed to the corresponding free phenolic acids by cinnamoyl esterases during dehydration (Panceri et al., 2015). G20 and G40 had significantly greater levels of caffeic acid and (*E*)-Ferulic acid than CK, whereas G20 showed the greatest levels of *p*-coumaric acid. Hydroxybenzoic acids also have the same trend as hydroxycinnamic acids, including protocatechuic acid and gallic acid. It has been reported that both hydroxybenzoic acids and hydroxycinnamic acids are important co-factors, which can interact with anthocyanins to form stable copigmentation complexes. They can significantly increase the color stability and produce hyperchromic effect and bathochromic effect by changing the structures of anthocyanins (He et al., 2012). In addition, gallic acid can be added in wine as hydrolysates of tannin to enhance the aging potential of red wine.

In agreement with the previous literature (Panceri et al., 2013), fortified sweet wines made from the dehydrated grapes have higher total flavan-3-ols content. The main manifestation was that the epicatechin content was significantly higher than CK at all stages of bottle aging (*p* > 0.05). The increase in flavan-3-ols content is one of the main metabolic activities of the postharvest dehydration of grape berries, which could stabilize anthocyanins and increase the astringency and color of wines (Bonghi et al., 2012). Overall, wines made using dehydrated grapes may have better color characteristics and aging potential.

### 3.4 CIELab parameters and feature color

Color is the most crucial indicator to influence consumer preference for red wine. From the results, it is clear that the CIELab parameters and feature color of wines were markedly influenced by dehydration (Table 2). Sweet wines made from dehydrated grapes have a more pleasing and stable color.

**Table 2:**
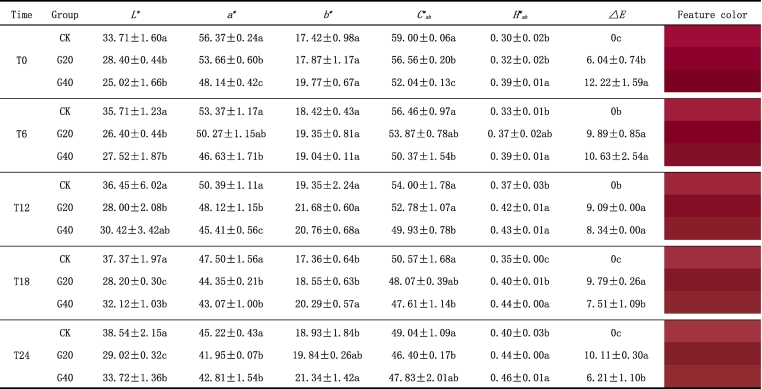
The CIELab parameters and feature color of Syrah wines made by fresh and dehydrated grapes at 0, 6, 12, 18 and 24 months of bottle aging.

For every bottle aging period, a distinct statistical analysis was carried out. Different letters indicate significant differences among wine samples (*p* < 0.05). Wine samples coding: CK, the control, made by 0 % weight loss grapes; G20, made by 20 % weight loss grapes; G40, made by 40 % weight loss grapes.

After bottling (T0), G40 and G20 wines revealed significantly higher *H*^*⁎*^_*ab*_ (hue angle) values and lower values of *L*^*⁎*^ (lightness), *a*^*⁎*^ (red-green color channel) and *C*^*⁎*^_*ab*_ (chroma or saturation) in comparison to CK wine (*p* < 0.05). Correspondingly, the color difference (*ΔE*) of G20 and G40 wines reached 6.04 and 12.22, which were both above 3.0. This means that the effect of dehydration of grape on the wine color could be visually perceptible (Fanzone et al., 2015). Besides, except for the *L*^*⁎*^ value, the evolution of color parameters was consistent with changes during wine aging, which means the grape dehydration process could cause visually aging-like changes in the feature color, while the color of G40 and G20 wines is deeper. This is probably caused by the oxidation and formation of anthocyanin derivatives and the interaction between phenolic species (Li, Prejano, et al., 2019).

It is worth noting that, the wines with longer bottle aging had higher *L*^*⁎*^, *H*^*⁎*^_*ab*_ and *b*^*⁎*^ values but lower *a*^*⁎*^ and *C*^*⁎*^_*ab*_ values, suggesting amore yellowish tonality. A further novel finding is that the variations of *a*^*⁎*^, *b*^*⁎*^, *C*^*⁎*^_*ab*_ and *H*^*⁎*^_*ab*_ values were lower in G40 wine than in G20 and CK wines (Table 3)*.* Conversely, the variation of *L*^*⁎*^ value shows the opposite trend. Altogether, the results suggest that the using of dehydrated grapes may help to slow down the color degradation of Syrah fortified sweet wines during bottle aging, which ties well with previous studies (Bai et al., 2024).

**Table 3:** The variations of CIELab parameters of Syrah wines made by fresh and dehydrated grapes at 0, 6, 12, 18 and 24 months of bottle aging.

Time	Group	Variation (%)				
		*L*^*⁎*^ (%)	*a*^*⁎*^ (%)	*b*^*⁎*^ (%)	*C*^*⁎*^_*ab*_ (%)	*H*^*⁎*^_*ab*_ (%)
T0	CK	0.00	0.00	0.00	0.00	0.00
	G20	0.00	0.00	0.00	0.00	0.00
	G40	0.00	0.00	0.00	0.00	0.00
T6	CK	5.93	−5.32	5.74	−4.31	10.00
	G20	−7.04	−6.32	8.28	−4.76	15.63
	G40	9.99	−3.14	−3.69	−3.21	0.00
T12	CK	7.67	−11.20	10.48	−8.86	21.21
	G20	−1.52	−11.02	19.69	−7.02	27.03
	G40	19.62	−5.85	5.20	−4.19	10.26
T18	CK	10.04	−17.60	−0.31	−15.61	13.51
	G20	−0.71	−19.35	3.14	−16.09	19.05
	G40	23.34	−11.16	2.50	−8.87	11.63
T24	CK	12.92	−23.47	8.70	−19.70	28.57
	G20	2.20	−26.40	10.62	−21.14	30.00
	G40	27.09	−12.38	7.74	−8.84	15.91

All calculations of the variations of CIELab parameters are based on T0 period. Wine samples coding: CK, the control, made by 0 % weight loss grapes; G20, made by 20 % weight loss grapes; G40, made by 40 % weight loss grapes.

### 3.5 Wines volatile compositions

A total of 29 volatile components were detected in fortified sweet wines, including 3 acetic esters, 12 fatty acid ethyl esters, 9 alcohols, 3 acids, and 2 aldehydes (Table 4). Fatty acid ethyl esters and alcohols were the main volatile components of fortified sweet wines. It is worth noting that the dehydrated samples showed higher contents of acetate, fatty acid ethyl ester and aldehydes, and lower contents of alcohols (*p* < 0.05). Fatty acid ethyl esters were the main contributors to the floral and fruity aromas of the wines (Peng et al., 2013). Except for ethyl 3-methylbutanoate, ethyl lactate, and diethyl succinate, the contents of all other fatty acid ethyl esters of the dehydrated-treated fortified sweet wines were significantly higher than those of the CK wine samples (*p* < 0.05), which indicated that the dehydration treatment would give the wines a more intense floral and fruity aroma. Among the alcohols, moderate amounts of 2-Methyl-1-propanol and 3-Methyl-1-butanol had a whisky aroma but an irritating nail polish odor at high concentrations (Arcari et al., 2017). The content of 3-Methyl-1-butanol decreased significantly (*p* < 0.05) with the increase in the degree of dehydration, while the 2-Methyl-1-propanol content appeared to have a tendency to decrease first and then to increase, which suggests that a certain degree of dehydration can improve the aroma quality of fortified sweet wines.

**Table 4:** The volatile compounds of Syrah fortified sweet wines made by fresh and dehydrated grapes before bottle aging.

Compound (μg/L)	Odor threshold(μg/L) a	CK		G20		G40		Odor descriptor c
		Concentration (μg/L)	OAV b	Concentration (μg/L)	OAV b	Concentration (μg/L)	OAV b	
*Acetic esters*		77,843.4 ± 861.19c		107,145.81 ± 4096.38b		392,148.13 ± 14,694.40a		
Ethyl acetate	75000^1^	77,565.33 ± 844.24c	1.03	106,804.54 ± 4121.21b	1.03	391,458.14 ± 14,666.91a	5.22	fruity, solvent^2^
3-Methylbutyl acetate	30^3^	265.65 ± 27.84c	8.86	332.04 ± 25.23b	8.86	675.11 ± 26.09a	22.50	banana, fruity, sweet^4^
Phenethyl acetate	250^1^	12.42 ± 0.88b	0.05	9.23 ± 0.51c	0.05	14.88 ± 1.4a	0.06	flowers^2^

*Fatty acid ethyl esters*		28,183.22 ± 2047.36a		16,519.34 ± 483.02c		21,451.96 ± 1769.11b		
Ethyl 2-methylpropanoate	15^5^	8.34 ± 0.68b	0.56	6.22 ± 0.47c	0.56	11.33 ± 0.26a	0.76	fruity, banana^5^
Ethyl butyrate	20^6^	18.76 ± 0.46b	0.94	18.40 ± 0.68b	0.94	27.21 ± 1.53a	1.36	strawberry, apple^5^
Ethyl 2-methylbutanoate	1^1^	50.42 ± 4.82b	50.42	25.13 ± 0.96c	50.42	65.01 ± 3.17a	65.01	sweet fruit^5^
Ethyl 3-methylbutanoate	3^1^	20.52 ± 1.58a	6.84	12.75 ± 0.37c	6.84	16.09 ± 0.40b	5.36	berry, blackberry^5^
Ethyl hexanoate	5^1^	563.79 ± 61.54b	112.76	434.70 ± 5.97c	112.76	790.12 ± 51.63a	158.02	green apple^7^
Ethyl lactate	150000^8^	18,260.67 ± 1398.57a	0.12	10,993.20 ± 230.60b	0.12	9809.25 ± 410.34b	0.07	strawberry, raspberry^9^
Methyl octanoate	200^10^	1.11 ± 0.31b	0.01	0.92 ± 0.01b	0.01	3.71 ± 0.32a	0.02	apple skin, fruity^5^


Compound (μg/L)	Odor threshold(μg/L) a	CK		G20		G40		Odor descriptor c
		Concentration (μg/L)	OAV b	Concentration (μg/L)	OAV b	Concentration (μg/L)	OAV b	
Ethyl octanoate	580^8^	1435.08 ± 457.98b	2.47	907.04 ± 58.91b	2.47	3681.05 ± 358.65a	6.35	sweet, fruity^7^
Ethyl decanoate	200^11^	433.32 ± 200.80b	2.17	341.21 ± 24.81b	2.17	2000.78 ± 340.42a	10.00	fruity, grape^9^
Dithyl succinate	200000^12^	7370.49 ± 76.39a	0.04	3748.42 ± 353.81b	0.04	4257.00 ± 403.58b	0.02	vinous^7^
Ethyl dodecanoate	500^8^	3.45 ± 1.55b	0.01	3.83 ± 0.11b	0.01	45.09 ± 11.51a	0.09	candy, floral^9^
Ethyl hexadecanoate	1000^8^	20.10 ± 6.79b	0.02	29.83 ± 8.09b	0.02	750.98 ± 187.64a	0.75	wax, milk, cream^13^

***Alcohols***		510,293.82 ± 14,429.43a		343,973.21 ± 7065.27b		342,718.12 ± 23,779.42b		
2-Methyl-1-propanol	40000^3^	51,368.30 ± 1888.13b	1.28	43,041.46 ± 767.94c	1.28	66,615.65 ± 3825.79a	1.67	nf d
3-Methyl-1-butanol	30000^9^	300,482.23 ± 8653.48a	10.02	231,976.95 ± 5260.76b	10.02	228,331.63 ± 14,406.28b	7.61	nail polish, whiskey^9^
3-Methyl-1-pentanol	500^6^	3831.76 ± 190.35a	7.66	4045.24 ± 76.41a	7.66	2425.24 ± 180.27b	4.85	pungent,brandy,cocoa^2^
1-Hexanol	8000^1^	1268.64 ± 47.32a	0.16	1338.42 ± 42.99a	0.16	796.92 ± 58.79b	0.10	cut grass, wood^14^
2-Ethylhexanol	nf d	15.92 ± 0.16b		14.82 ± 0.83b		19.43 ± 0.72a		nf d
1-Octanol	800^15^	6.17 ± 0.04a	0.01	4.67 ± 0.08b	0.01	nd ^*e*^	nd ^*e*^	intense citrus, rose^16^
α-Terpineol	250^11^	2.60 ± 0.11a	0.01	nd ^*e*^	nd ^*e*^	3.45 ± 0.00a	0.01	pine, resinous, citrus^2^
3-Methylthio-1-propanol	500^9^	750.40 ± 154.60a	1.50	nd ^e^	nd ^*e*^	nd ^e^	nd ^*e*^	boiled, vegetables9
Phenylethyl alcohol	10000^14^	152,568.65 ± 4233.16a	15.26	63,551.65 ± 1864.35b	15.26	44,527.53 ± 5303.39c	4.45	rose^2^

Compound (μg/L)	Odor threshold(μg/L) a	CK		G20		G40		Odor descriptor c
		Concentration (μg/L)	OAV b	Concentration (μg/L)	OAV b	Concentration (μg/L)	OAV b	
*Acids*		182,994.82 ± 28,636.7c		308,174.58 ± 9022.5b		1,207,440.94 ± 102,154.73a		
Acetic acid	200000^1^	182,377.44 ± 28,619.52c	0.91	307,231.59 ± 8766.37b	0.91	1,206,606.85 ± 102,084.84a	6.03	acetic^1^
Isobutyric acid	2300^11^	nd ^*e*^	nd ^*e*^	511.62 ± 32.85a	0.00	nd ^*e*^	nd ^*e*^	cheese, butter rancid^16^
Isovaleric acid	33.4^11^	472.57 ± 28.12a	14.15	529.42 ± 11.29a	14.15	518.97 ± 36.82a	15.54	nf d

*Aldehydes*		7795.25 ± 2648.14ab		5256.83 ± 231.74b		11,074.34 ± 1078.37a		
Furfural	14100^8^	86.00 ± 0.69b	0.01	72.75 ± 3.23b	0.01	293.32 ± 19.97a	0.02	sweet, almond, bread^2^
Acetoin	30000^17^	7709.25 ± 2648.77bc	0.26	5184.08 ± 228.82c	0.26	10,781.03 ± 1058.41ab	0.36	butter^17^

Different letters indicate significant differences among wine samples (*p* < 0.05). Sample coding: CK, the control, made by 0 % weight loss grapes; G20, made by 20 % weight loss grapes; G40, made by 40 % weight loss grapes. Different letters indicate significant differences among wine samples (*p* < 0.05).

a Superscripts are references to the values taken. [1] Rotaru et al., 2010; [2] Duran-Guerrero et al., 2021; [3] Urcan et al., 2017; [4] Ribeiro et al., 2023; [5] Peng et al., 2013; [6] Tao & Zhang, 2010; [7] Garcia-Carpintero et al., 2011; [8] Fogarty, 2020; [9] Arcari et al., 2017; [10] Qian et al., 2020; [11] Song et al., 2023; [12] Lan et al., 2019; [13] Wang et al., 2018; [14] He et al., 2012; [15] Li, Guo, et al., 2019; [16] Chen et al., 2023; [17] Moyano et al., 2010.

b OAV, odor activity value, which is calculated by dividing the mean concentration of volatile compounds by odor thresholds.

c Superscripts are references to the descriptors taken.

d nf: not found.

In addition, the dehydrated samples had higher levels of acetic esters, especially ethyl acetate and 3-Methylbutyl acetate. Compared with CK, the ethyl acetate content in G20 and G40 increased 1.4-fold and 5.0-fold, respectively. And their OAV values were above 1, suggesting that they contribute significantly to the fruity and sweet flavors such as banana and pear of wines (Ribeiro et al., 2023). Furthermore, the amount of aldehydes exhibited a tendency to decrease and then increase with the degree of drying, the amount of furfural and acetoin in G40 was 3.4 and 1.40 times greater than in CK, respectively. Meanwhile, some undesired odorants were also found to be much more prevalent in dehydrated samples (*p* < 0.05). The content of acids increased significantly (*p* < 0.05), the acetic acid content in G20 and G40 increased 1.7-fold and 6.6-fold, which were lower than maximum allowable limit in OIV (2.1 g/L) (Hong et al., 2021). The reason for this was the higher sugar content in musts, which could cause stress on the yeast during fermentation and the development of heightened quantities of yeast metabolites such as acetic acid (Teng et al., 2020). These findings suggested that the aroma quality of the wines would benefit from a suitable degree of dehydration treatment, but that the degree of dehydration should be controlled to prevent potential negative effects.

### 3.6 Organoleptic properties

**Fig. 5:**
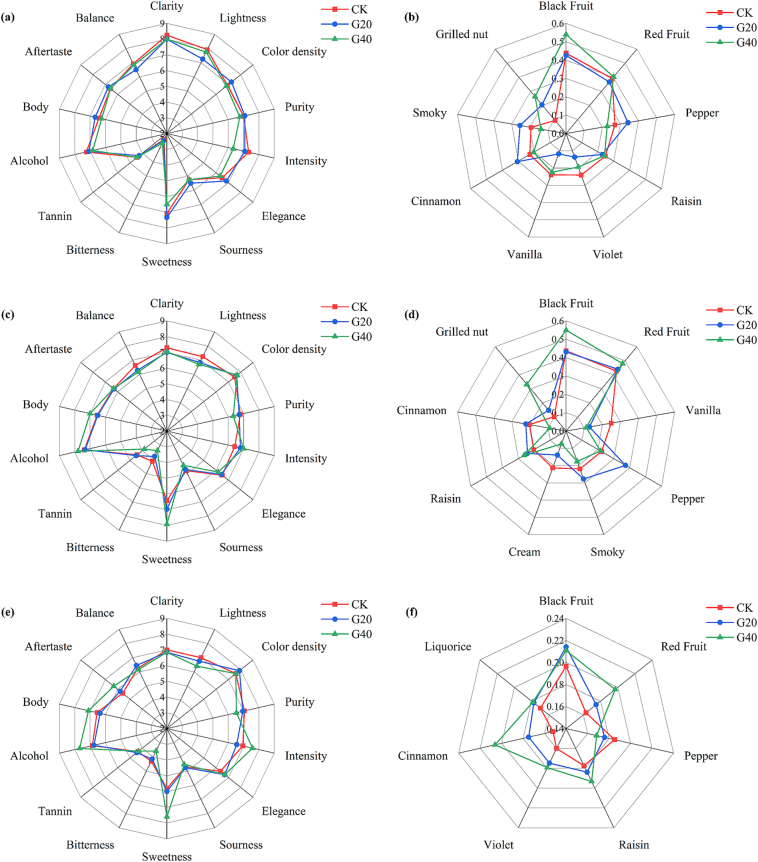
The sensory aroma profile of of Syrah fortified sweet wines made by fresh and dehydrated grapes at 0, 12, and 18 months of bottle aging. (a) Sensory quality before bottle aging; (b) M-value of aroma descriptors before bottle aging; (c) sensory quality at 12 months of bottle aging; (d) M-value of aroma descriptors at 12 months of bottle aging; (e) sensory quality at 18 months of bottle aging; (f) M-value of aroma descriptors at 18 months of bottle aging. Descriptors with ^⁎^ denote significant differences. Analyses of variance, levels of significance: ^⁎^(*p* < 0.05), ^⁎⁎^ (*p* < 0.01), ^⁎⁎⁎^(*p* < 0.001). Sample coding: CK, the control, made by 0 % weight loss grapes; G20, made by 20 % weight loss grapes; G40, made by 40 % weight loss grapes.

Fig. 5 shows a radar plot analysis of the sensory evaluation and aroma profile of Syrah fortified sweet wines. Similar to the previous analysis of the CIELab parameters, G20 and G40 wines scored higher on color and lower on lightness compared to CK wine, verifying that wine made from dehydrated grapes does have a more pleasing and deeper color. By comparison, the scores of lightness and color density decreased more slowly in G20 wines than in CK and G40 wines. This finding demonstrated that the differences in color characteristics due to different dehydration degrees accumulate with aging, and wines made from dehydrated grapes have better color features after aging. In terms of taste attributes, G40 was scored lower on sourness, whereas there was no significant discrepancy between G20 and CK, probably due to the fact that the higher sweetness of G40 had a masking effect on the acidity.

### 3.7 Correlation analysis and mantel test of volatile compounds

**Fig. 6:**
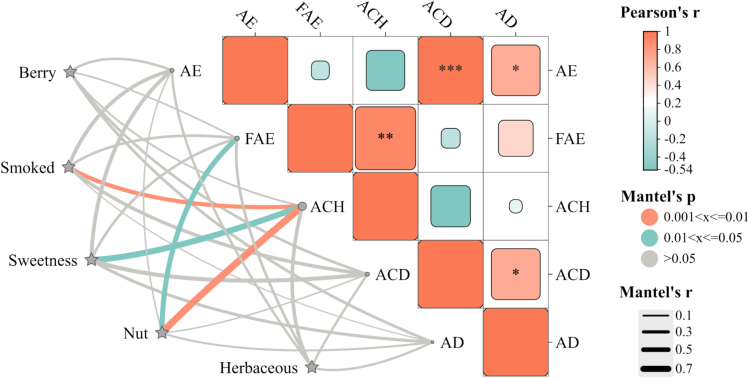
Correlation analysis of volatile compounds and Mantel test result of Syrah fortified sweet wines before bottle aging. * denote significant of correlation analysis, levels of significance: *(*p* < 0.05), ** (*p* < 0.01), ***(*p* < 0.001). Coding: AE, acetic esters; FAE, fatty acid ethyl esters; ACH, alcohols; ACD, acids; AD, aldehydes.

Perceptual interactions with other odor-active compounds may have an impact on the sensory impact of odor-active compounds on wine aroma (Ma et al., 2022). Therefore, the single odorant may have various aroma characteristics in different global aroma environments. The Correlation analysis and Mantel test were used to investigate the correlations between volatile compounds and aroma characteristics (Fig. 6). The volatile compound has a greater effect on the aroma profile when Mantel's r is higher and Mantel's p is lower. The correlation results further emphasizes that there was a highly significant positive correlation between the contents of acetic esters and acids (*p* < 0.001) and a significant positive correlation between the contents of alcohols and fatty acid ethyl esters (*p* < 0.01). The results of the Mantel analysis showed that the alcohols' content was positive significantly correlated with smoked (*p* < 0.01, *r* = 0.45), sweetness (*p* = 0.05, *r* = 0.70), and nutty (*p* < 0.01, *r* = 0.82) aromas. The content of fatty acid ethyl esters was positive significantly correlated with nutty (*p* < 0.05, *r* = 0.57). This suggests that alcohols and fatty acid ethyl esters had a positive contributing role to the smoky, sweet and nutty aromas in Syrah fortified sweet wines.

## 4 Conclusions

In summary, the preceding analysis indicate that the dehydration has an impact on every facet of Syrah fortified sweet wine, wines made from dehydrated grapes have more exceptional organoleptic properties and aging ability. Dehydration not only raises the reducing sugar and total acid content of fortified sweet wines but also enhances the stability of the phenolic compounds. Simultaneously, it promotes the concentration of organic acids and aroma substances, altering or improving the organoleptic properties and nutritive value of the wine. These effects are mainly due to the concentration effect and metabolic activities of dehydration. In addition, dehydration increases the color and aromatic qualities of the wine during aging, giving Syrah fortified sweet wine a greater capacity for aging. In this study, the G40 wines had better aromatic and color aging stability; however, darker color and better aromatic qualities were observed in the G20 wines. Specifically, the total phenolic content rose and then fell during dehydration, with G20 samples having the highest total phenolic content. And the monomeric anthocyanin content of G20 was less affected by oxidative degradation. Obviously, Syrah fortified sweet wines produced at middle and high dehydration levels showed the better aging abilities and organoleptic properties. Therefore, producing fortified sweet wines may be a fantastic option for Ningxia in China to improve the aging potential of wines and relieve the problems of color lightening, aroma dissipating and taste fading during storage.

## Ethical statement

The study was reviewed and approved by the Northwest A&F University and informed consent was obtained from each participant before their participation in the study. Participants gave informed consent via the statement “I am aware that my responses are confidential, and I agree to participate in this survey” where an affirmative reply was required to enter the survey. They were able to withdraw from the survey at any time without giving a reason. The wines tested were safe for consumption.

## Funding

This work was supported by the International Science and Technology Cooperation Project of Shaanxi Province [grant numbers No.2023-GHZD-39] and 10.13039/100006190Xinjiang High Quality Aged Wine Products Research and Development Program [grant numbers K4050722060].

## CRediT authorship contribution statement

**Xiaoxi Zhang:** Writing – original draft, Software, Methodology, Data curation. **Kaixian Wang:** Writing – original draft, Investigation, Data curation. **Qun Wang:** Writing – review & editing. **Zhenan Shi:** Writing – review & editing. **Hélder Oliveira:** Writing – review & editing, Supervision. **Nuno Mateus:** Writing – review & editing, Supervision. **Fuliang Han:** Writing – review & editing, Validation, Methodology, Funding acquisition, Conceptualization.

## Declaration of competing interest

The authors declare that they have no known competing financial interests or personal relationships that could have appeared to influence the work reported in this paper.

## Data Availability

Data will be made available on request.

## Appendix A Supplementary data

**Figure S1.** Record of grape dehydration phenomenon. Image 1

**Figure S2.** Chromatogram of CK-1 sample Image 2

**Figure S3.** Chromatogram of CK-2 sample Image 3

**Figure S4.** Chromatogram of G20-1 sample Image 4

**Figure S5.** Chromatogram of G20-2 sample Image 5

**Figure S6.** Chromatogram of G40-1 sample Image 6

**Figure S7.** Chromatogram of G40-2 sample Image 7

**Figure S8.** Mass Spectrometry of 1-Hexanol Image 8

**Figure S9.** Mass Spectrometry of 1-Octanol Image 9

**Figure S10.** Mass Spectrometry of 2-Ethylhexanol Image 10

**Figure S11.** Mass Spectrometry of 2-Methyl-1-propanol Image 11

**Figure S12.** Mass Spectrometry of 3-Methyl-1-butanol Image 12

**Figure S13.** Mass Spectrometry of 3-Methyl-1-pentanol Image 13

**Figure S14.** Mass Spectrometry of 3-Methylbutyl acetate Image 14

**Figure S15.** Mass Spectrometry of 3-Methylthio-1-propanol Image 15

**Figure S16.** Mass Spectrometry of Acetic acid Image 16

**Figure S17.** Mass Spectrometry of Acetoin Image 17

**Figure S18.** Mass Spectrometry of Dithyl succinate Image 18

**Figure S19.** Mass Spectrometry of Ethyl 2-methylbutanoate Image 19

**Figure S20.** Mass Spectrometry of Ethyl 2-methylpropanoate Image 20

**Figure S21.** Mass Spectrometry of Ethyl 3-methylbutanoate Image 21

**Figure S22.** Mass Spectrometry of Ethyl acetate Image 22

**Figure S23.** Mass Spectrometry of Ethyl butyrate Image 23

**Figure S24.** Mass Spectrometry of Ethyl decanoate Image 24

**Figure S25.** Mass Spectrometry of Ethyl dodecanoate Image 25

**Figure S26.** Mass Spectrometry of Ethyl hexadecanoate Image 26

**Figure S27.** Mass Spectrometry of Ethyl hexanoate Image 27

**Figure S28.** Mass Spectrometry of Ethyl lactate Image 28

**Figure S29.** Mass Spectrometry of Ethyl octanoate Image 29

**Figure S30.** Mass Spectrometry of Furfural Image 30

**Figure S31.** Mass Spectrometry of Isobutyric acid Image 31

**Figure S32.** Mass Spectrometry of Isovaleric acid Image 32

**Figure S33.** Mass Spectrometry of Methyl octanoate Image 33

**Figure S34.** Mass Spectrometry of Phenethyl acetate Image 34

**Figure S35.** Mass Spectrometry of Phenylethyl alcohol Image 35

**Figure S36.** Mass Spectrometry of α-Terpineol Image 36
