# Supplementary material for: Effect of dehydration of Syrah grape berries on the aging potential of fortified sweet wines in Ningxia of China
Source: Food Chem X. 2025 Jan 17;26:102197. doi: 10.1016/j.fochx.2025.102197 (PMC11851203; doi:10.1016/j.fochx.2025.102197)
Supplement: Supplementary file 1 — Figure S1. Record of grape dehydration phenomenon. [file mmc1.docx]

**
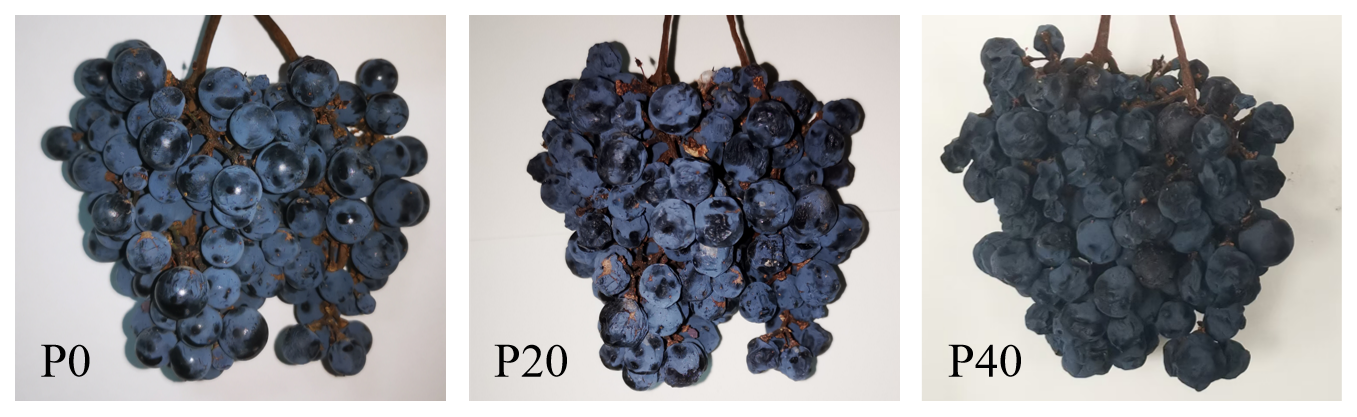
**

**Figure S1.** Record of grape dehydration phenomenon. P0, 0% weight loss grapes; P20, 20% weight loss grapes; P40, 40% weight loss grapes.
