# Supplementary figures and images for: Effect of dehydration of Syrah grape berries on the aging potential of fortified sweet wines in Ningxia of China
Source: Food Chem X. 2025 Jan 17;26:102197. doi: 10.1016/j.fochx.2025.102197 (PMC11851203; doi:10.1016/j.fochx.2025.102197)

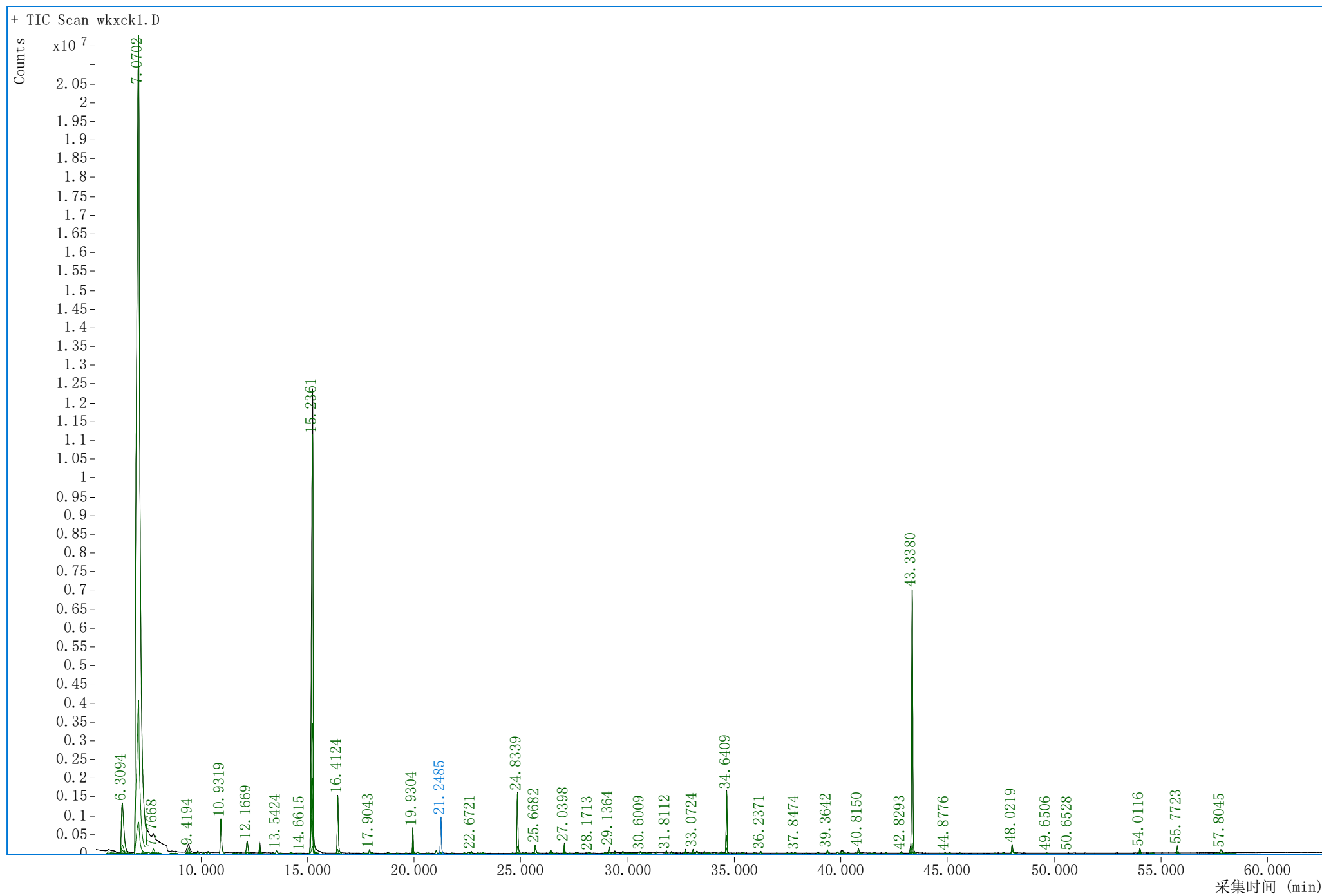

Supplement: Supplementary file 2 — Figure S2. Chromatogram of CK-1 sample [file mmc2.pdf]

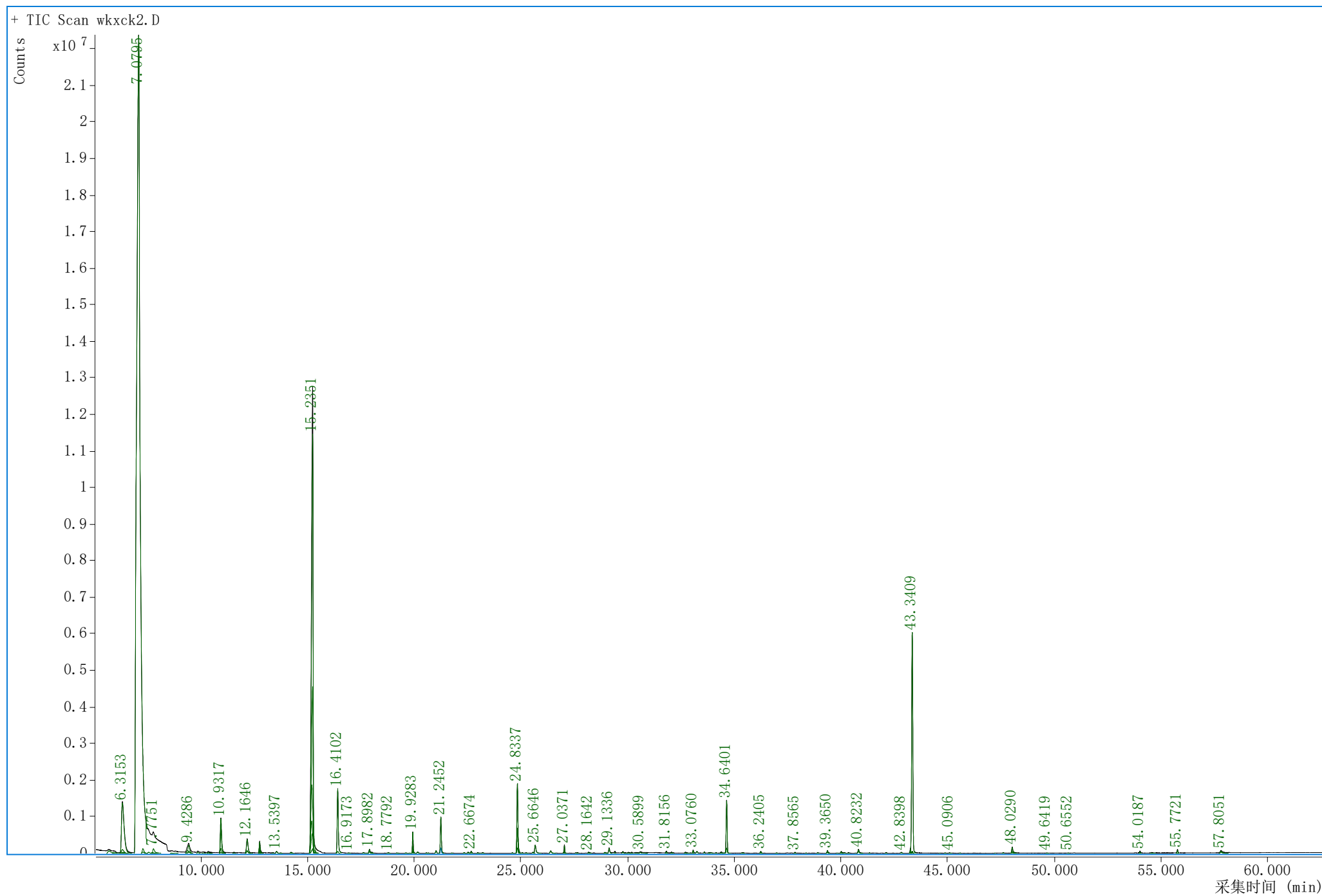

Supplement: Supplementary file 3 — Figure S3. Chromatogram of CK-2 sample [file mmc3.pdf]

+ TIC Scan wkxg21.D

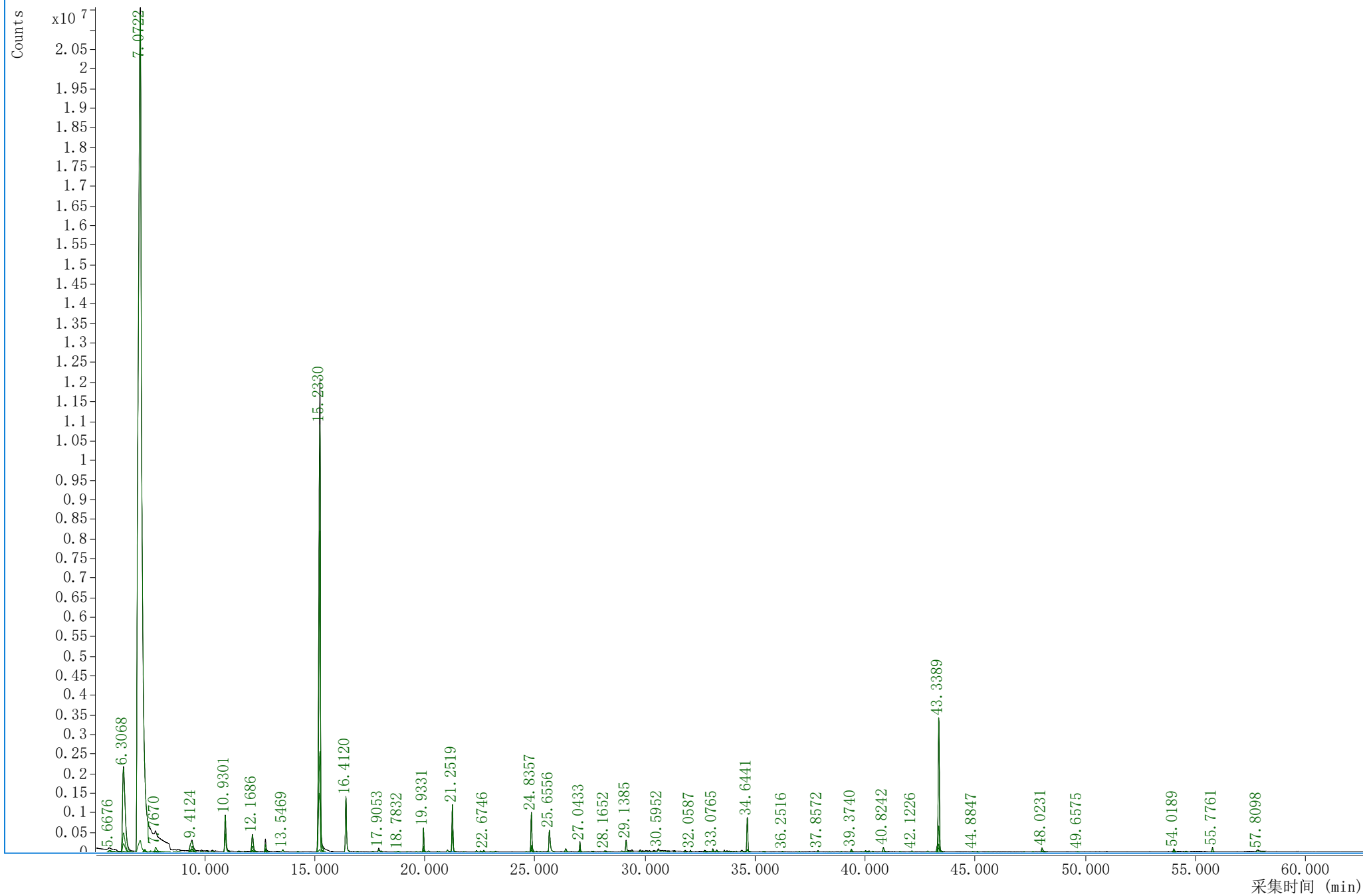

Supplement: Supplementary file 4 — Figure S4. Chromatogram of G20-1 sample [file mmc4.pdf]

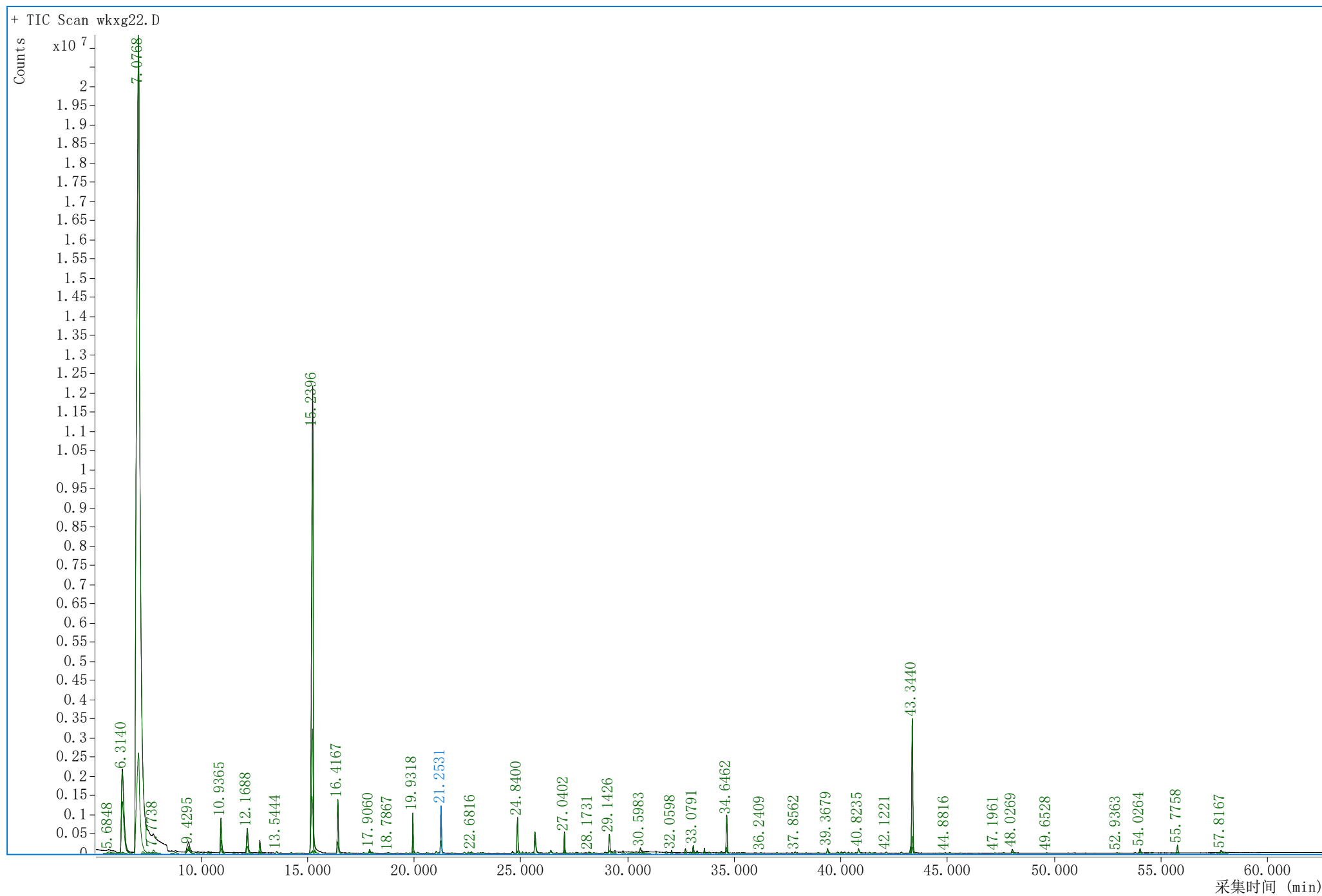

Supplement: Supplementary file 5 — Figure S5. Chromatogram of G20-2 sample [file mmc5.pdf]

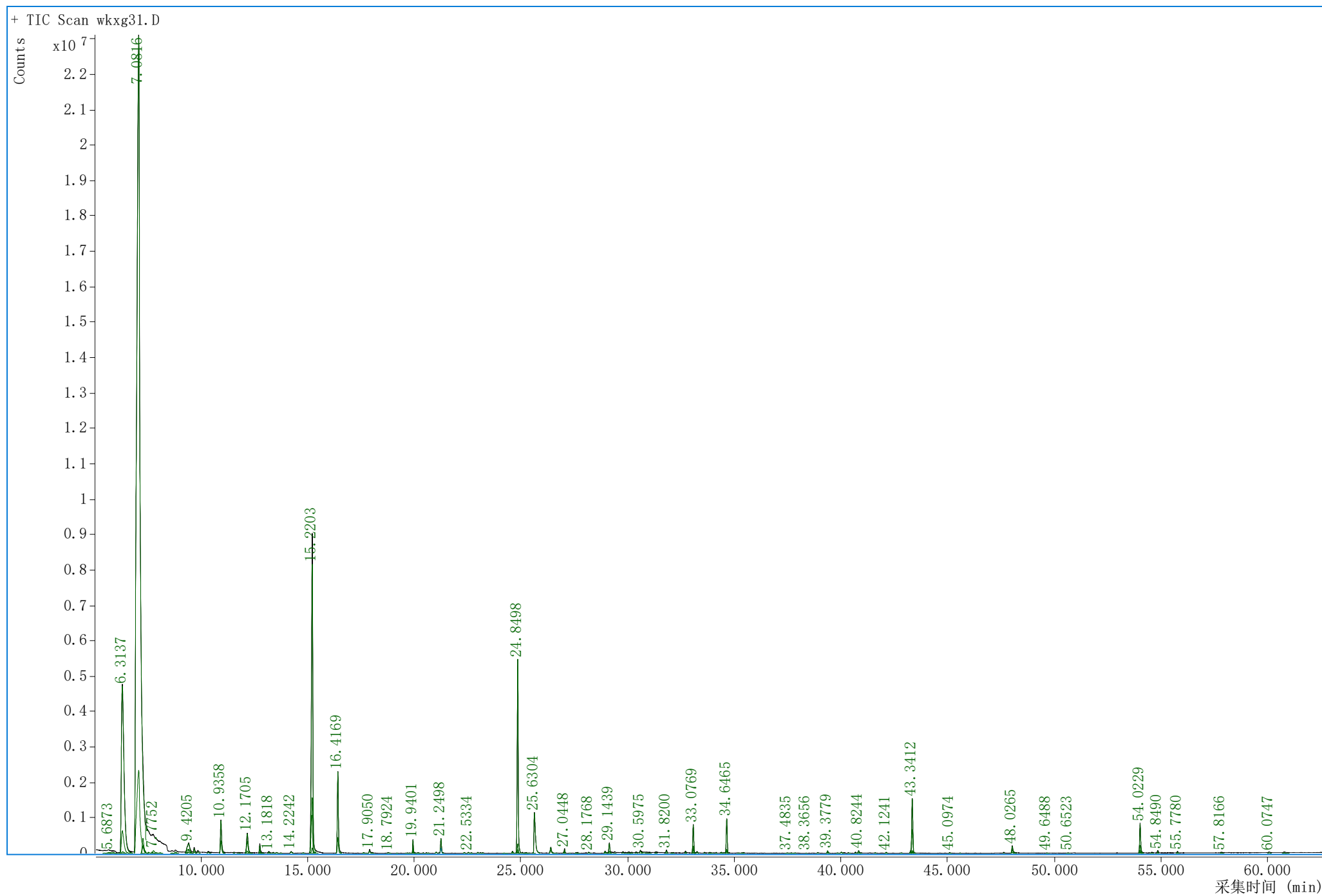

Supplement: Supplementary file 6 — Figure S6. Chromatogram of G40-1 sample [file mmc6.pdf]

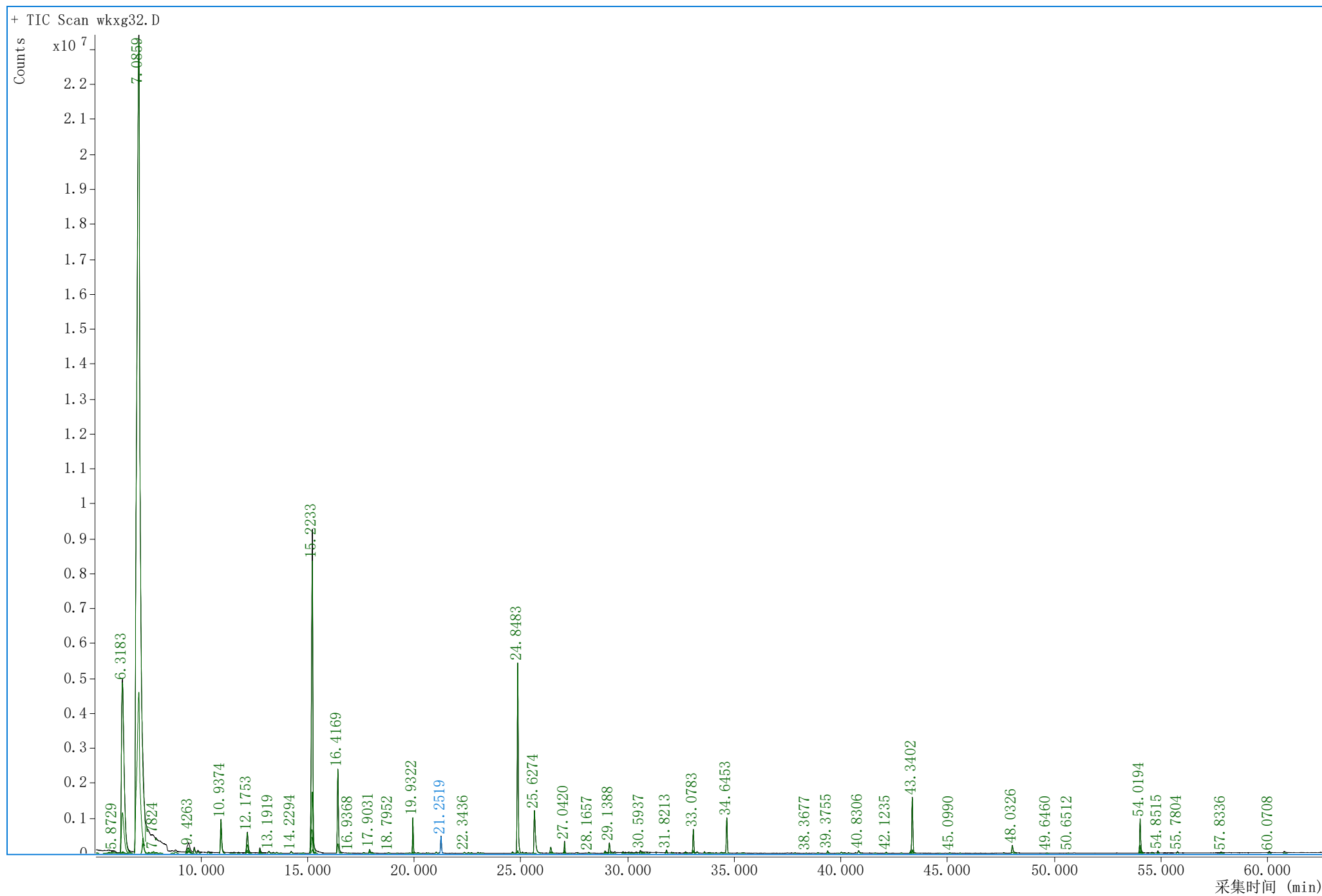

Supplement: Supplementary file 7 — Figure S7. Chromatogram of G40-2 sample [file mmc7.pdf]

组分 RT: 21.2443

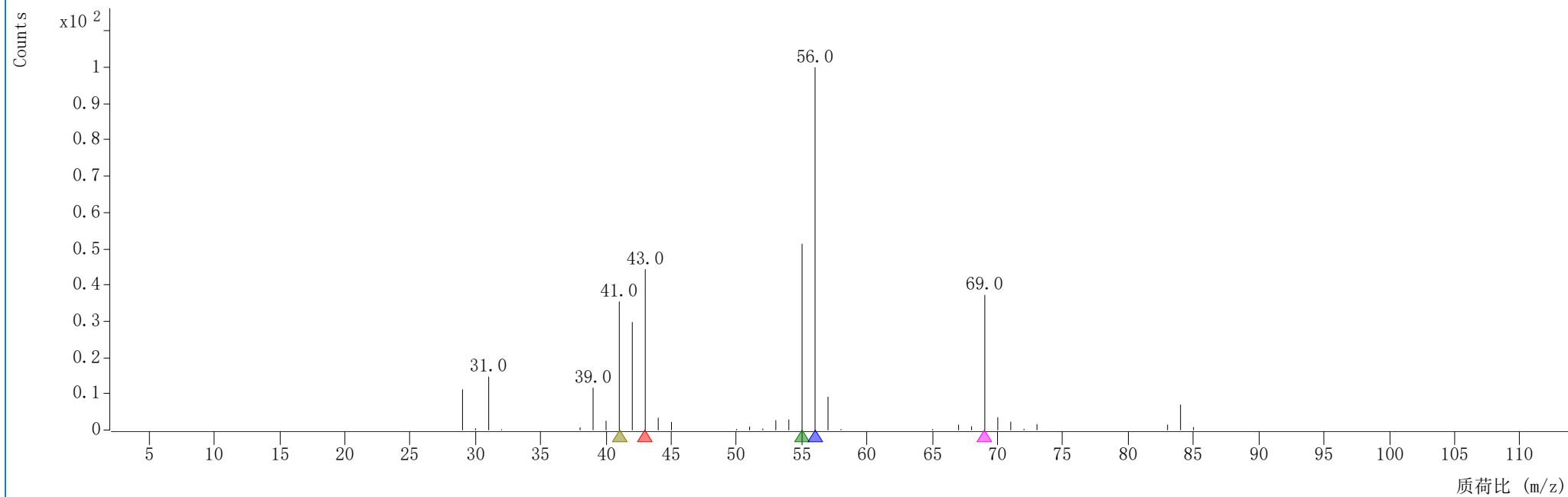

1-Hexanol (NIST17.L)

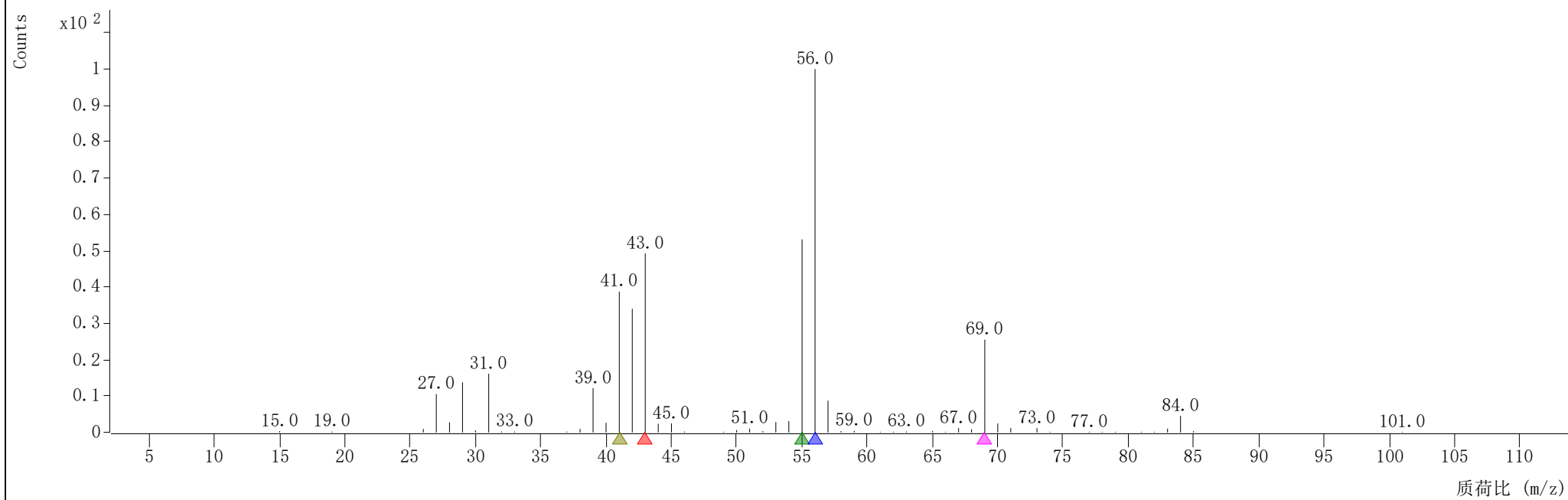

Supplement: Supplementary file 8 — Figure S8. Mass Spectrometry of 1-Hexanol [file mmc8.pdf]

组分 RT: 29.7798

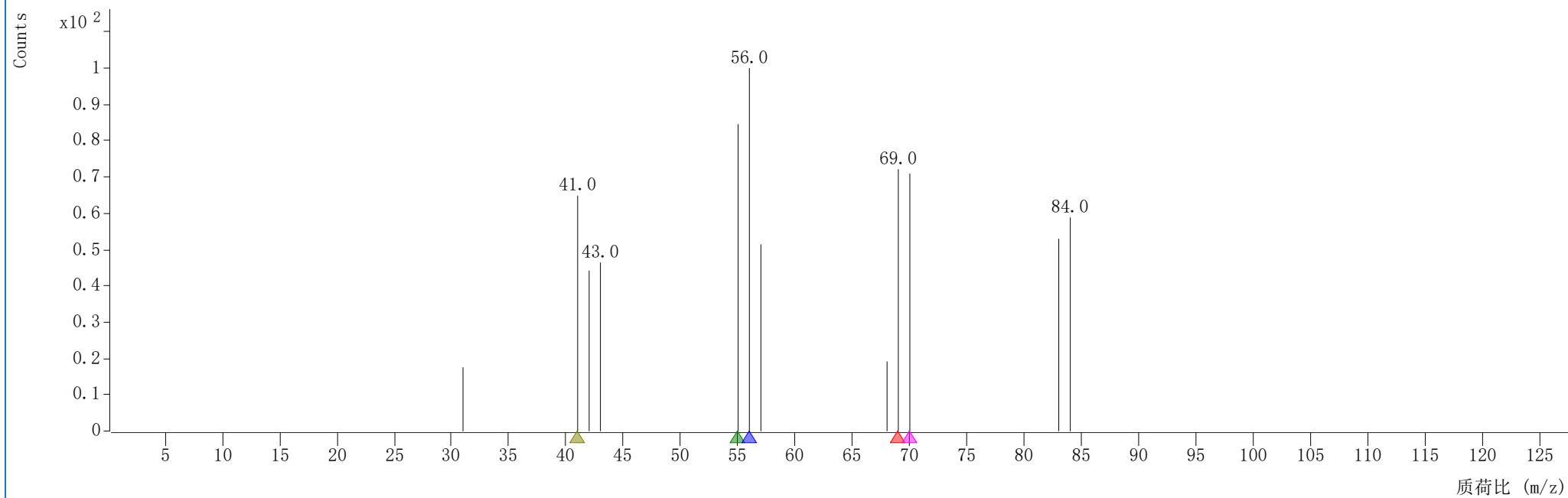

1-Octanol (NIST17.L)

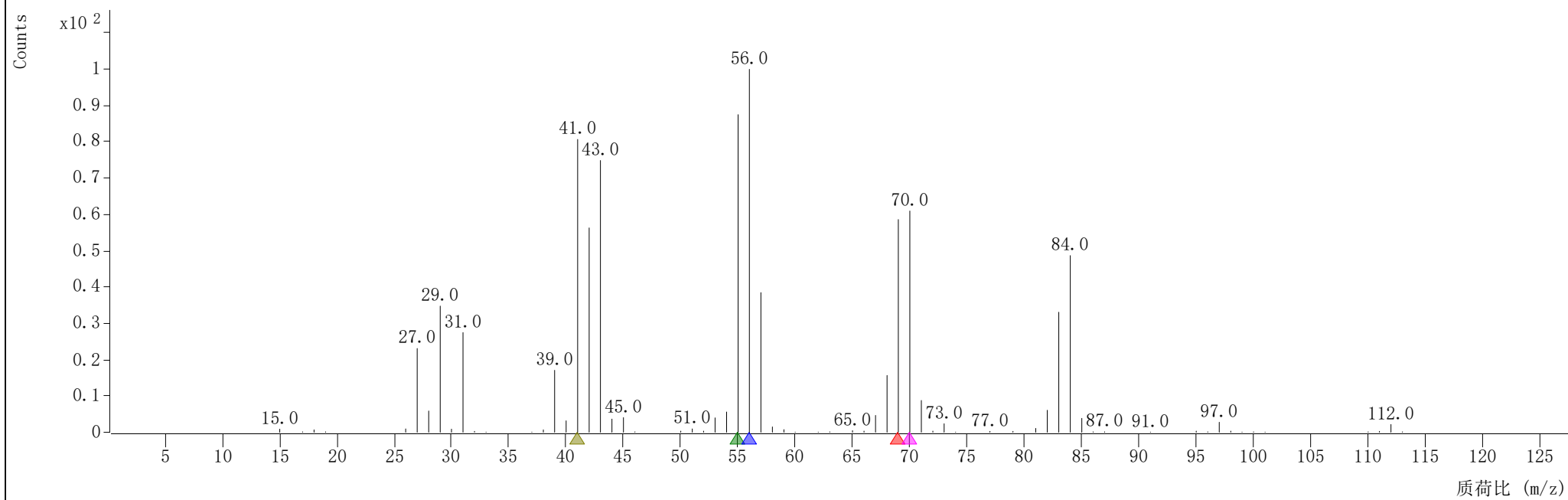

Supplement: Supplementary file 9 — Figure S9. Mass Spectrometry of 1-Octanol [file mmc9.pdf]

组分 RT: 27.0175

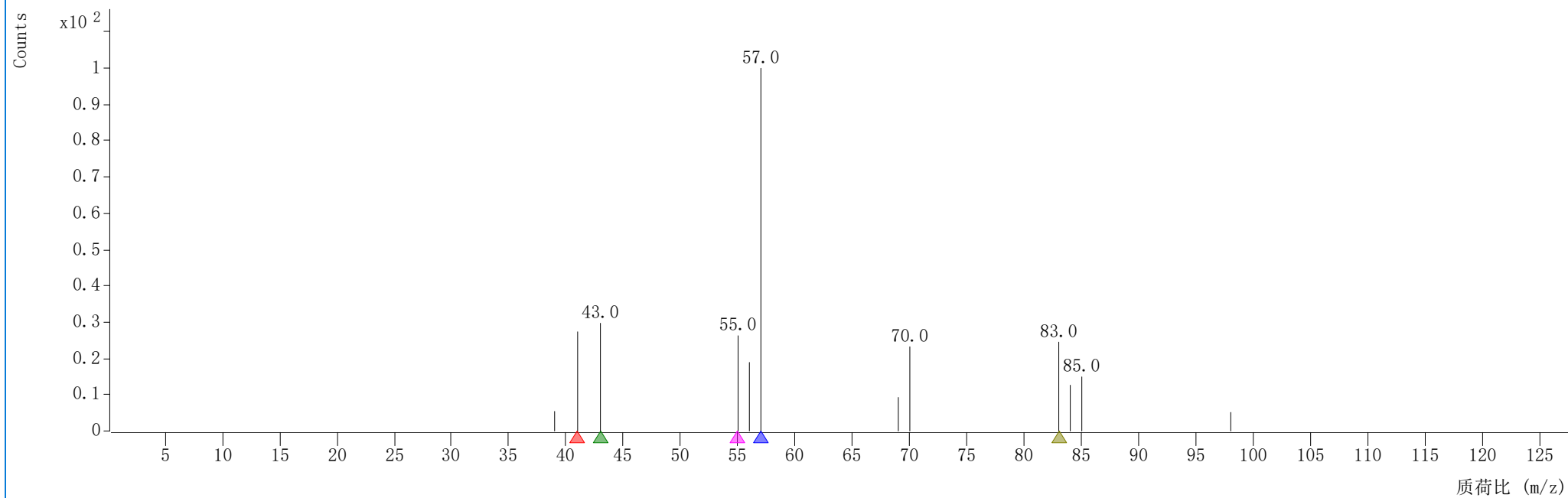

1-Hexanol, 2-ethyl- (NIST17.L)

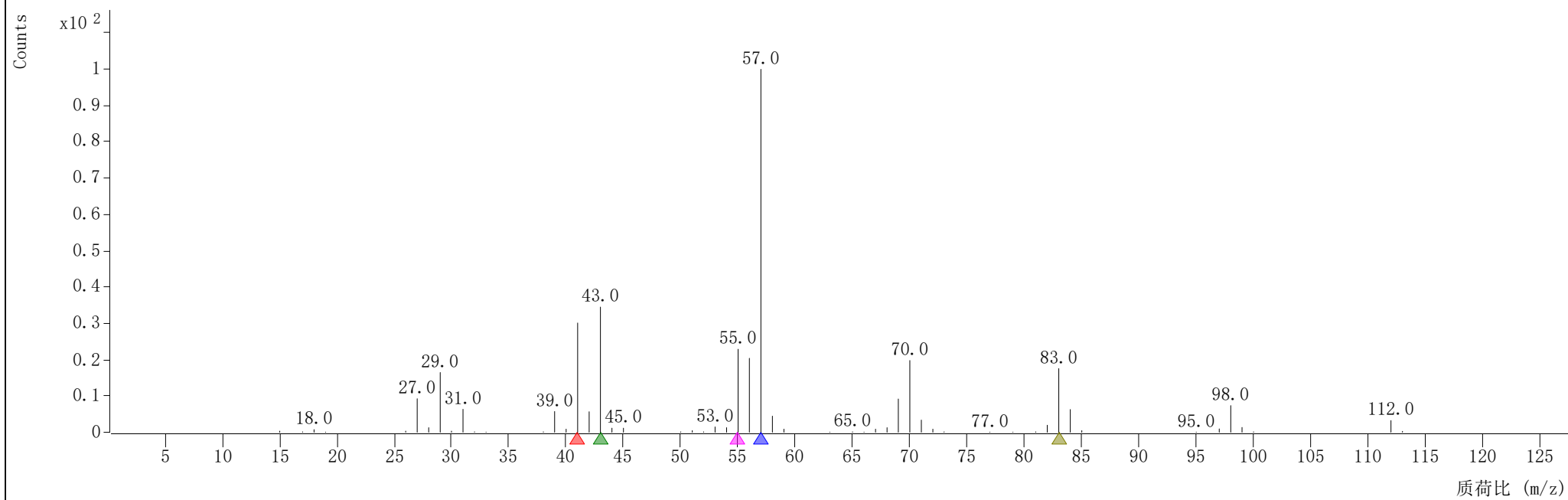

Supplement: Supplementary file 10 — Figure S10. Mass Spectrometry of 2-Ethylhexanol [file mmc10.pdf]

组分 RT: 10.9319

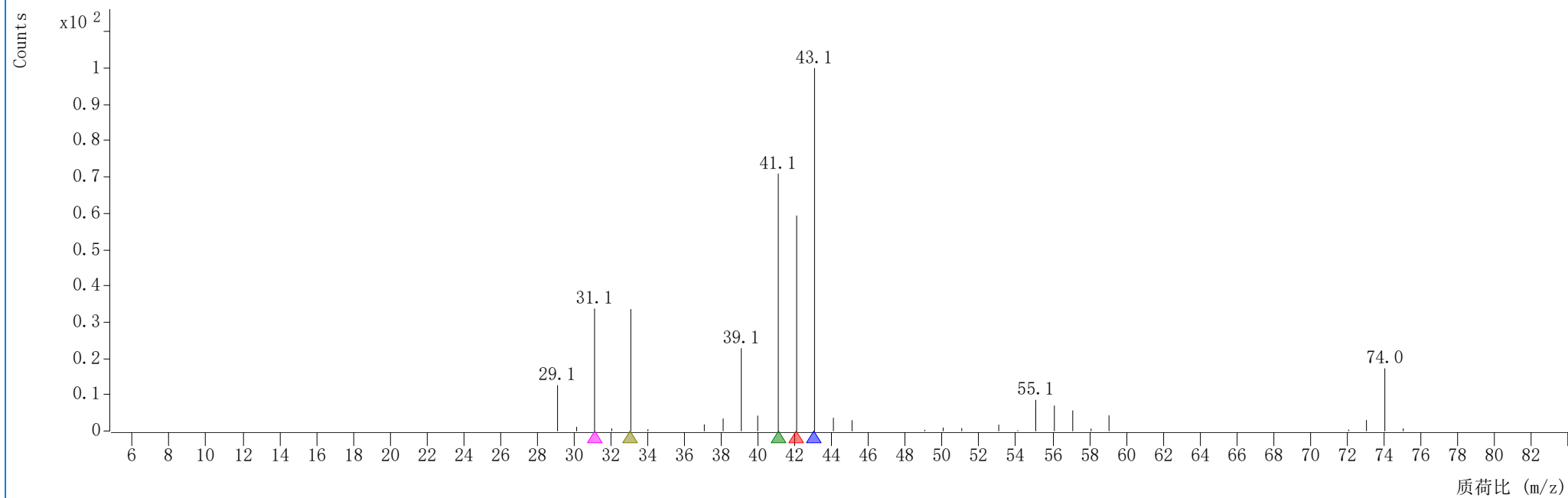

1-Propanol, 2-methyl- (NIST17.L)

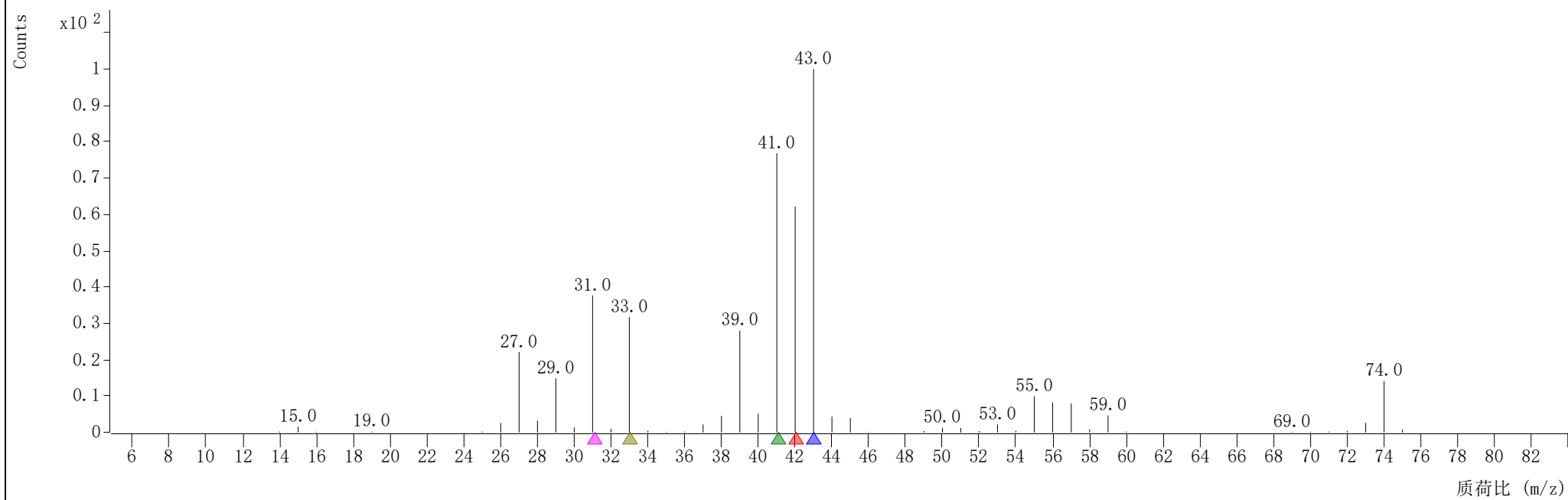

Supplement: Supplementary file 11 — Figure S11. Mass Spectrometry of 2-Methyl-1-propanol [file mmc11.pdf]

组分 RT: 15.2361

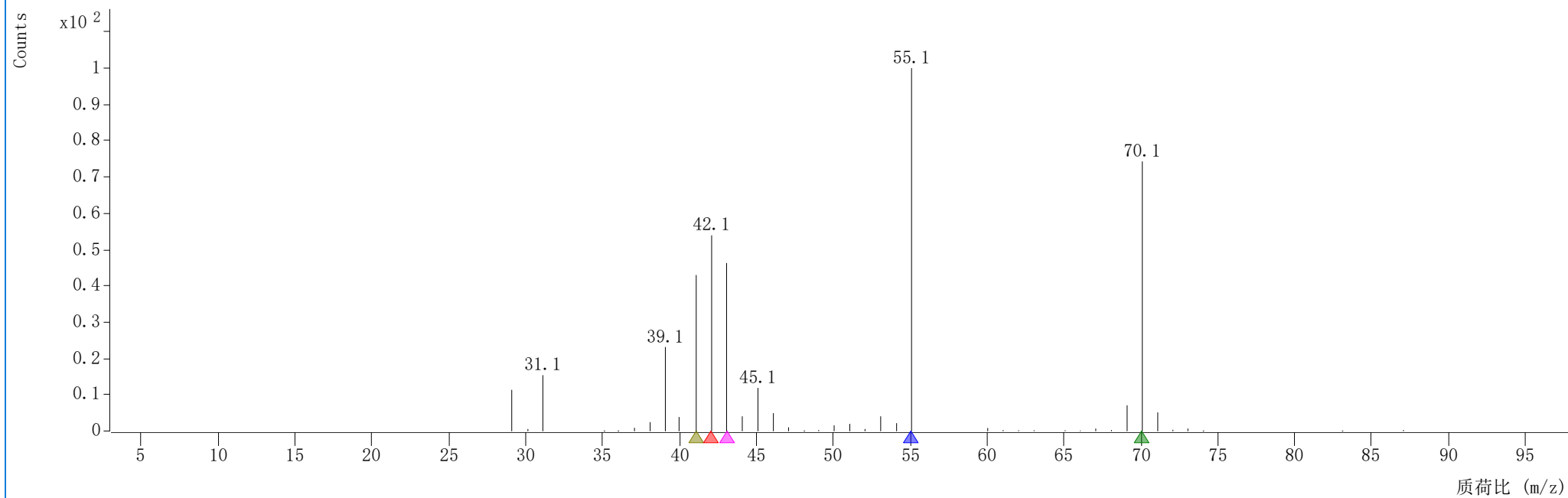

1-Butanol, 3-methyl- (NIST17.L)

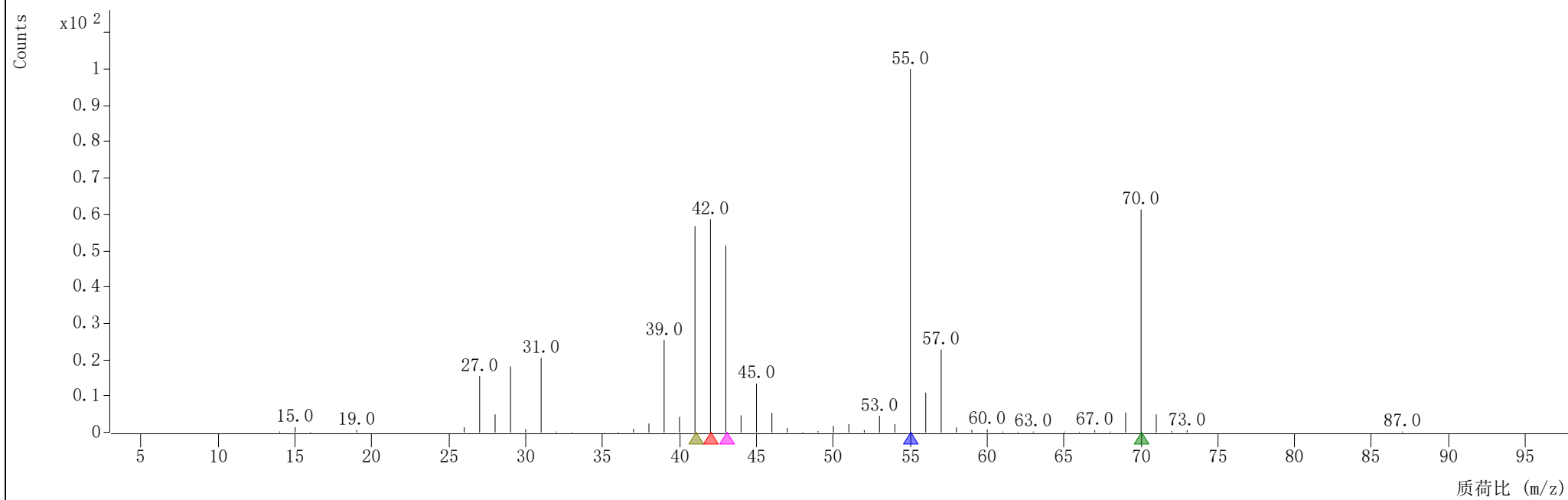

Supplement: Supplementary file 12 — Figure S12. Mass Spectrometry of 3-Methyl-1-butanol [file mmc12.pdf]

组分 RT: 20.1616

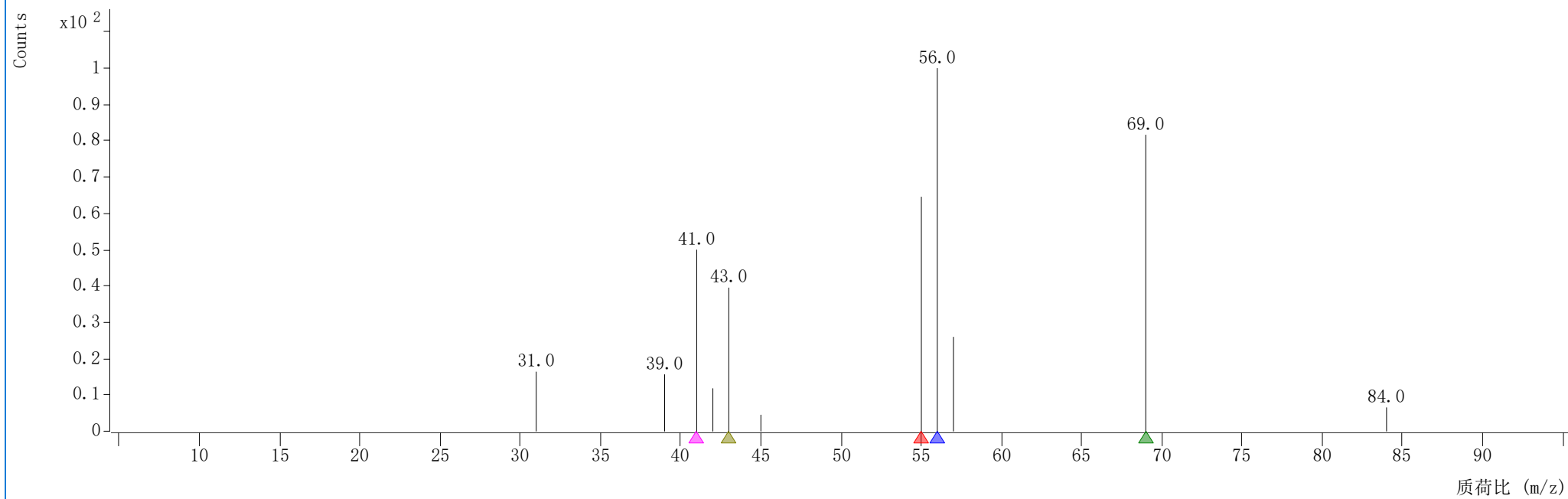

1-Pentanol, 3-methyl- (NIST17.L)

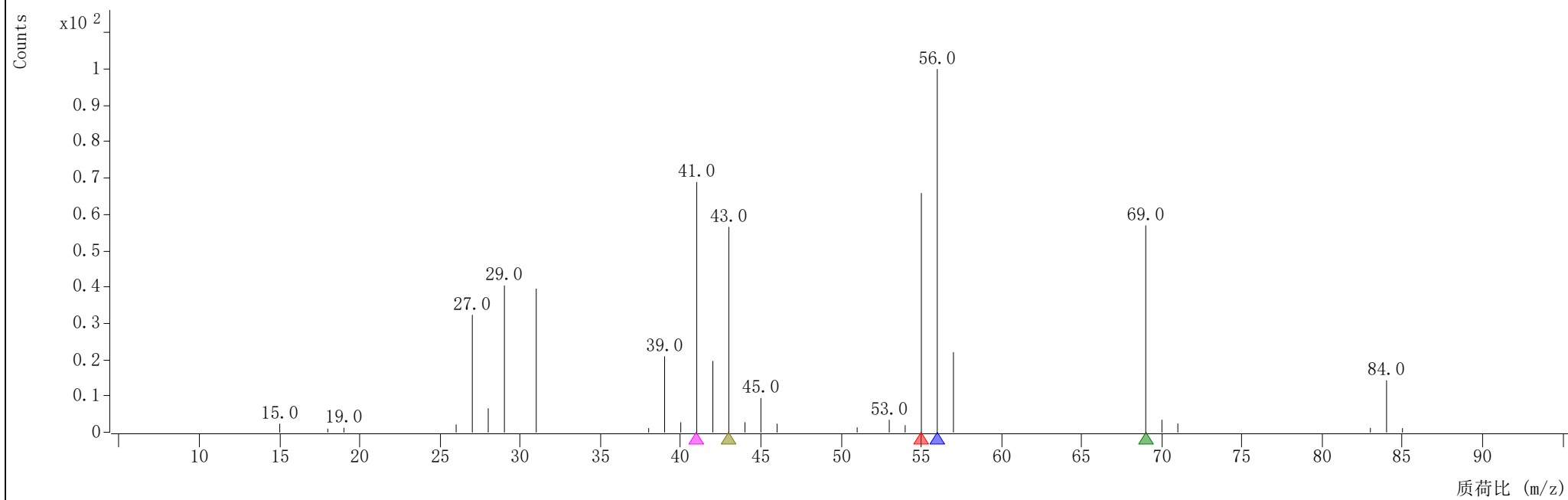

Supplement: Supplementary file 13 — Figure S13. Mass Spectrometry of 3-Methyl-1-pentanol [file mmc13.pdf]

组分 RT: 12.1569

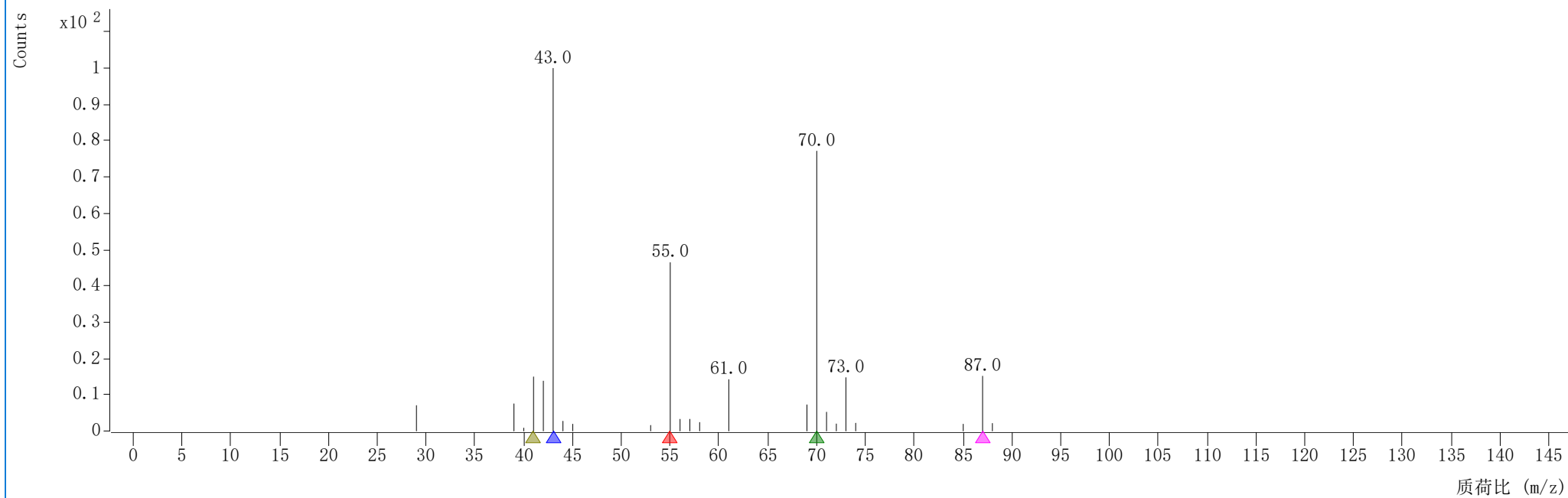

1-Butanol, 3-methyl-, acetate (NIST17.L)

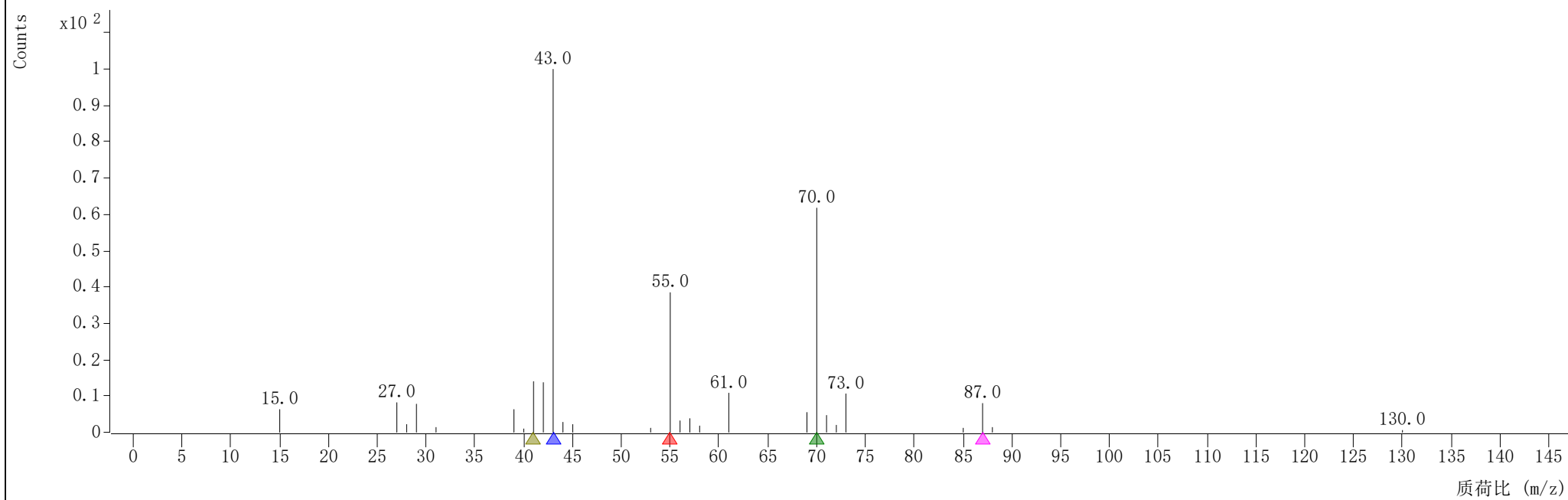

Supplement: Supplementary file 14 — Figure S14. Mass Spectrometry of 3-Methylbutyl acetate [file mmc14.pdf]

组分 RT: 36.2462

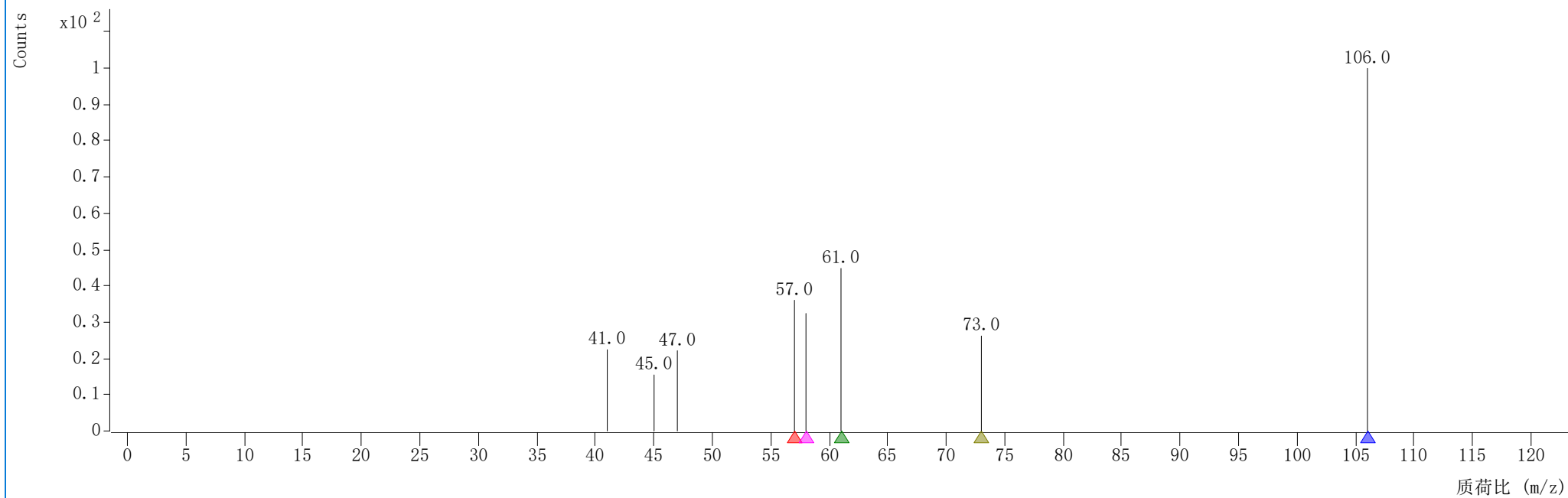

1-Propanol, 3-(methylthio)- (NIST17.L)

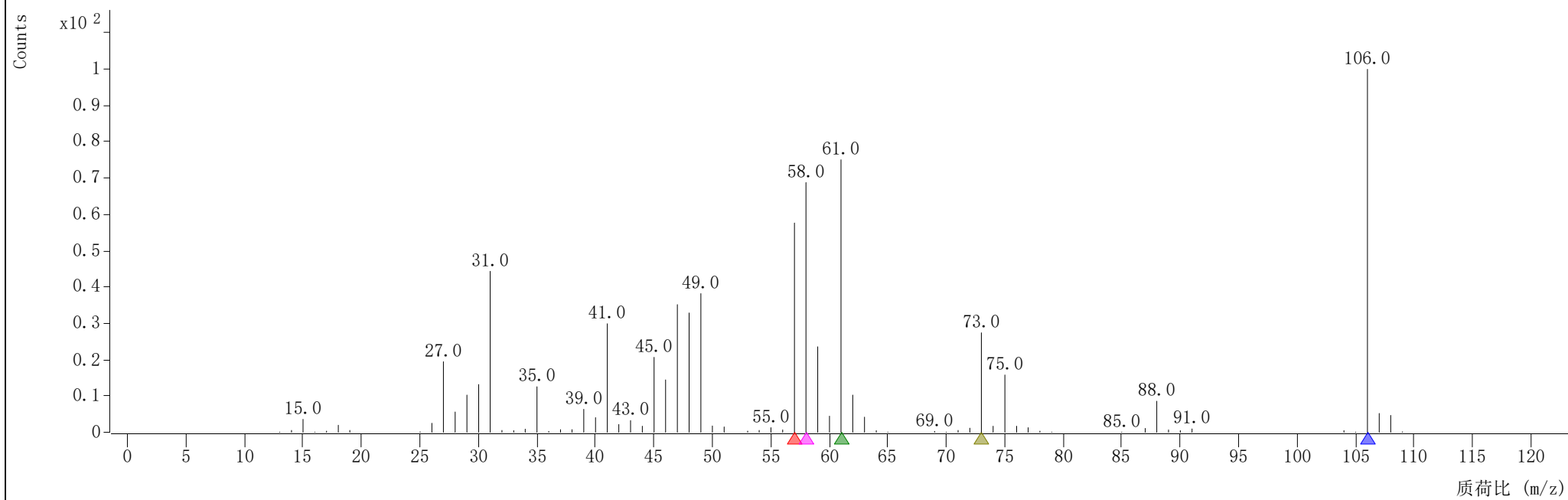

Supplement: Supplementary file 15 — Figure S15. Mass Spectrometry of 3-Methylthio-1-propanol [file mmc15.pdf]

组分 RT: 25.6556

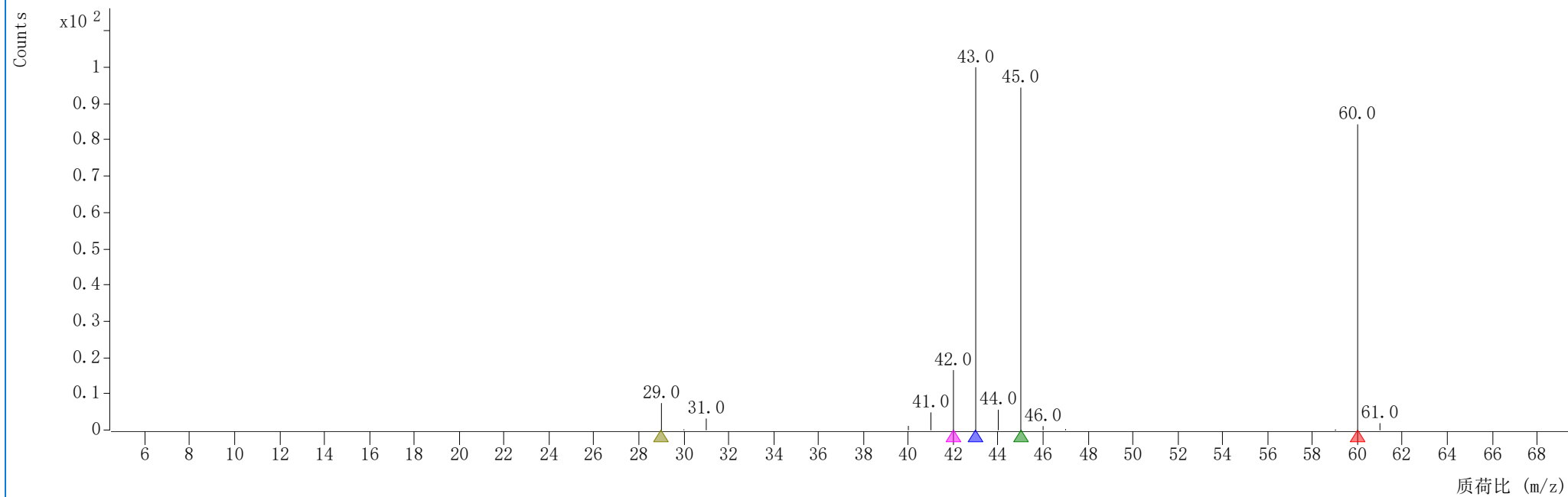

Acetic acid (NIST17.L)

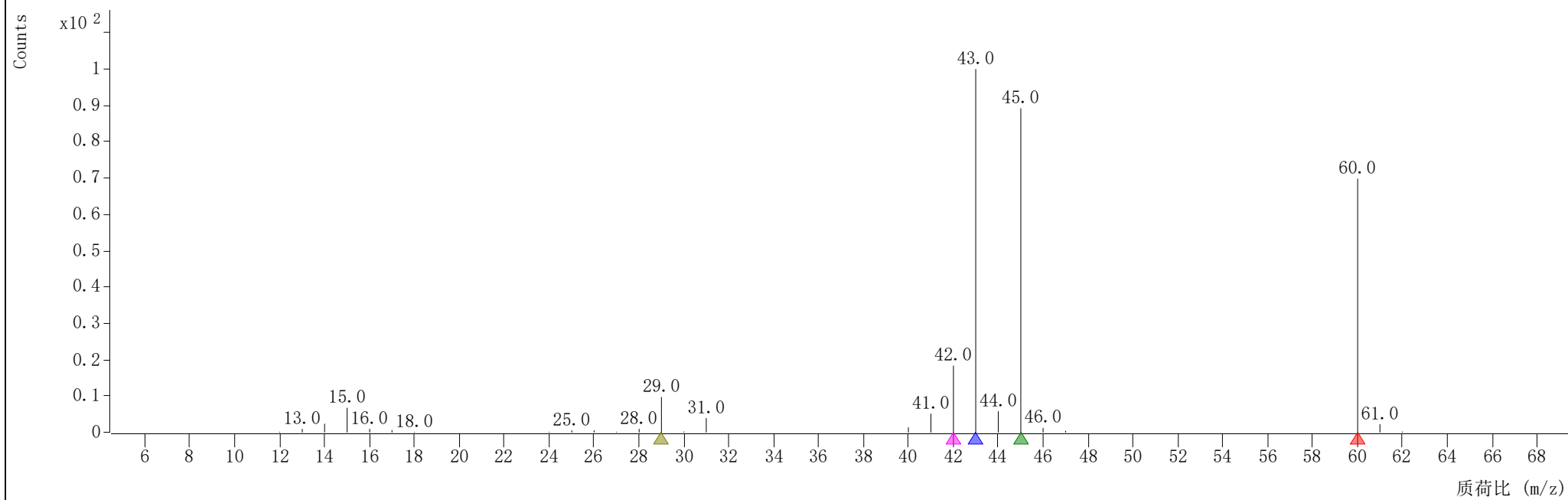

Supplement: Supplementary file 16 — Figure S16. Mass Spectrometry of Acetic acid [file mmc16.pdf]

组分 RT: 18.7890

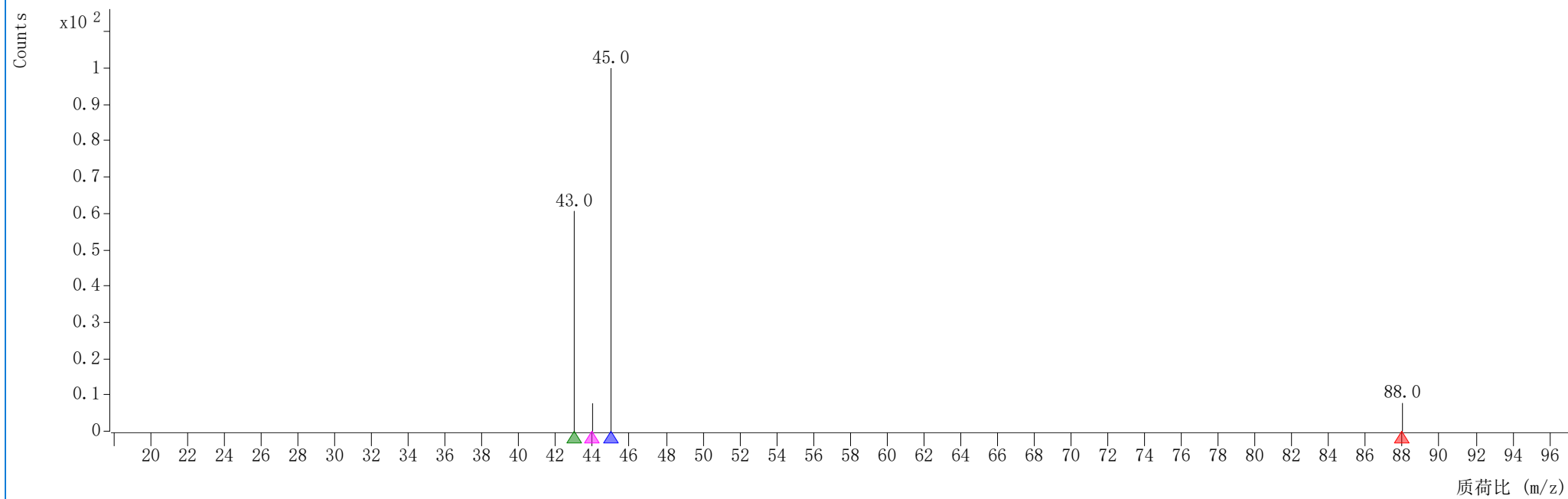

Acetoin (NIST17.L)

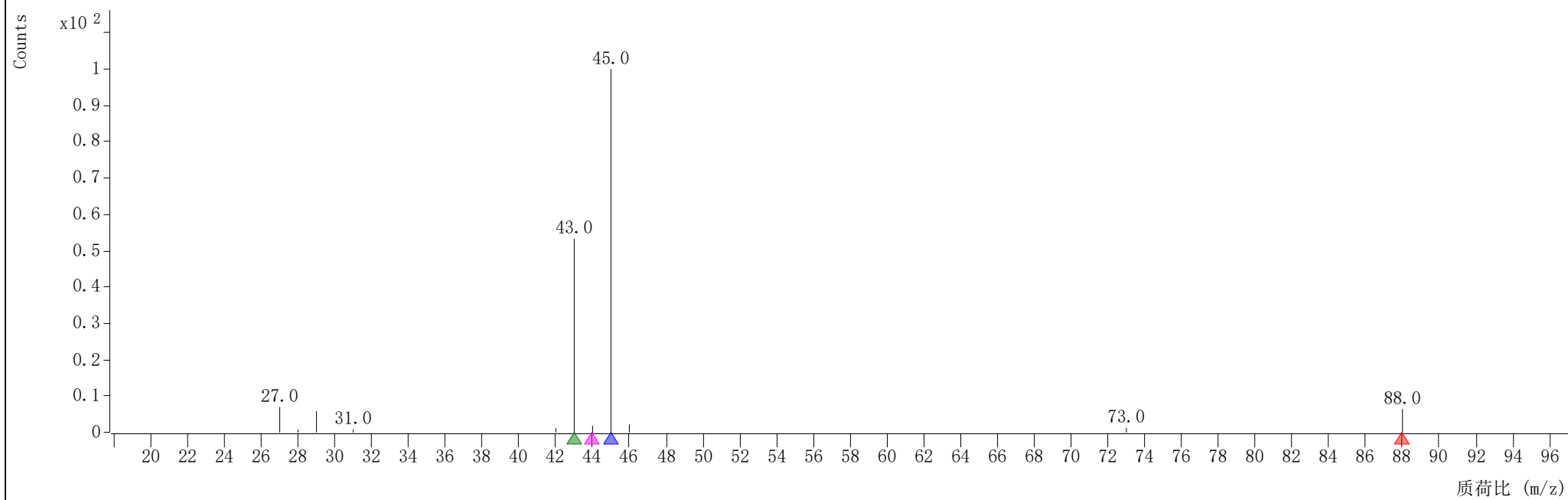

Supplement: Supplementary file 17 — Figure S17. Mass Spectrometry of Acetoin [file mmc17.pdf]

组分 RT: 34.6401

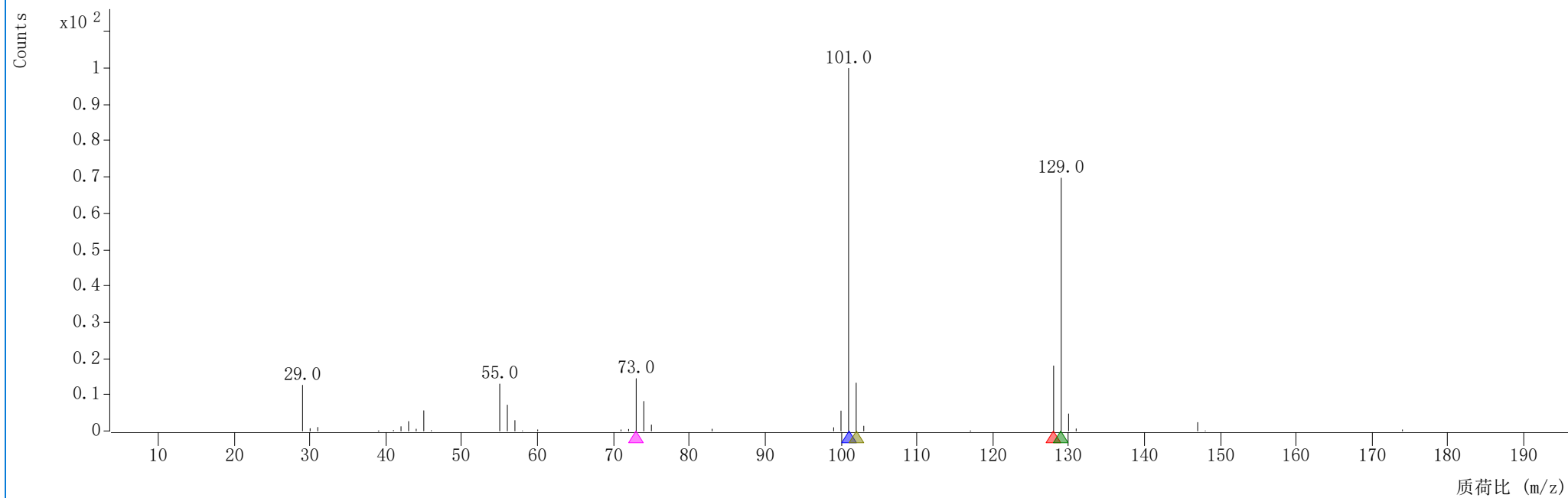

Butanedioic acid, diethyl ester (NIST17.L)

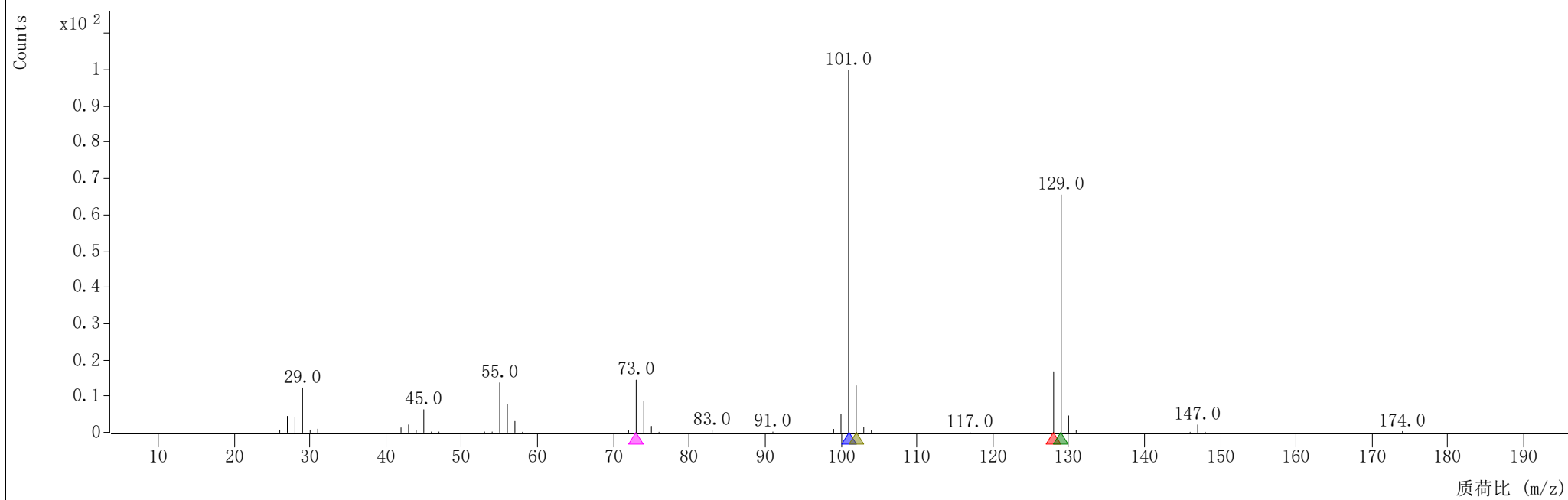

Supplement: Supplementary file 18 — Figure S18. Mass Spectrometry of Dithyl succinate [file mmc18.pdf]

组分 RT: 9.8516

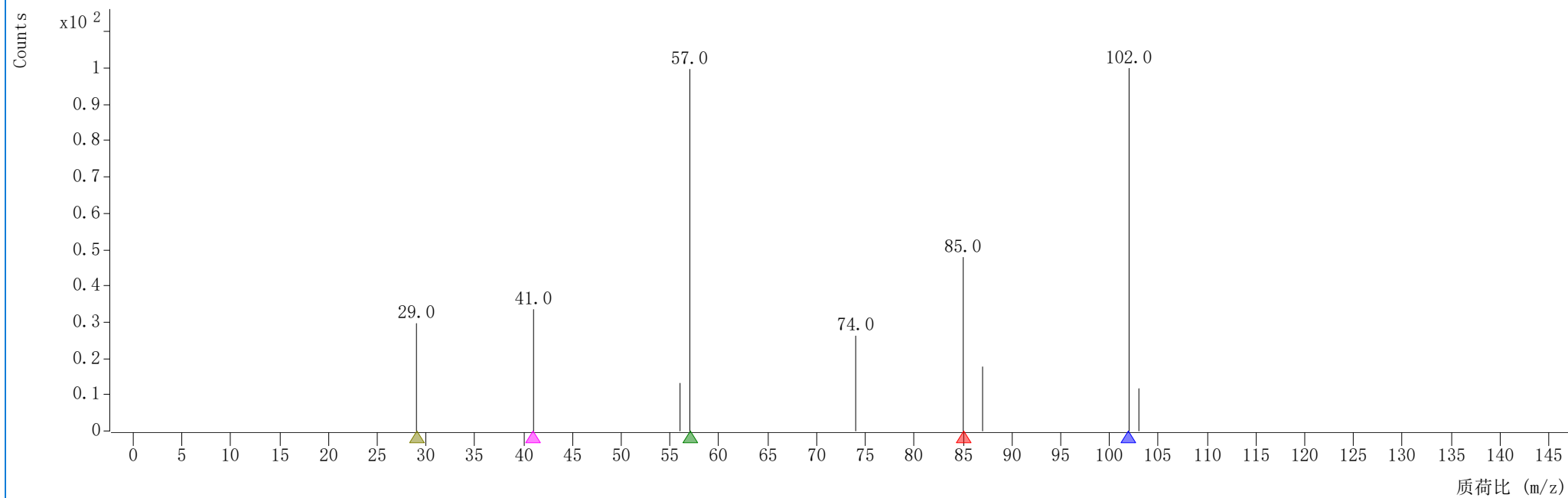

Butanoic acid, 2-methyl-, ethyl ester (NIST17.L)

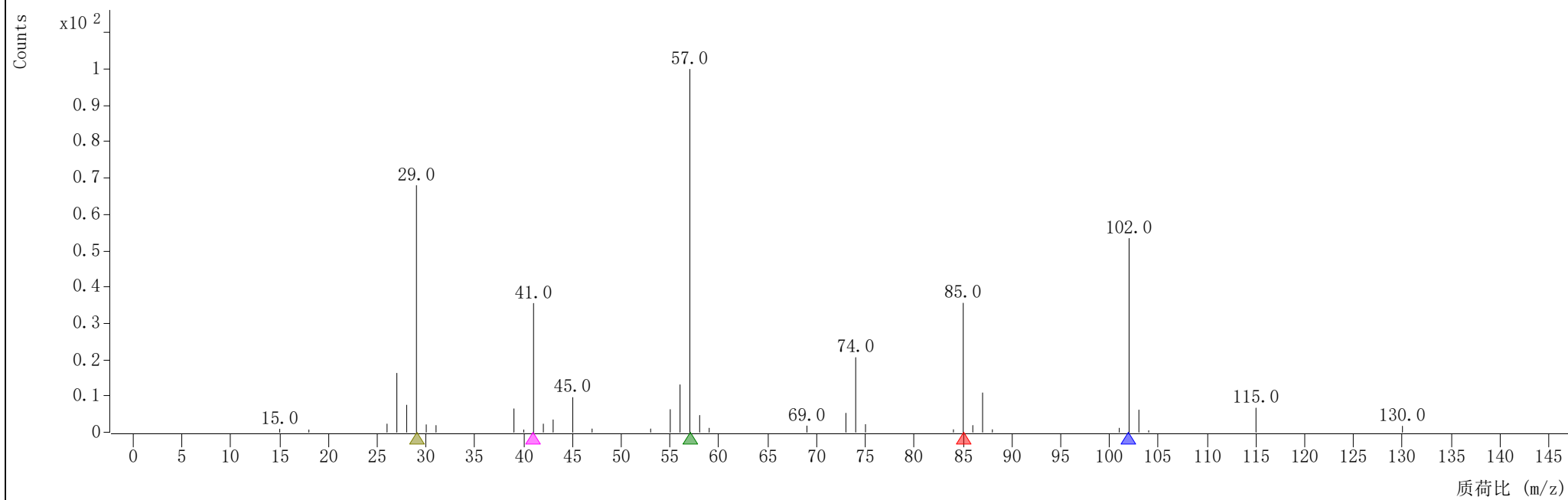

Supplement: Supplementary file 19 — Figure S19. Mass Spectrometry of Ethyl 2-methylbutanoate [file mmc19.pdf]

组分 RT: 7.6996

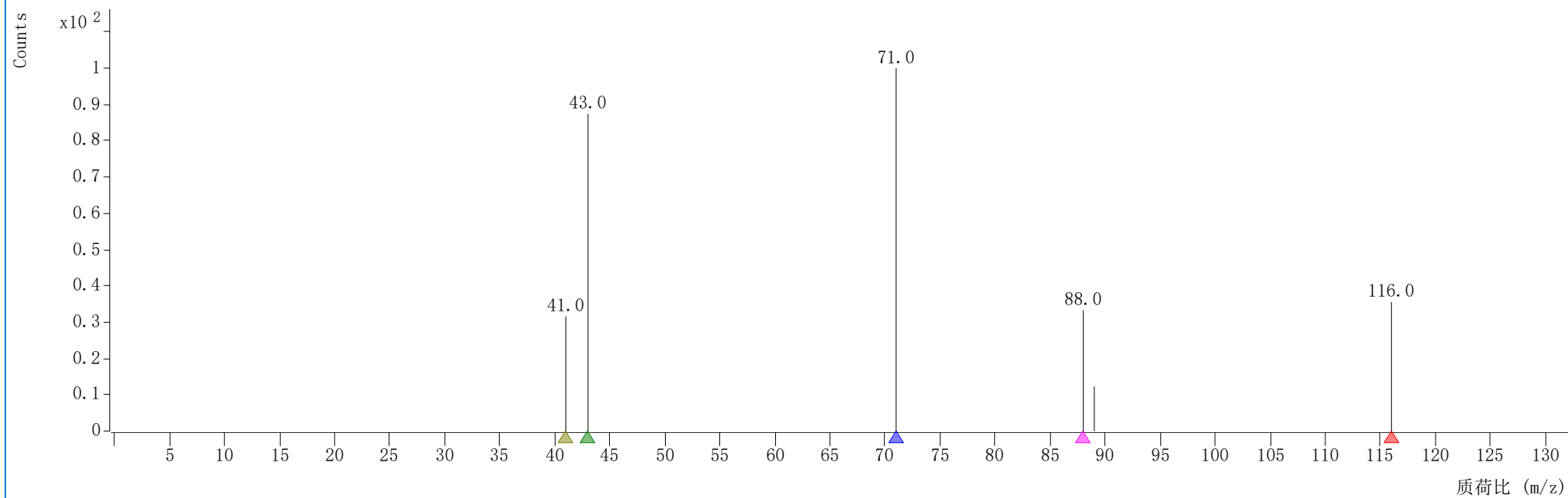

Propanoic acid, 2-methyl-, ethyl ester (NIST17.L)

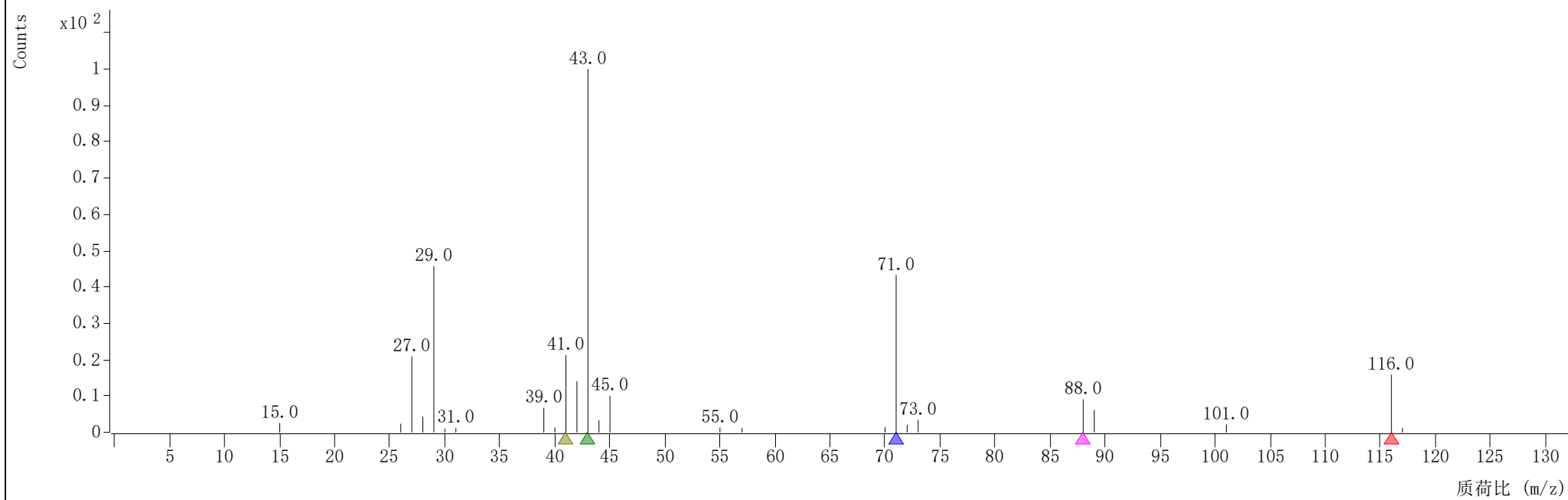

Supplement: Supplementary file 20 — Figure S20. Mass Spectrometry of Ethyl 2-methylpropanoate [file mmc20.pdf]

组分 RT: 10.3428

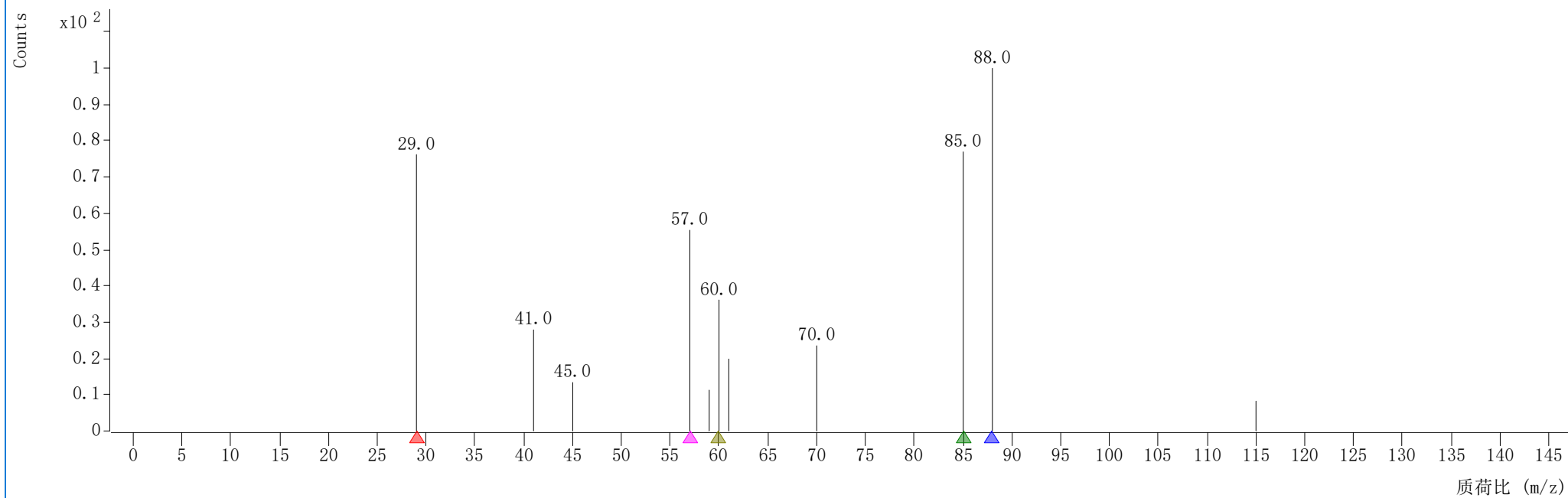

Butanoic acid, 3-methyl-, ethyl ester (NIST17.L)

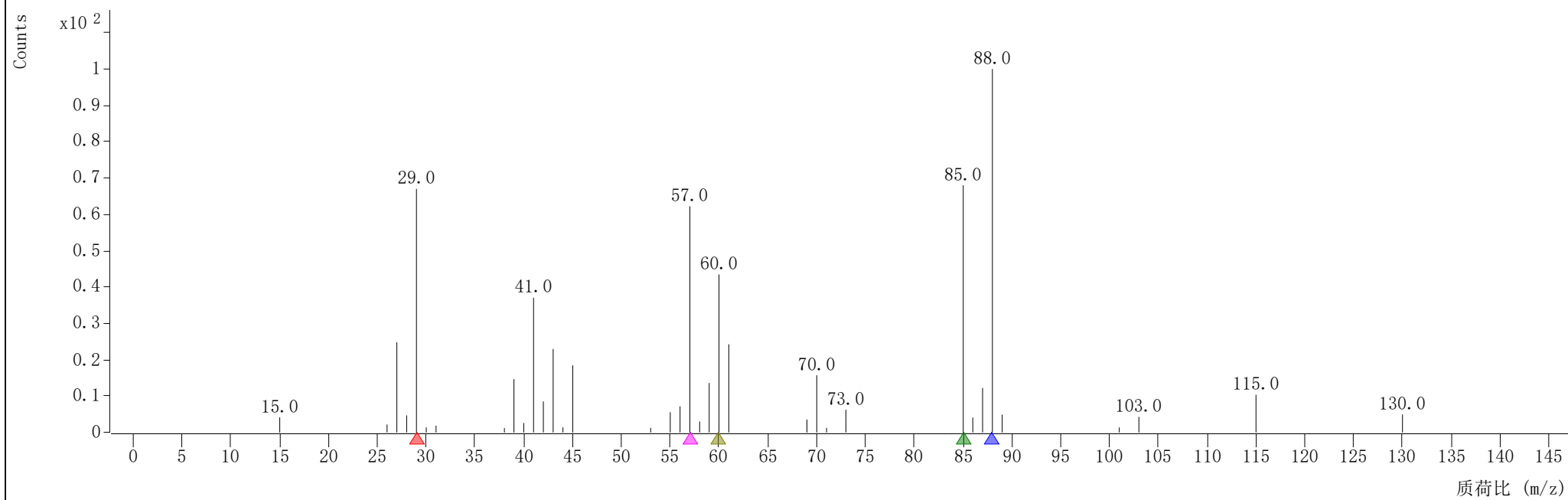

Supplement: Supplementary file 21 — Figure S21. Mass Spectrometry of Ethyl 3-methylbutanoate [file mmc21.pdf]

组分 RT: 6.3051

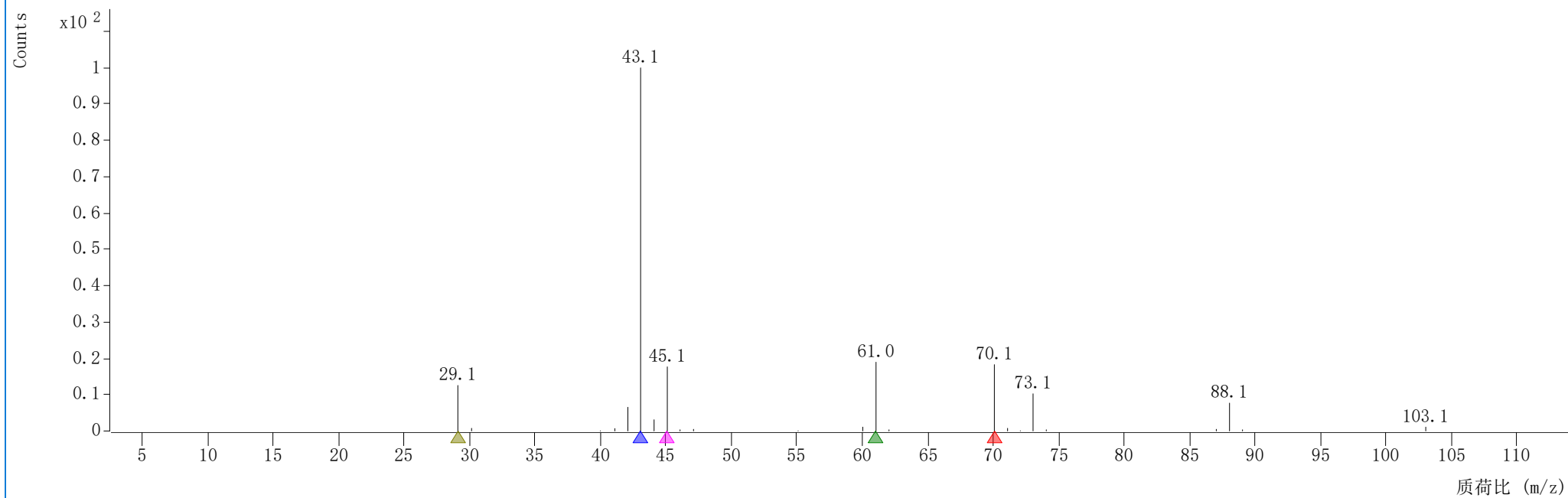

Ethyl Acetate (NIST17.L)

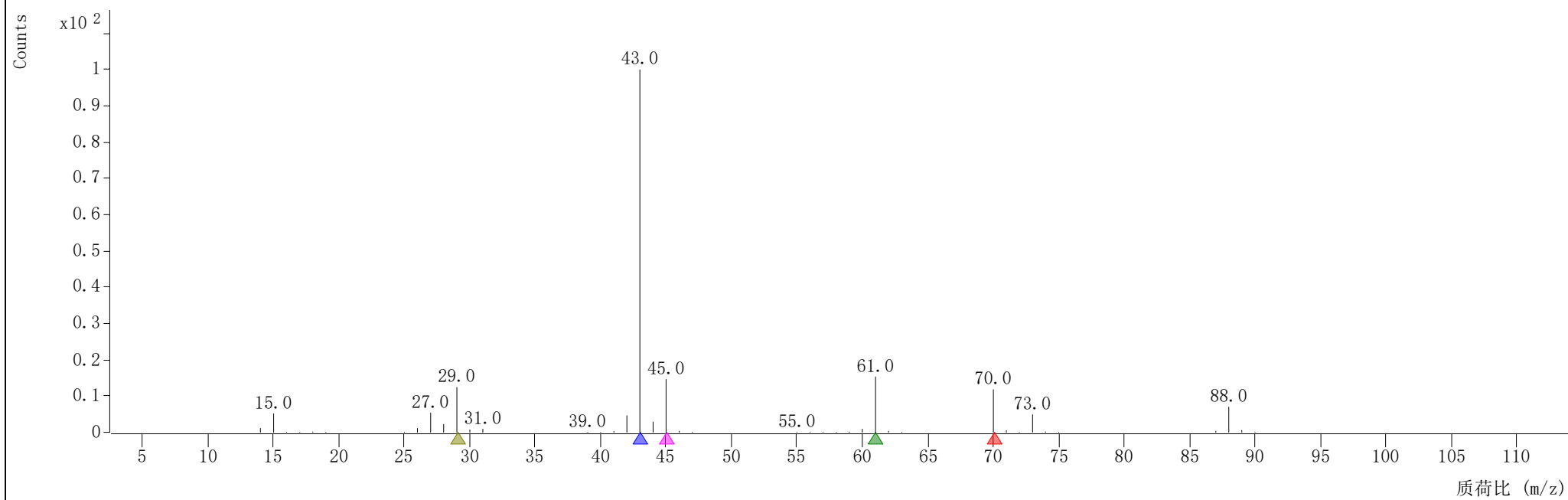

Supplement: Supplementary file 22 — Figure S22. Mass Spectrometry of Ethyl acetate [file mmc22.pdf]

组分 RT: 9.4296

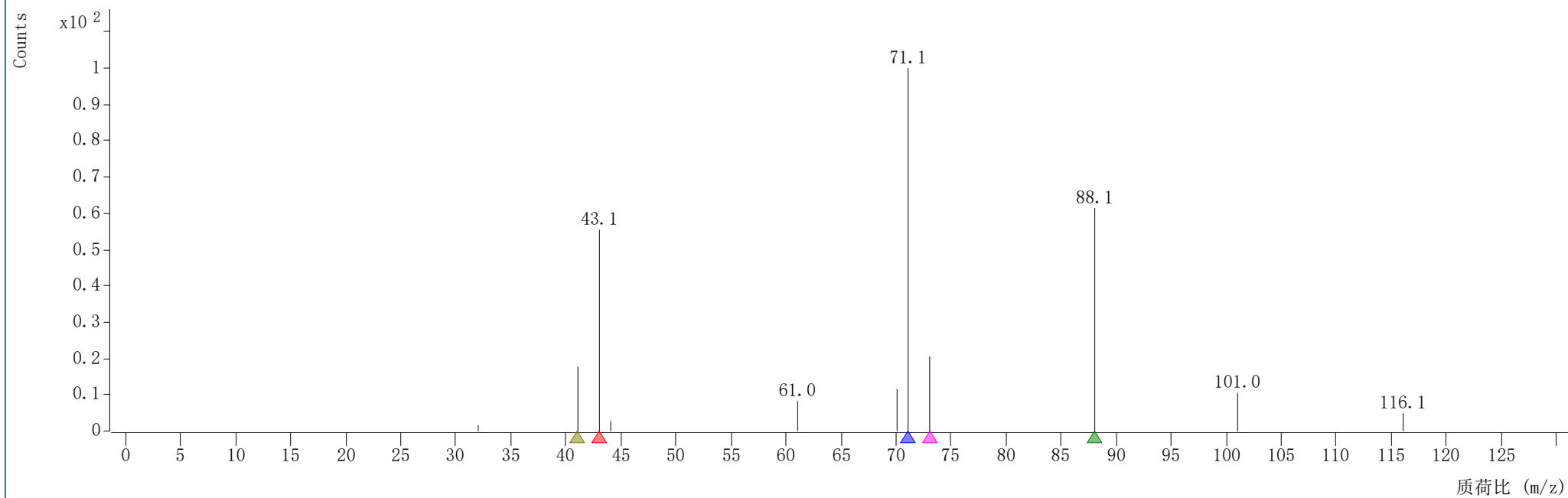

Butanoic acid, ethyl ester (NIST17.L)

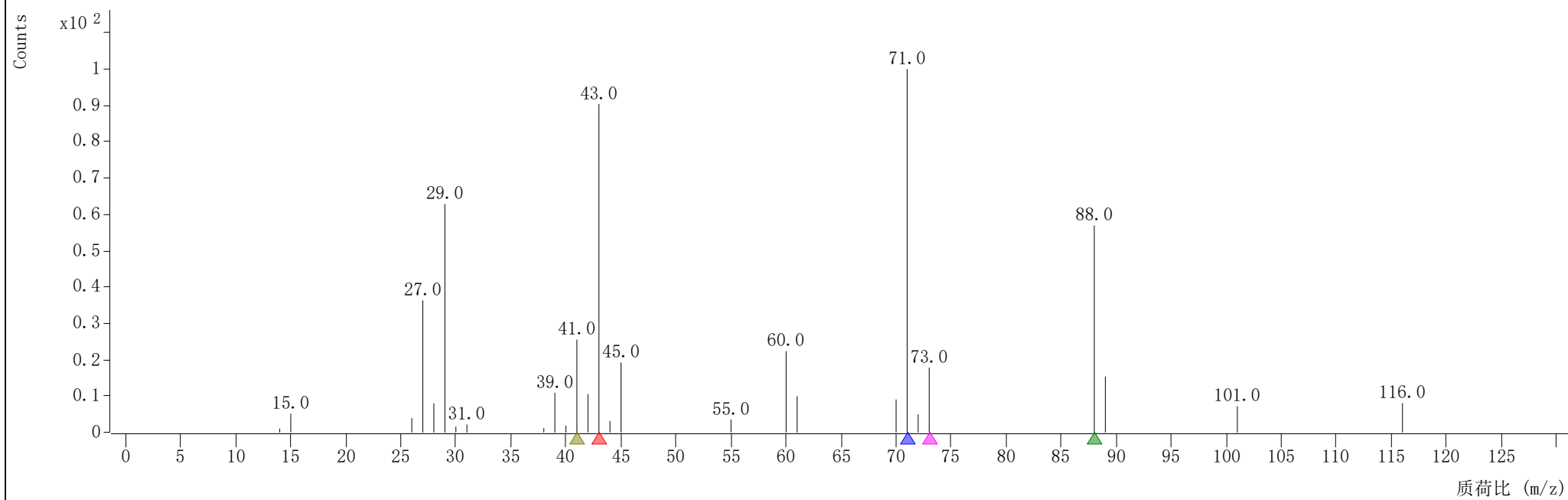

Supplement: Supplementary file 23 — Figure S23. Mass Spectrometry of Ethyl butyrate [file mmc23.pdf]

组分 RT: 33.0760

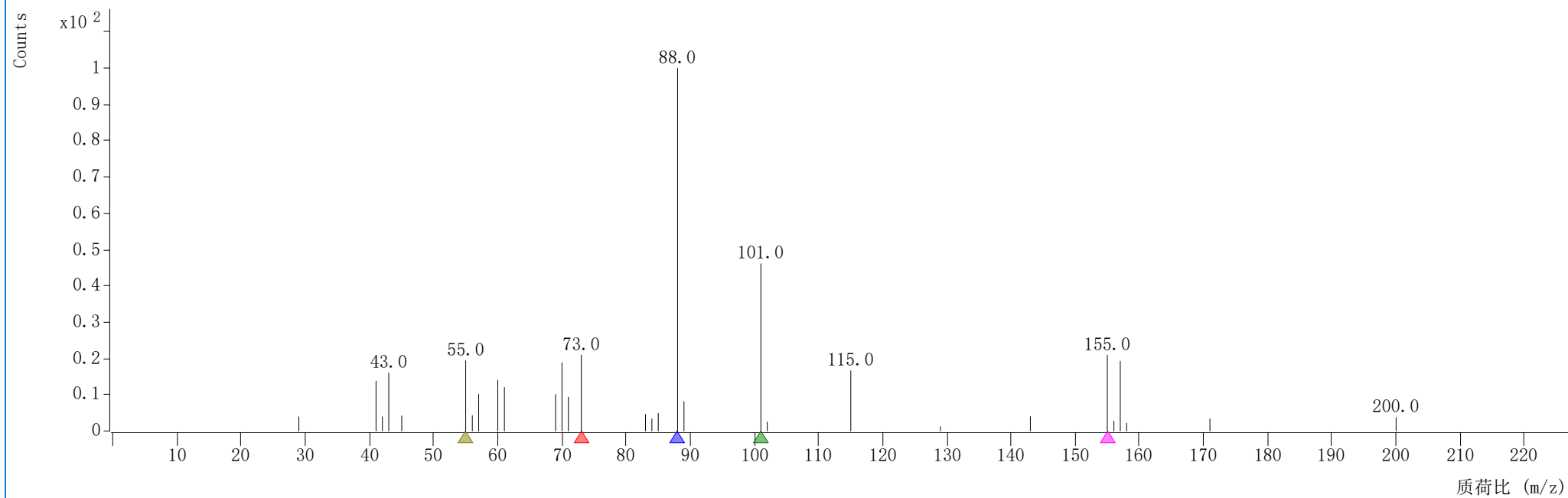

Decanoic acid, ethyl ester (NIST17.L)

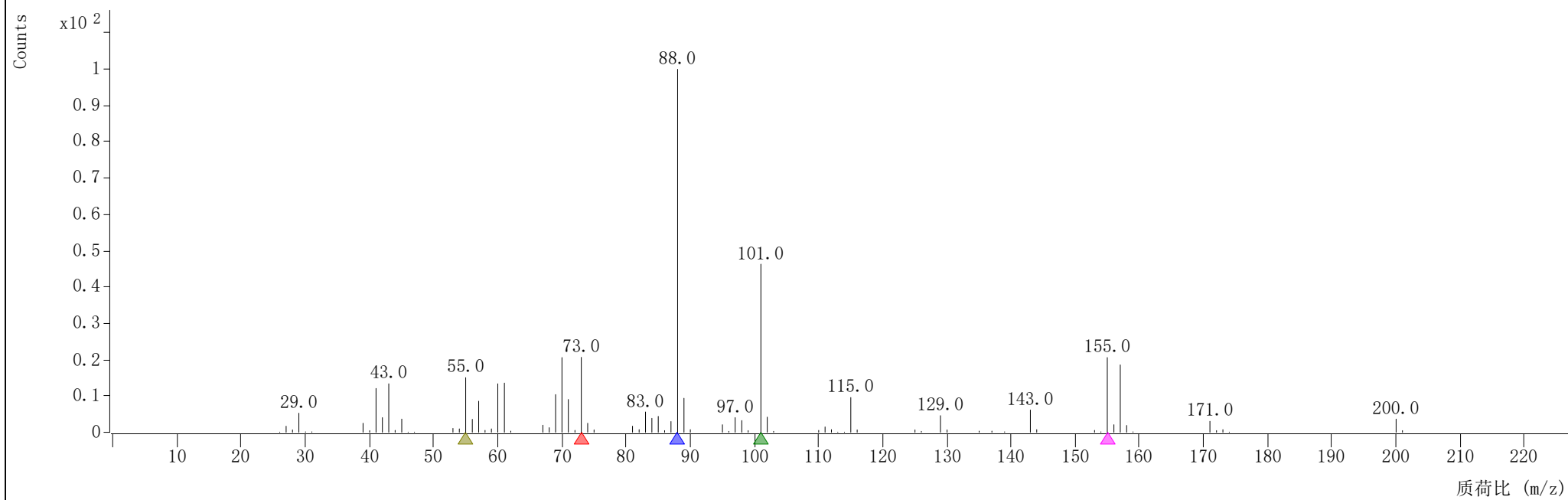

Supplement: Supplementary file 24 — Figure S24. Mass Spectrometry of Ethyl decanoate [file mmc24.pdf]

组分 RT: 40.6904

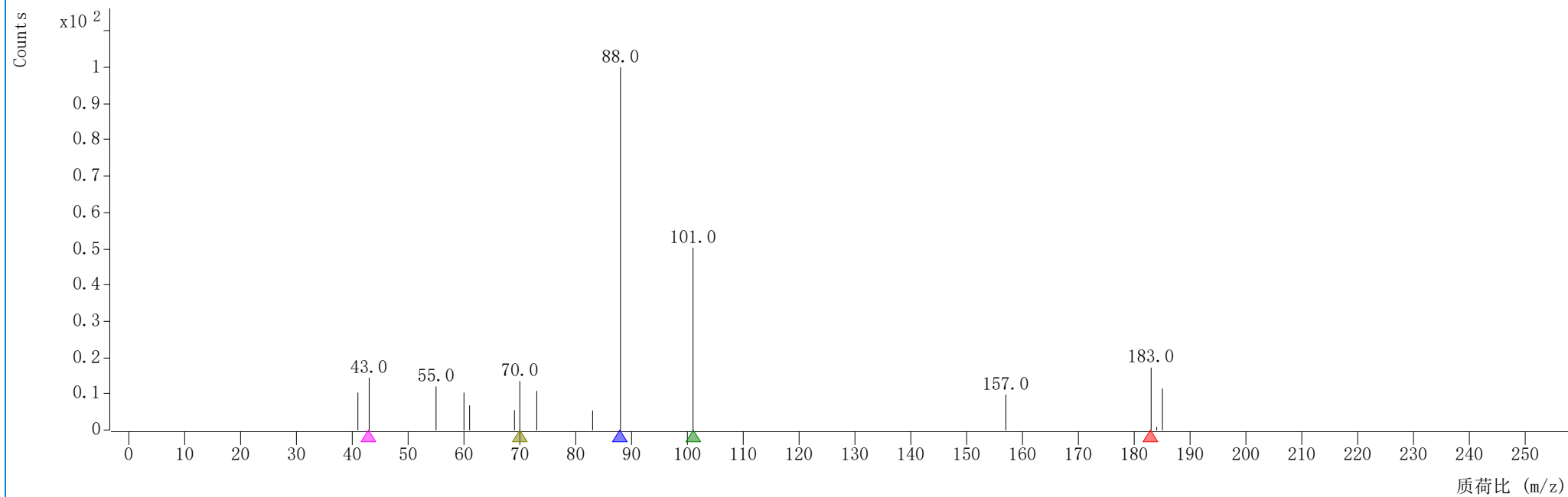

Dodecanoic acid, ethyl ester (NIST17.L)

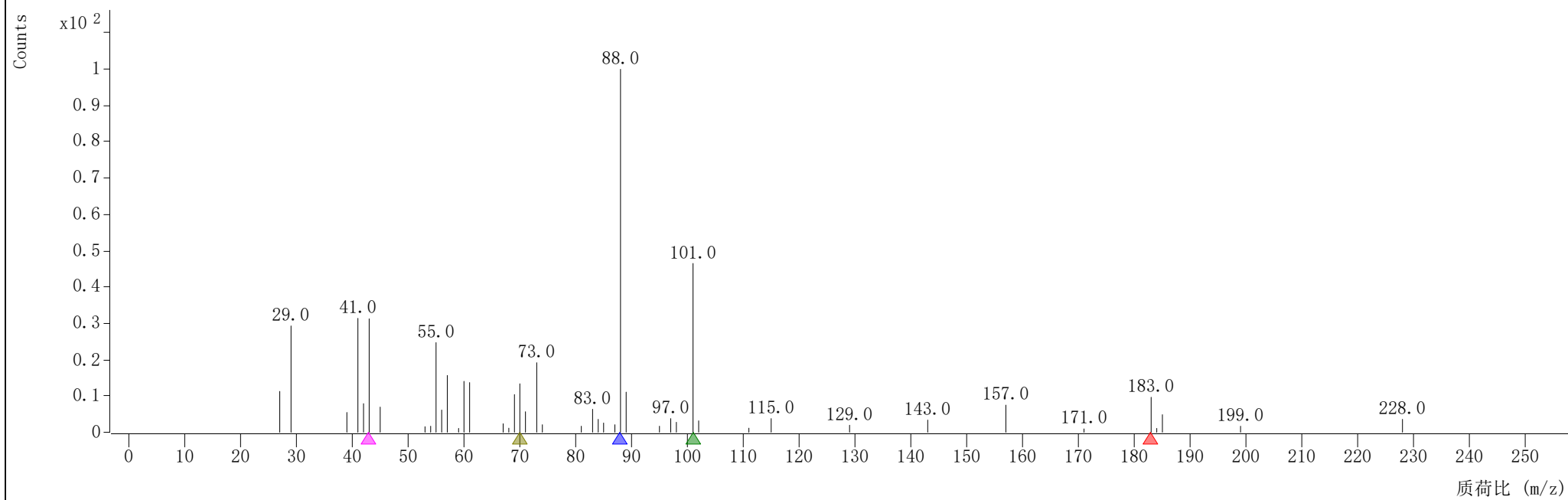

Supplement: Supplementary file 25 — Figure S25. Mass Spectrometry of Ethyl dodecanoate [file mmc25.pdf]

组分 RT: 54.0116

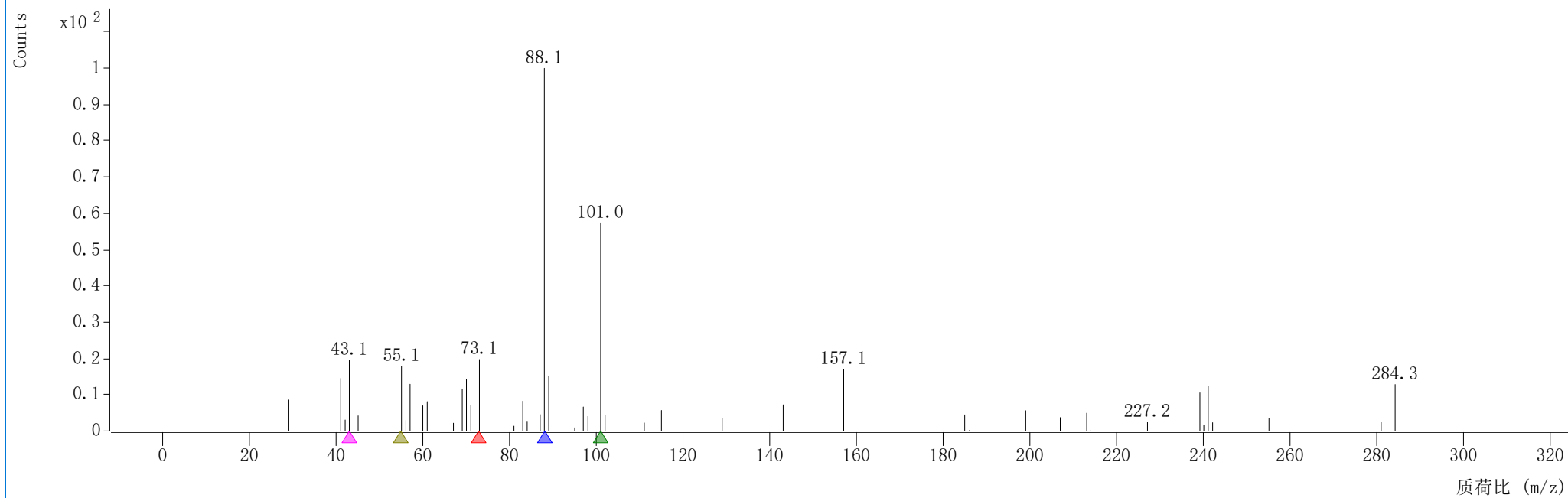

Hexadecanoic acid, ethyl ester (NIST17.L)

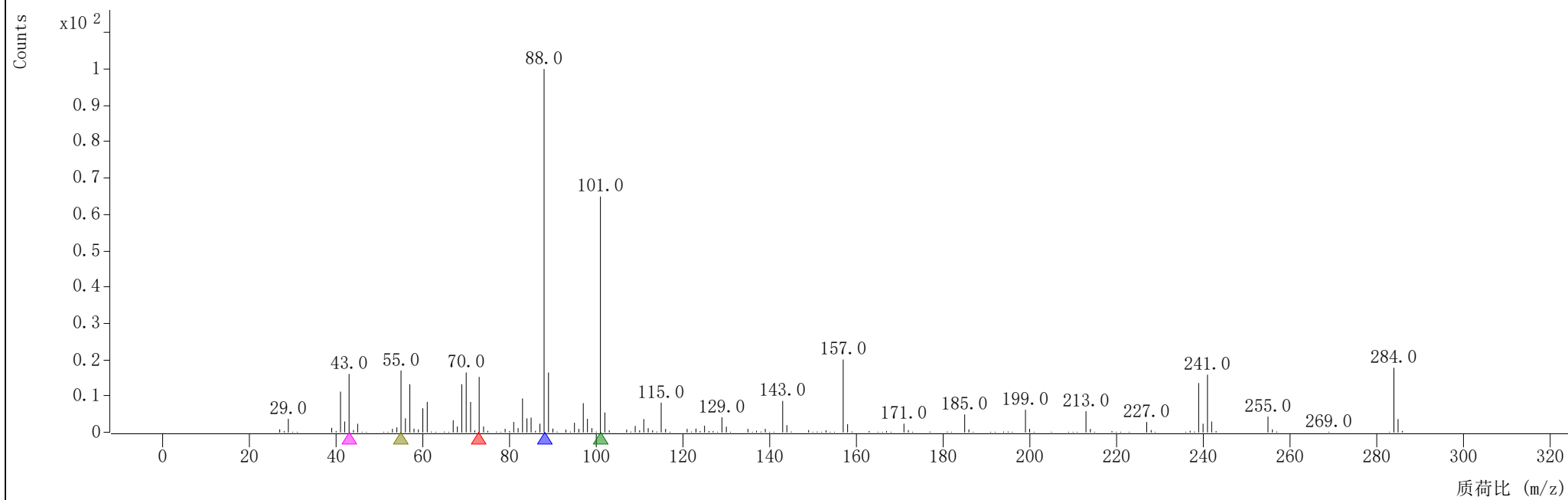

Supplement: Supplementary file 26 — Figure S26. Mass Spectrometry of Ethyl hexadecanoate [file mmc26.pdf]

组分 RT: 16.4102

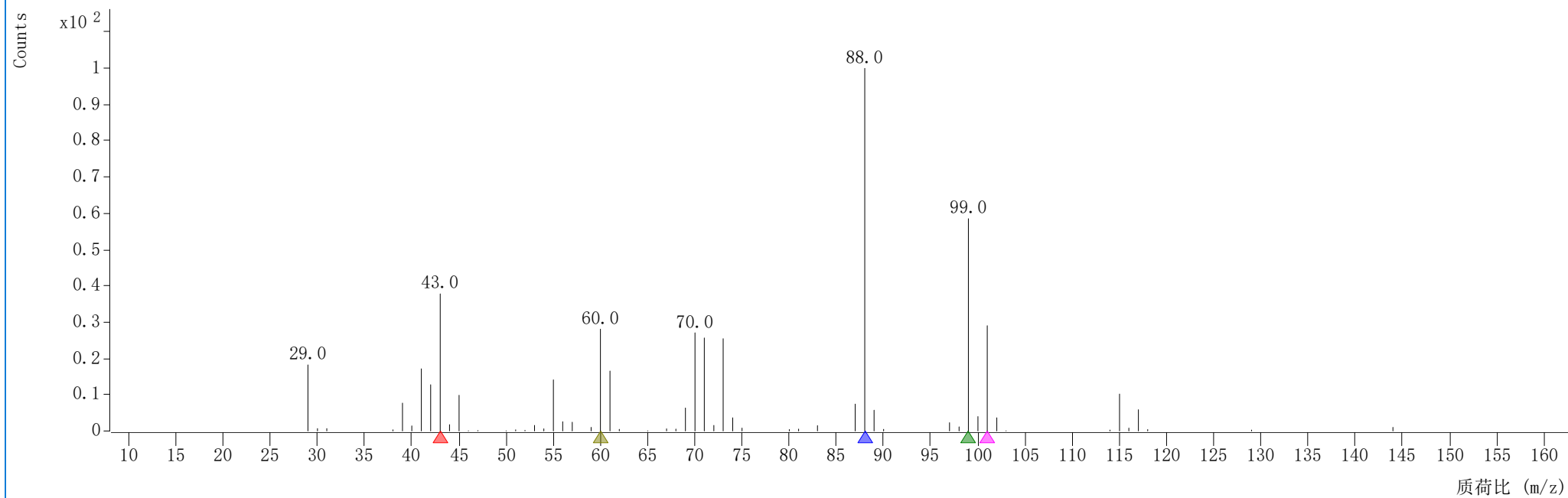

Hexanoic acid, ethyl ester (NIST17.L)

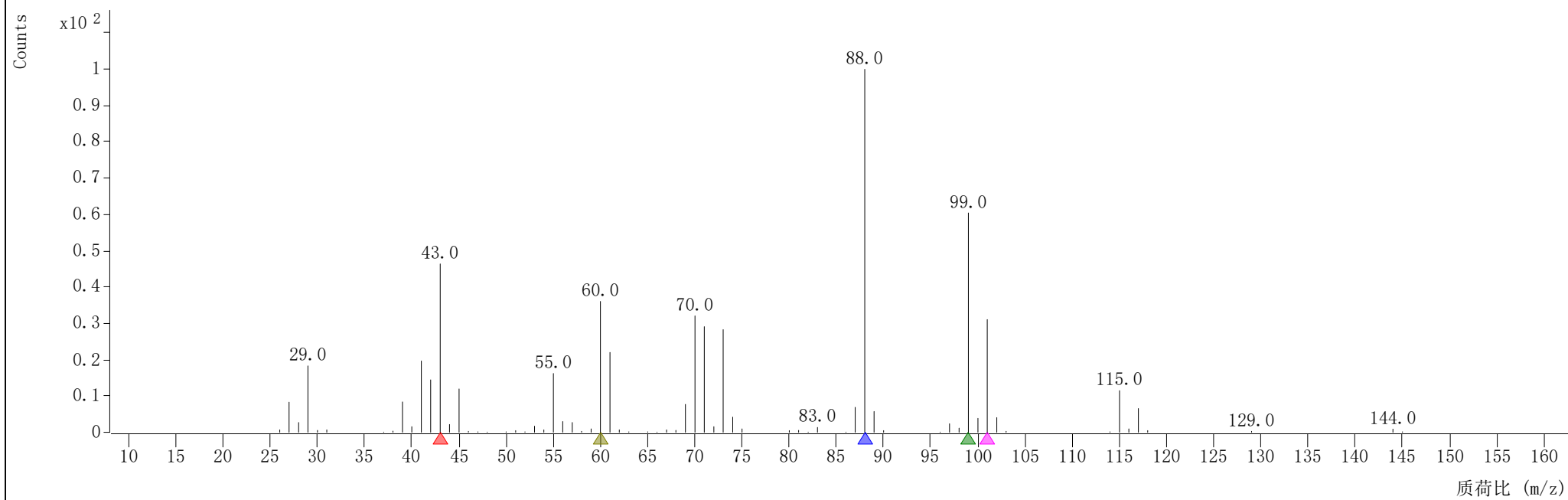

Supplement: Supplementary file 27 — Figure S27. Mass Spectrometry of Ethyl hexanoate [file mmc27.pdf]

组分 RT: 21.0178

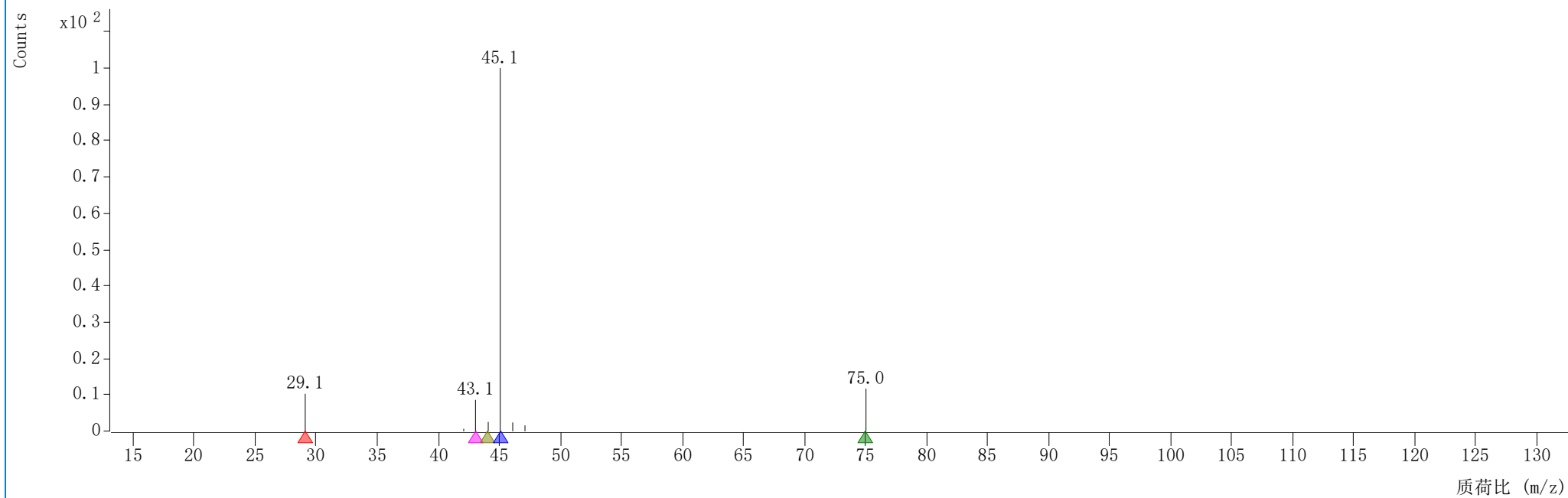

Propanoic acid, 2-hydroxy-, ethyl ester (NIST17.L)

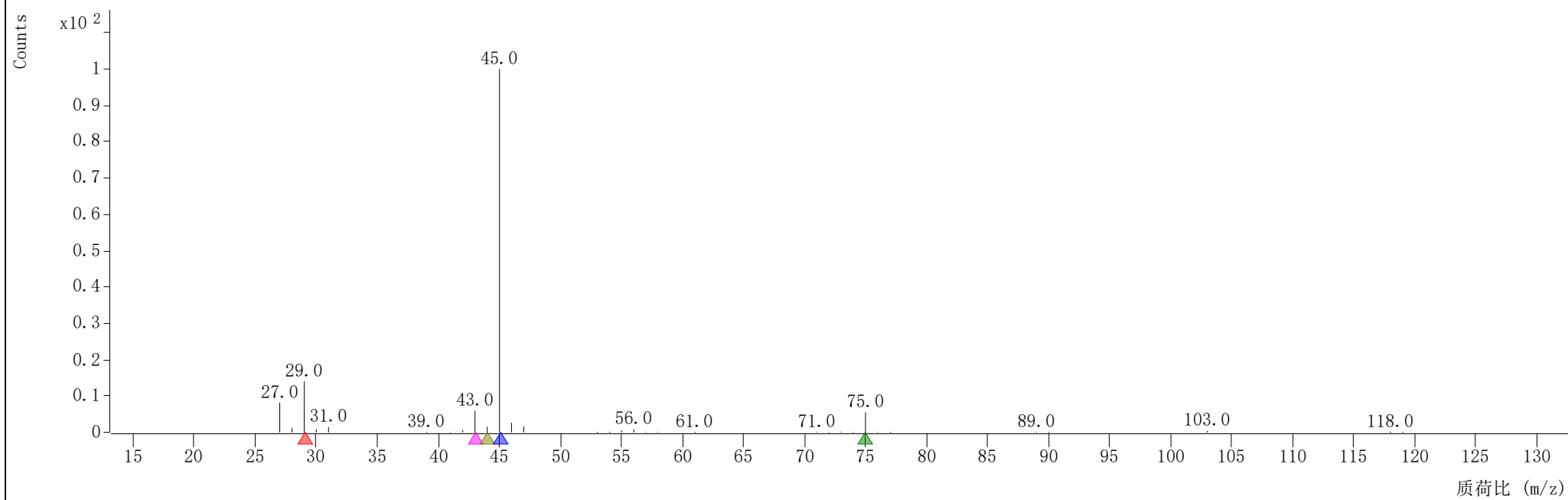

Supplement: Supplementary file 28 — Figure S28. Mass Spectrometry of Ethyl lactate [file mmc28.pdf]

组分 RT: 24.8337

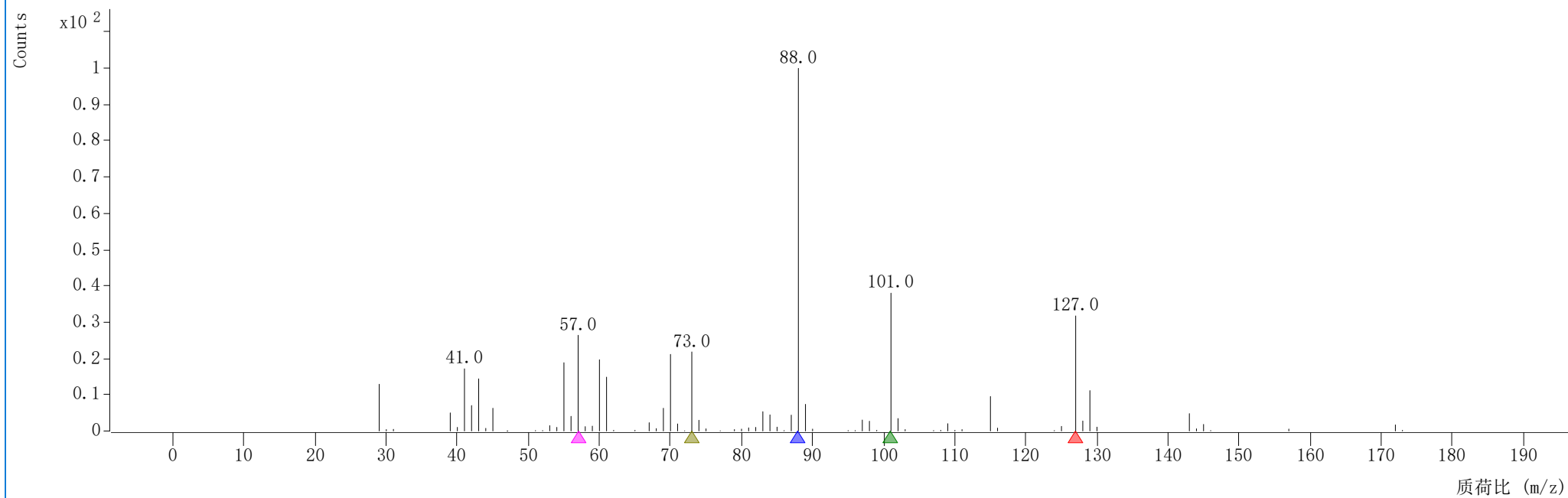

Octanoic acid, ethyl ester (NIST17.L)

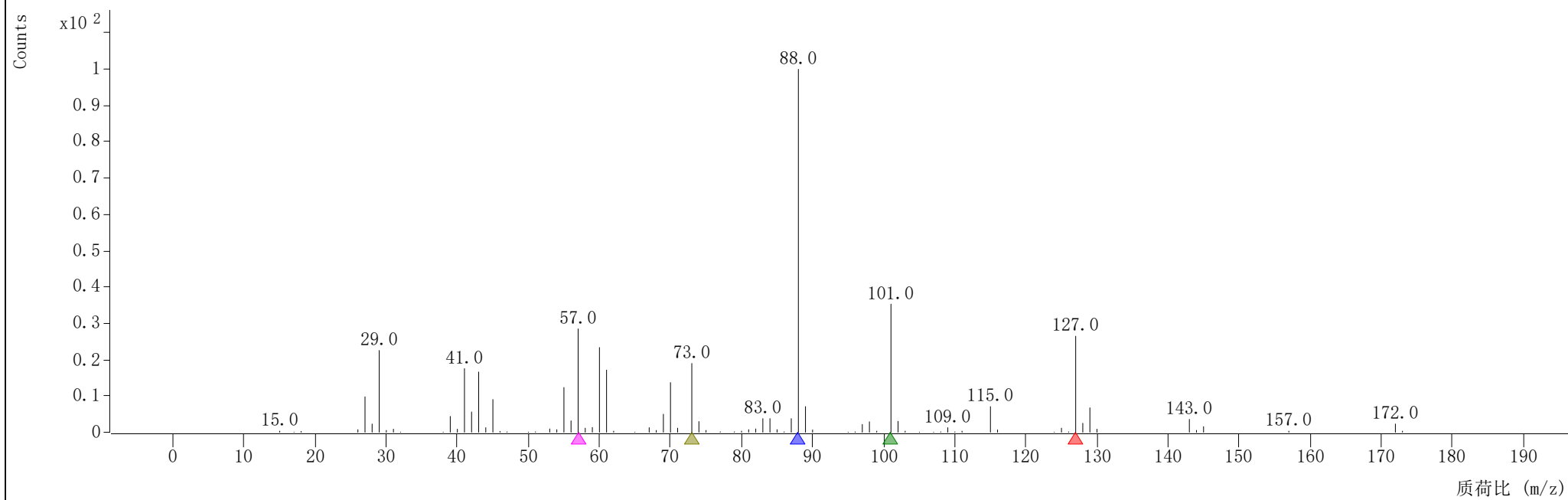

Supplement: Supplementary file 29 — Figure S29. Mass Spectrometry of Ethyl octanoate [file mmc29.pdf]

组分 RT: 26.3975

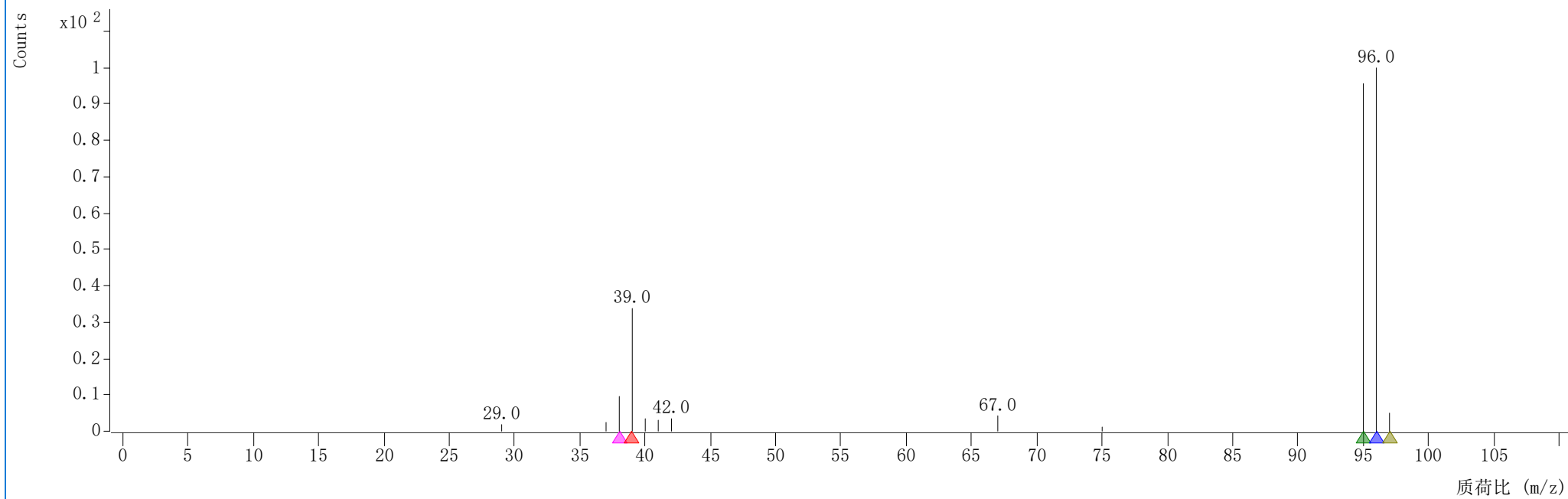

Furfural (NIST17.L)

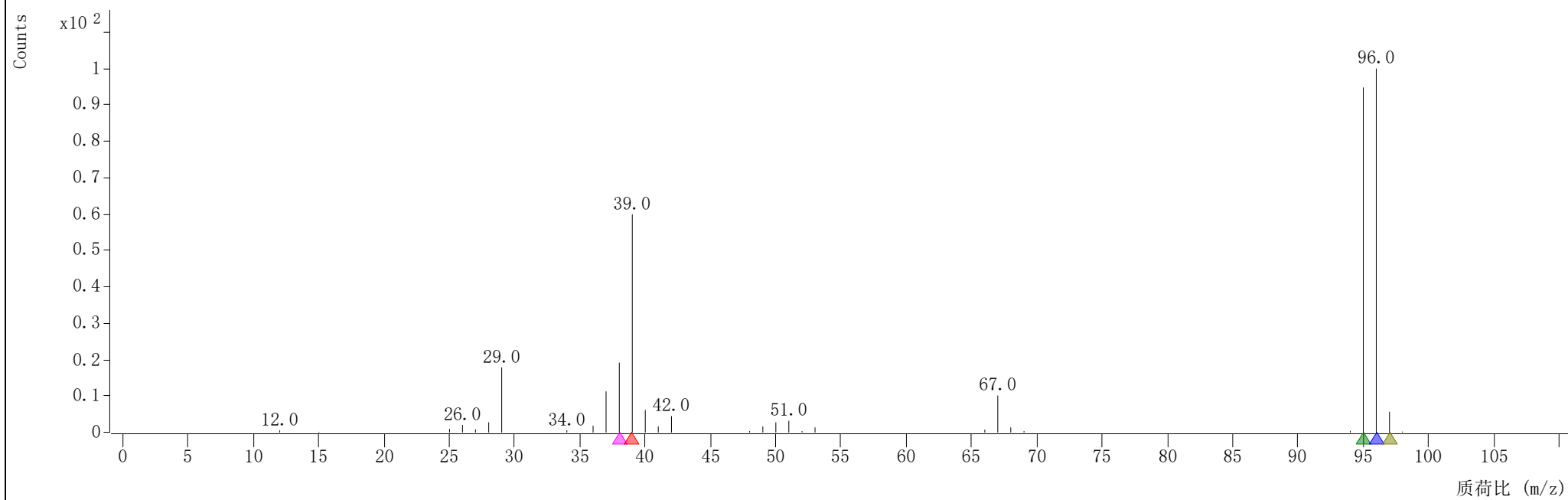

Supplement: Supplementary file 30 — Figure S30. Mass Spectrometry of Furfural [file mmc30.pdf]

组分 RT: 30.4000

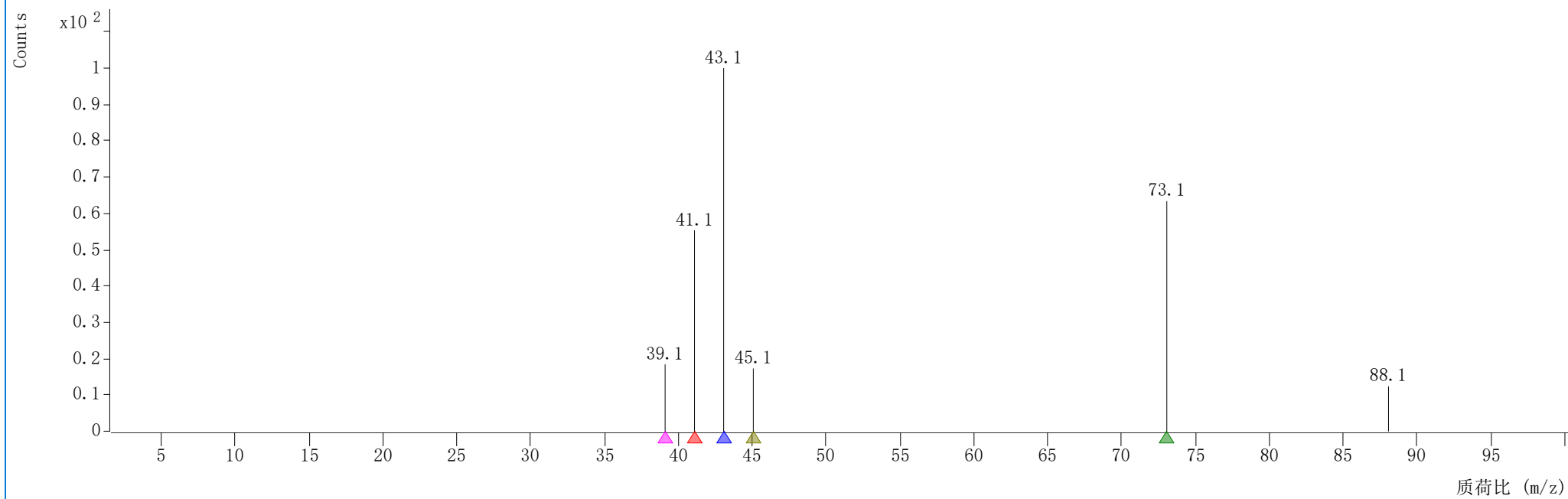

Propanoic acid, 2-methyl- (NIST17.L)

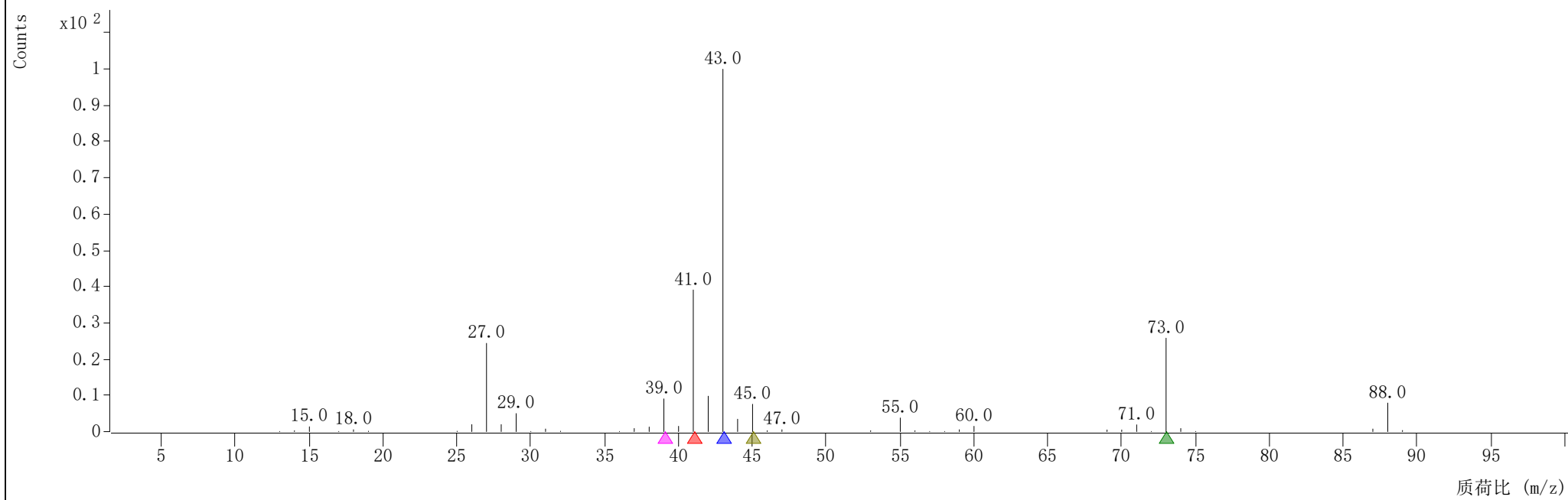

Supplement: Supplementary file 31 — Figure S31. Mass Spectrometry of Isobutyric acid [file mmc31.pdf]

组分 RT: 48.0328

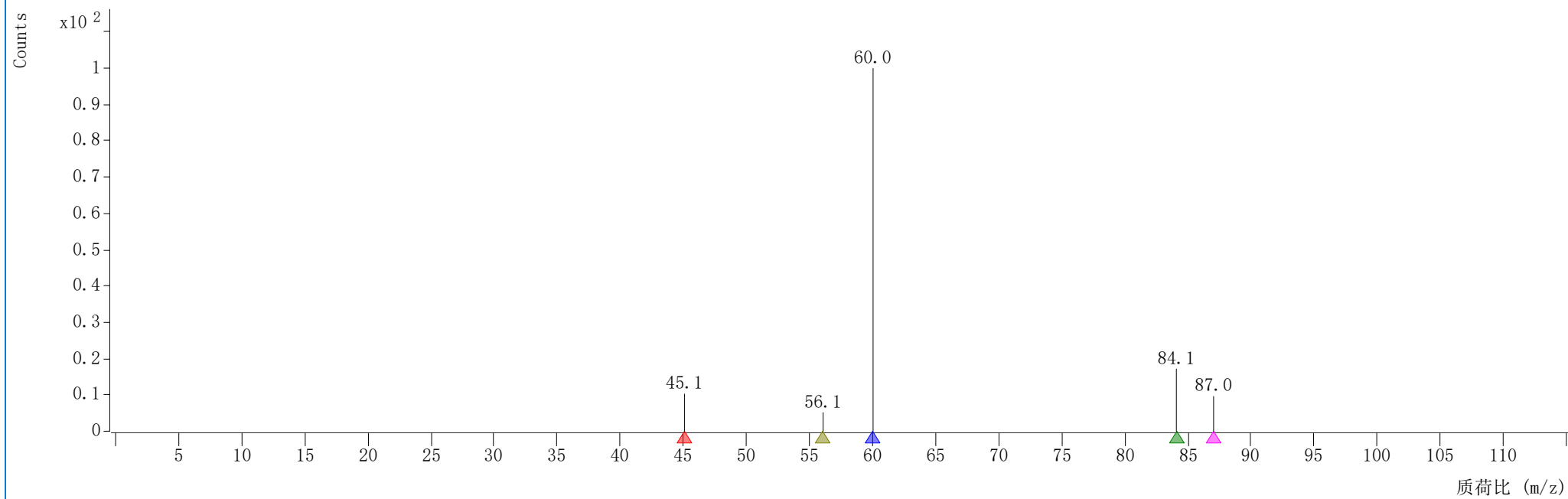

Butanoic acid, 3-methyl- (NIST17.L)

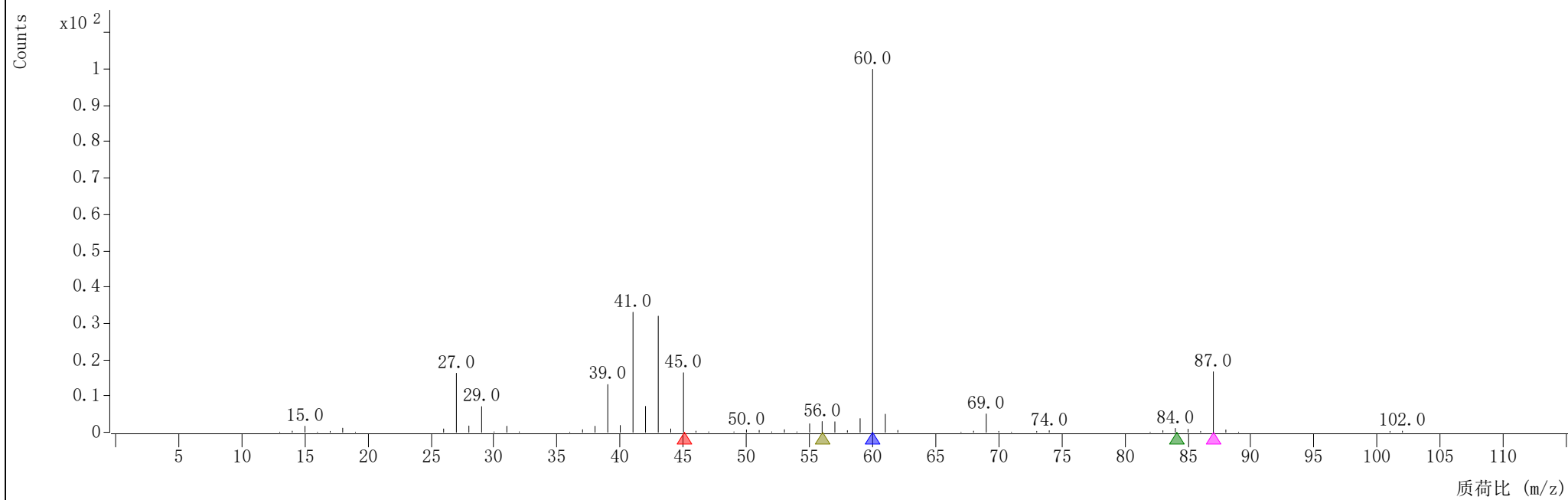

Supplement: Supplementary file 32 — Figure S32. Mass Spectrometry of Isovaleric acid [file mmc32.pdf]

组分 RT: 22.9764

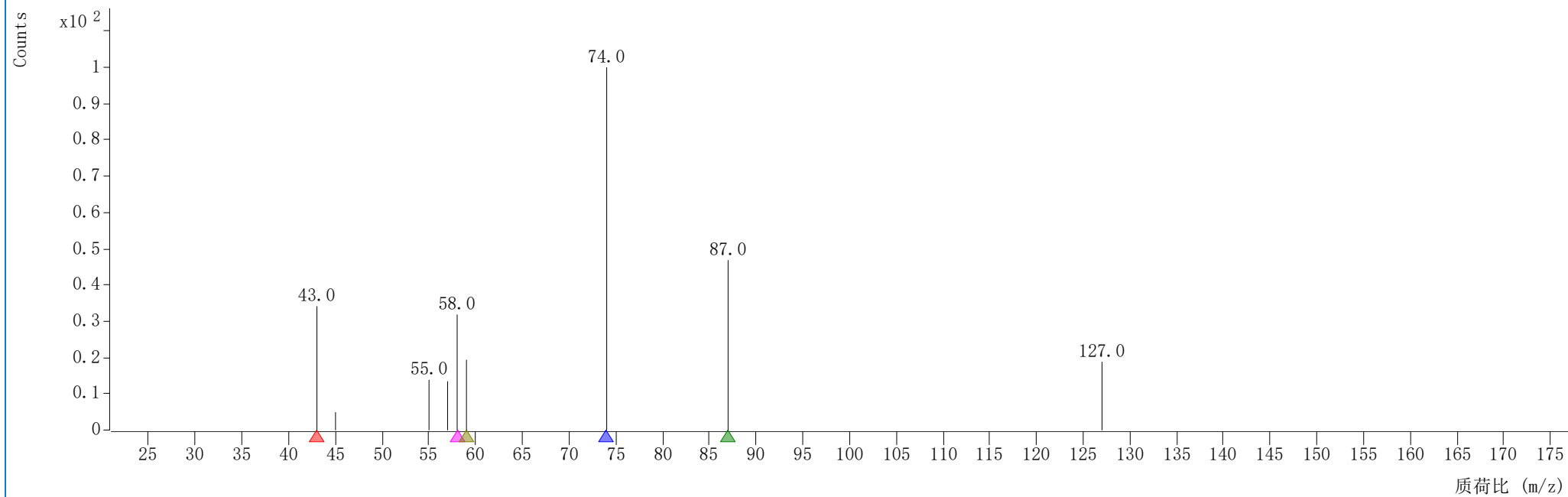

Octanoic acid, methyl ester (NIST17.L)

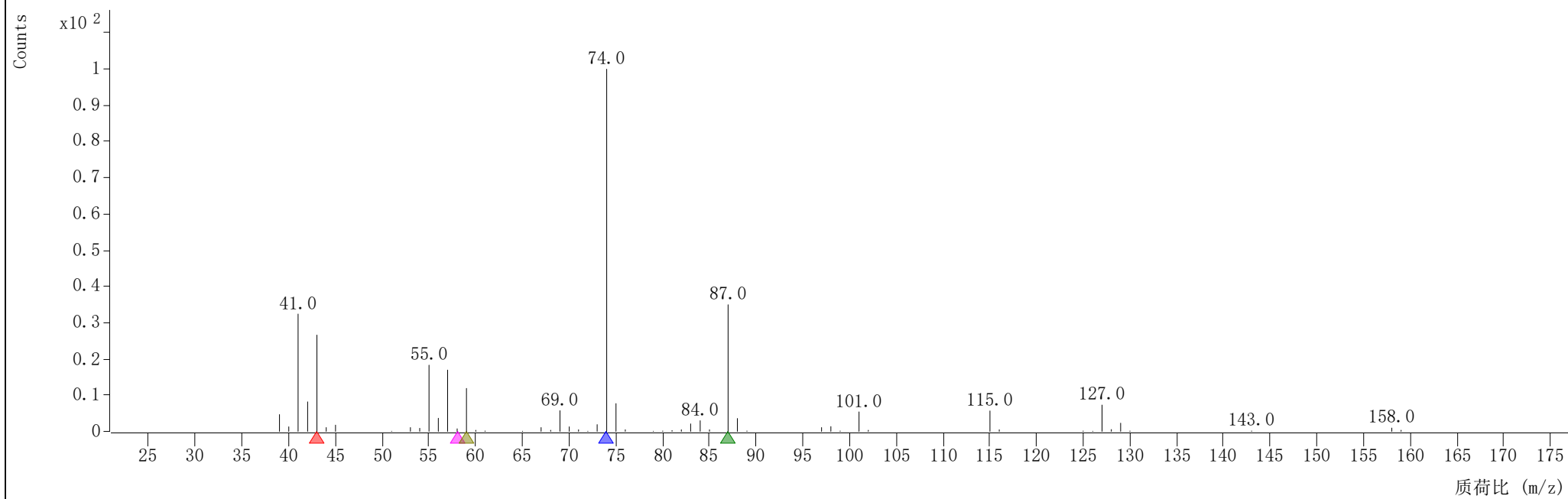

Supplement: Supplementary file 33 — Figure S33. Mass Spectrometry of Methyl octanoate [file mmc33.pdf]

组分 RT: 40.0220

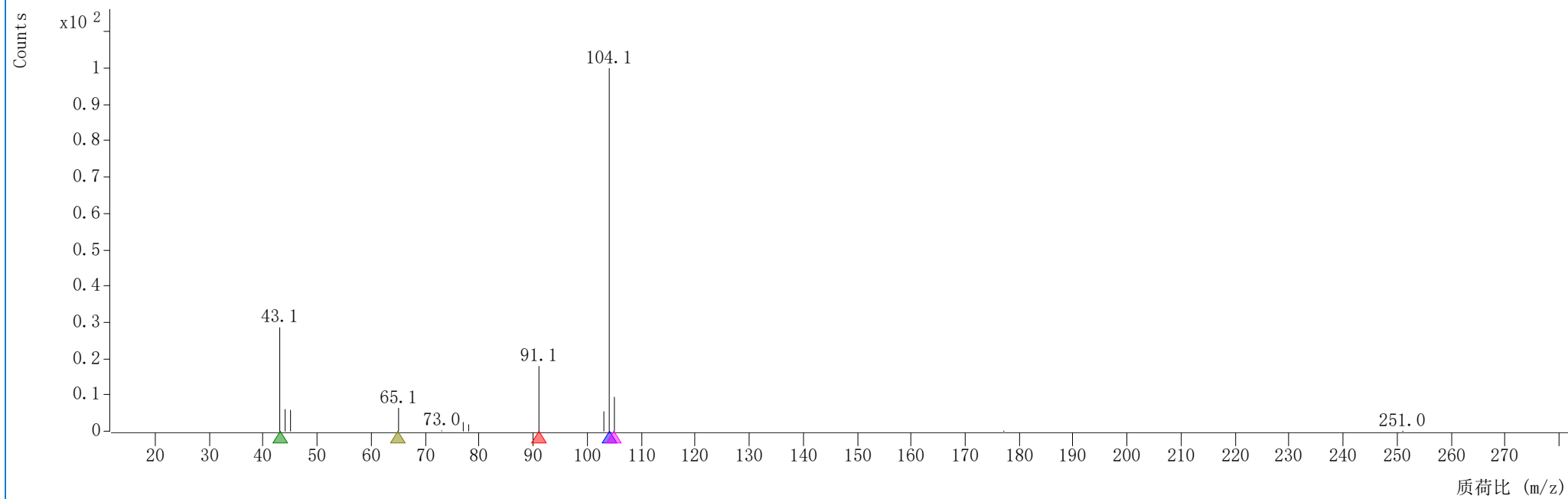

Acetic acid, 2-phenylethyl ester (NIST17.L)

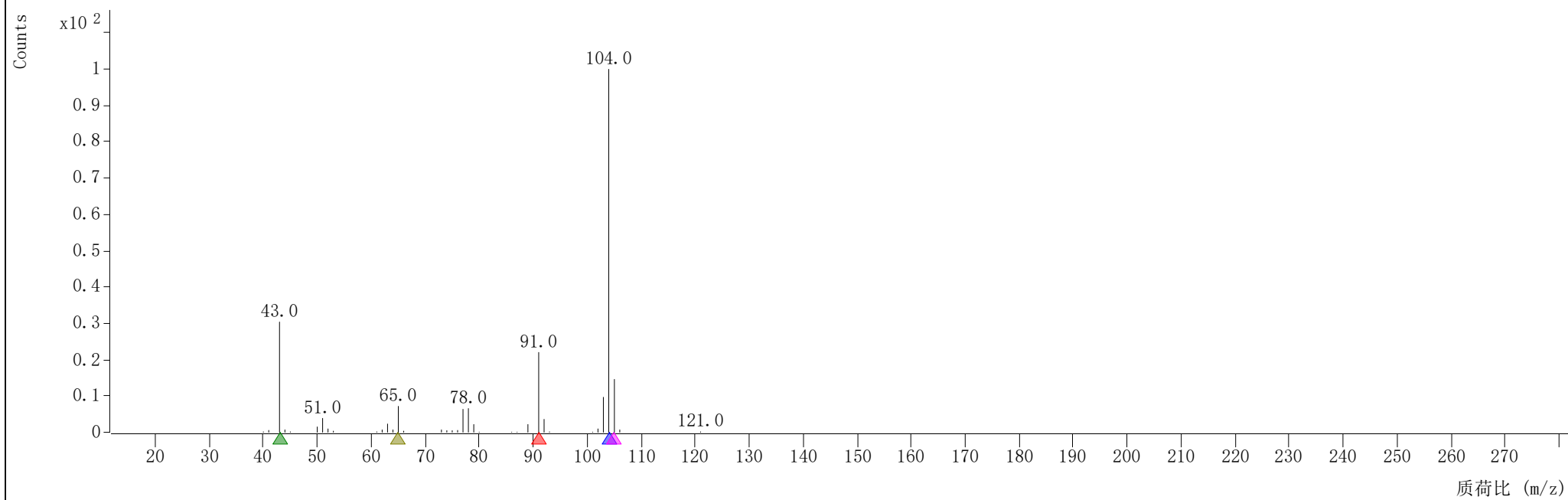

Supplement: Supplementary file 34 — Figure S34. Mass Spectrometry of Phenethyl acetate [file mmc34.pdf]

组分 RT: 43.3412

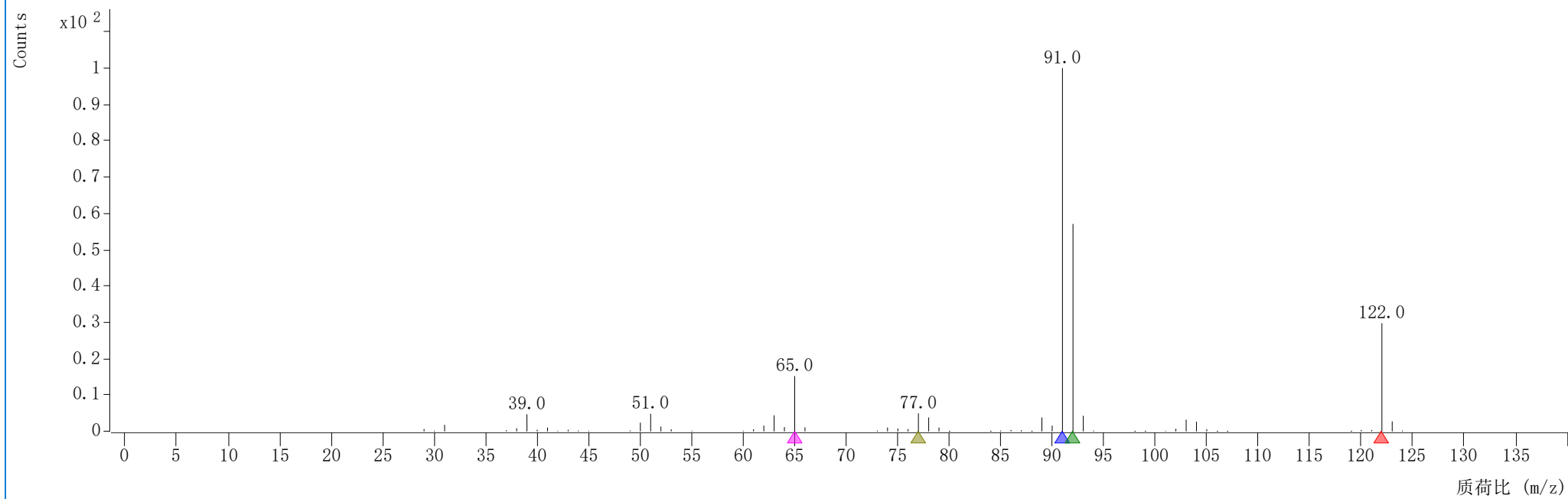

Phenylethyl Alcohol (NIST17.L)

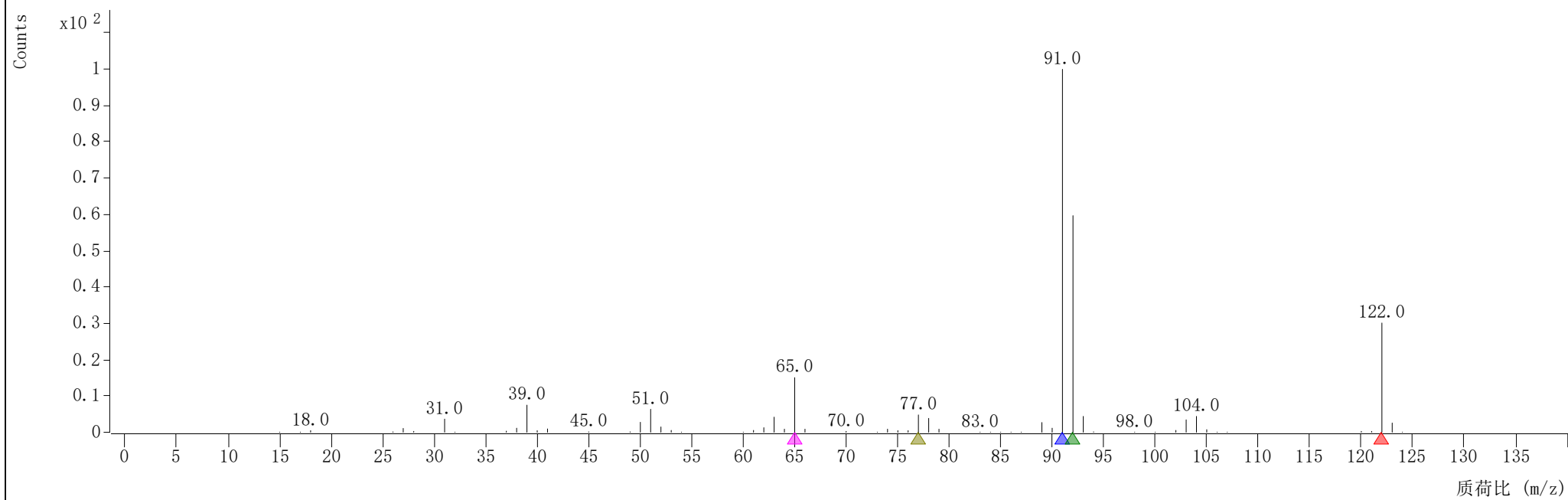

Supplement: Supplementary file 35 — Figure S35. Mass Spectrometry of Phenylethyl alcohol [file mmc35.pdf]

组分 RT: 35.4398

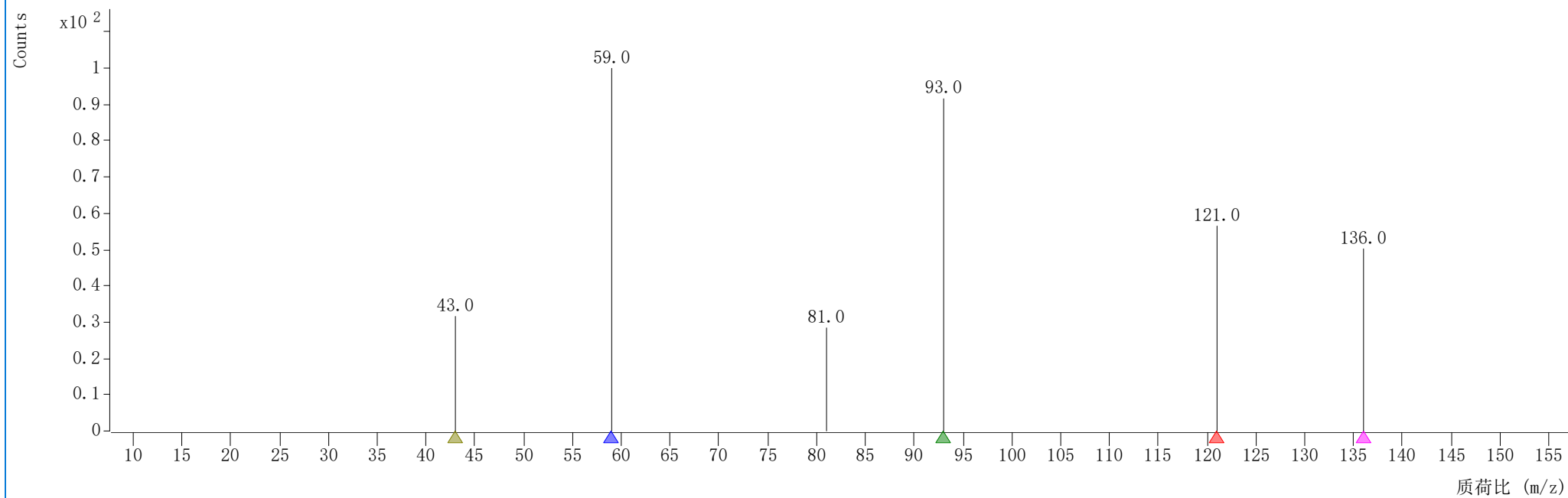

L- $\alpha$ -Terpineol (NIST17.L)

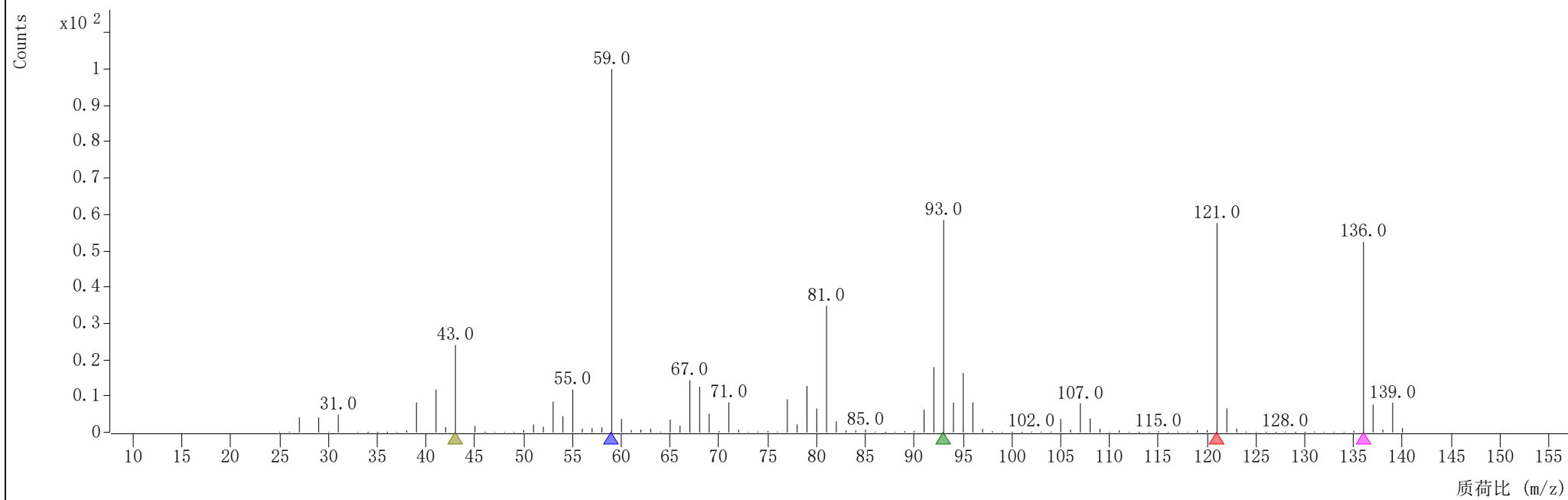

Supplement: Supplementary file 36 — Figure S36. Mass Spectrometry of α-Terpineol [file mmc36.pdf]
